# Organometallic Chemistry within the Structured Environment Provided by the Macrocyclic Cores of Carbaporphyrins and Related Systems

**DOI:** 10.3390/molecules28031496

**Published:** 2023-02-03

**Authors:** Timothy D. Lash

**Affiliations:** Department of Chemistry, Illinois State University, Normal, IL 61790-4160, USA; tdlash@ilstu.edu

**Keywords:** porphyrinoids, carbaporphyrins, azuliporphyrins, benziporphyrins, organometallic complexes, rearrangements, oxidations, aromaticity

## Abstract

The unique environment within the core of carbaporphyrinoid systems provides a platform to explore unusual organometallic chemistry. The ability of these structures to form stable organometallic derivatives was first demonstrated for N-confused porphyrins but many other carbaporphyrin-type systems were subsequently shown to exhibit similar or complementary properties. Metalation commonly occurs with catalytically active transition metal cations and the resulting derivatives exhibit widely different physical, chemical and spectroscopic properties and range from strongly aromatic to nonaromatic and antiaromatic species. Metalation may trigger unusual, highly selective, oxidation reactions. Alkyl group migration has been observed within the cavity of metalated carbaporphyrins, and in some cases ring contraction of the carbocyclic subunit takes place. Over the past thirty years, studies in this area have led to multiple synthetic routes to carbaporphyrinoid ligands and remarkable organometallic chemistry has been reported. An overview of this important area is presented.

## 1. Introduction

**Figure 1:**
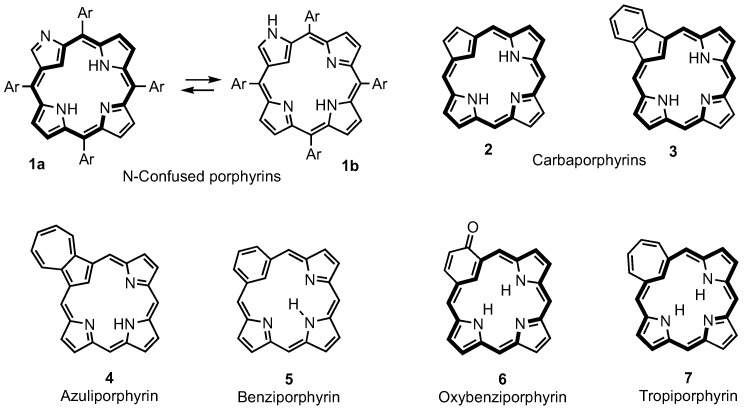
Selected carbaporphyrinoids.

Porphyrins are extraordinarily effective ligands that form coordination complexes to virtually every metal or metalloid element [1]. Although this versatility may be diminished upon core modification, porphyrin analogues still give remarkably diverse metalated derivatives [2]. When one or more of the nitrogen atoms within the porphyrin core are replaced by carbon atoms, the resulting carbaporphyrins [3] commonly form organometallic derivatives with late transition metals, including catalytically important cations such as nickel(II), palladium(II), platinum(II), silver(III) and gold(III) [4]. In these systems, the metal cation is constrained within a highly ordered coordination sphere, and this can lead to unusual reactivity, including selective oxidation reactions. The best known porphyrinoids of this type are the so-called N-confused porphyrins (NCP, **1**) [5,6,7,8,9,10,11,12], and these can easily be prepared by a one-pot procedure from pyrrole and benzaldehyde [13,14]. However, many other intriguing carbaporphyrinoid systems such as carbaporphyrins **2** and **3** [15], azuliporphyrins **4** [16], benziporphyrins **5** [17,18], oxybenziporphyrins **6** [19], and tropiporphyrins **7** [20] have been reported (Figure 1) and these exhibit diverse structural and spectroscopic properties, unusual reactivity, and varying degrees of aromatic, nonaromatic and antiaromatic characteristics. Carbaporphyrinoids have attracted widespread interest and have been the subject of a number of reviews [19,20,21,22,23,24,25,26,27,28,29,30]. This article focuses on the formation and reactivity of metalated carbaporphyrinoids that have carbon-metal bonds within 16-atom macrocyclic cavities. Methods used to prepare these fascinating ligands will be briefly discussed and the reactivity of different families of carbaporphyrinoids will be presented. N-Confused porphyrins are included in these discussions but will be covered in less depth as this area has been covered in some detail elsewhere [5,6,7,8,9,10,11,12]. Contracted and expanded systems are also briefly discussed.

## 2. Synthetic Routes to Carbaporphyrinoid Systems

**Scheme 1:**
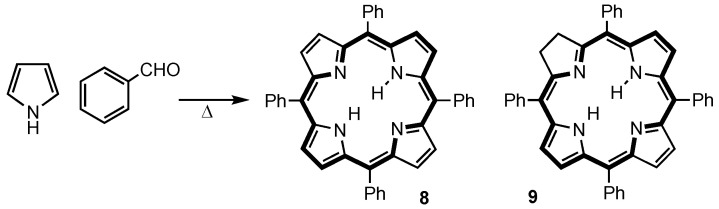
Rothemund synthesis of *meso*-tetraphenylporphyrin and a chlorin byproduct.

**Scheme 2:**
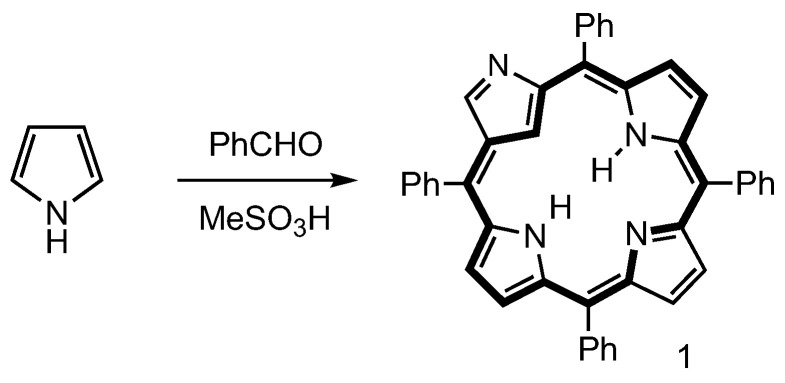
Synthesis of Tetraphenyl N-Confused Porphyrin.

Although N-confused porphyrins (NCPs) were first reported in 1994 [31,32], speculations on the formation of porphyrin analogues with inverted pyrrole units were made over 50 years earlier. Calvin and coworkers speculated that by-products formed in the Rothemund reaction could correspond to isomers of *meso*-tetraphenylporphyrin **8** (TPP, Scheme 1) in which one or two of the pyrrole rings have been inverted, although some more bizarre suggestions were also made [33]. Subsequently, the major by-product in the synthesis of TPP from pyrrole and benzaldehyde was shown to be the related chlorin **9** [34], and the reported UV-vis spectra for Calvin’s porphyrin-like fractions do not resemble the spectra later obtained for NCP [31,32]. Pauling also speculated about the existence of this type of porphyrin isomer around the same time, although these musings were not published until they were discovered among his papers in 2011 [35]. These prescient speculations had been all but forgotten when Latos-Grażyński and Furuta, in 1994, independently isolated modest yields of NCPs from reactions of aromatic aldehydes with pyrrole under acid-catalyzed conditions. Lindsey, Geier and coworkers later developed an efficient synthesis of tetraphenyl NCP with yields of up to 40% using methanesulfonic acid as the catalyst (Scheme 2) [13,14]. Early investigation by Latos-Grażyński, Furuta and others demonstrated the propensity of this system to form organometallic derivatives [5,6,7,8].

**Scheme 3:**
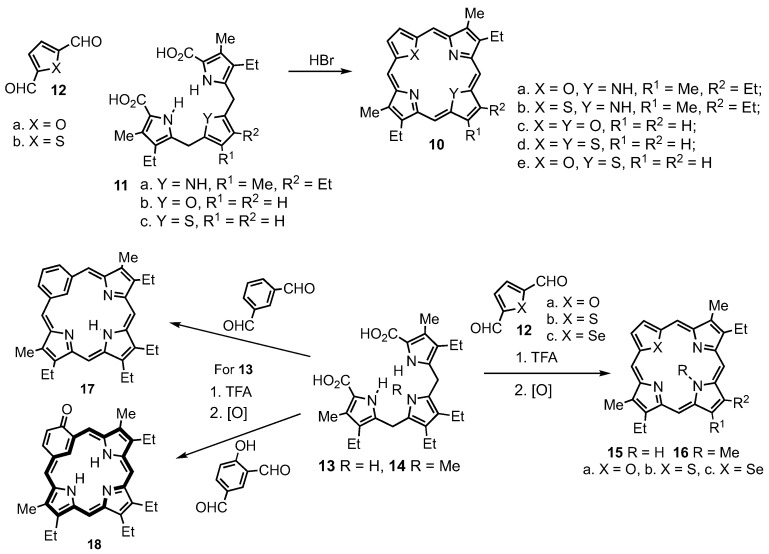
Example of MacDonald-type ‘3 + 1’ syntheses of porphyrin analogues.

**Scheme 4:**
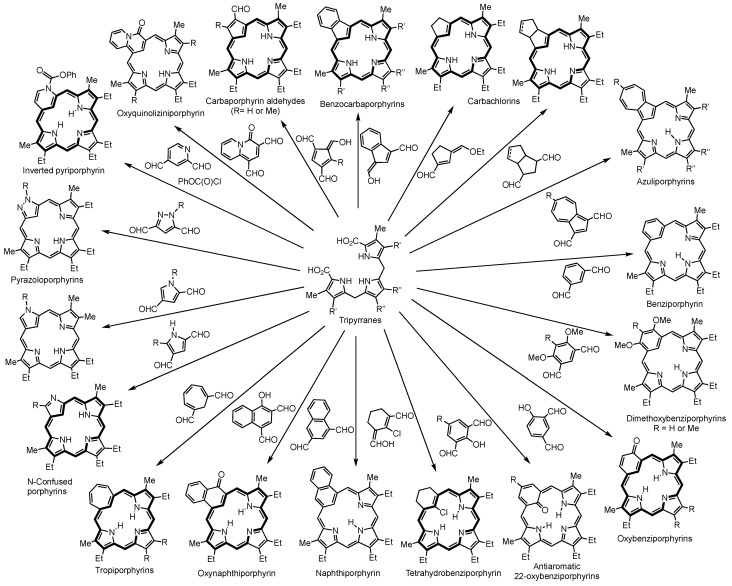
Synthesis of carbaporphyrinoid systems using MacDonald-type ‘3 + 1’ condensations.

**Scheme 5:**
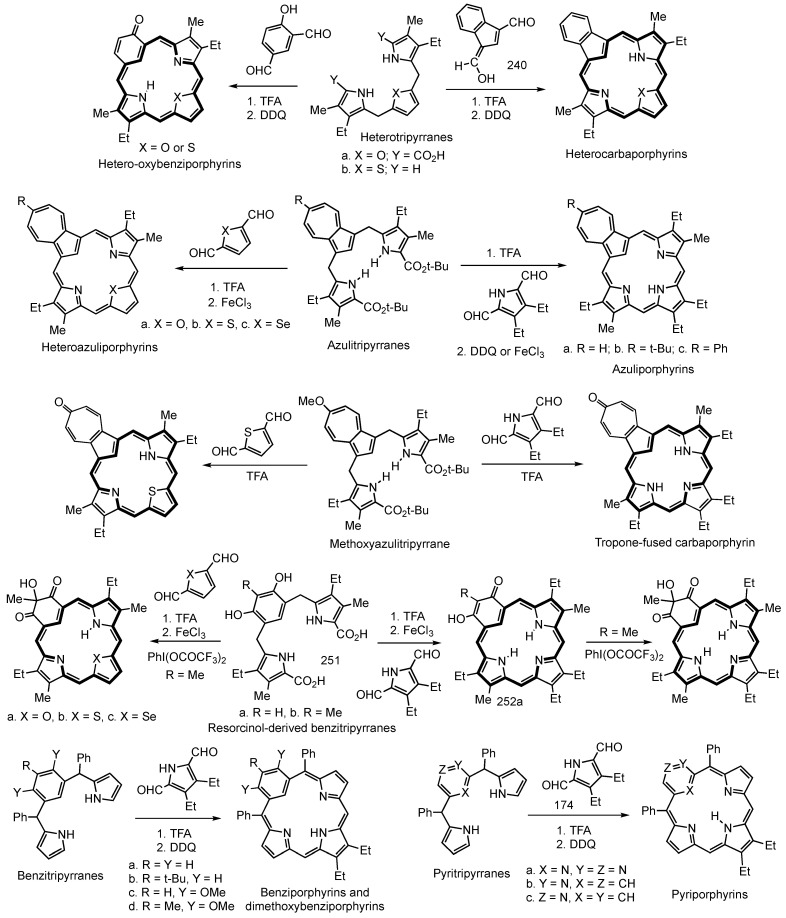
MacDonald-type ‘3 + 1’ syntheses of carbaporphyrinoids from tripyrrane analogues.

**Scheme 6:**
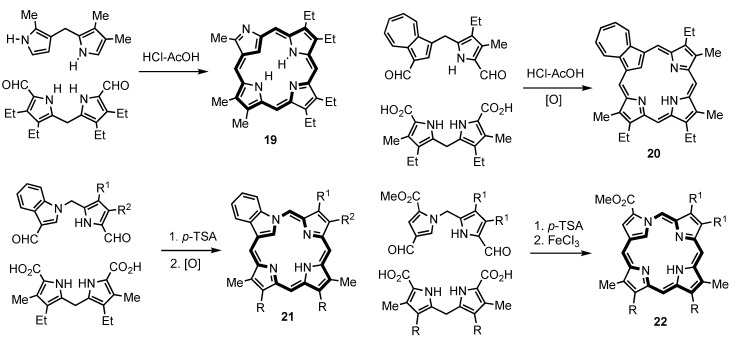
MacDonald ‘2 + 2’ syntheses of carbaporphyrinoids.

The ‘3 + 1’ variant of the MacDonald condensation provides an effective route to porphyrin analogues such as carbaporphyrins [36,37]. This approach was first used by Johnson to prepare 21-oxa- (**10a**), 21-thia- (**10b**), 21,23-dioxa- (**10c**), 21,23-dithia- (**10d**) and 21-oxa-23-thiaporphyrins (**10e**) (Scheme 3) [38]. The strategy involved reacting tripyrrane **11a** and related species **11b,c** with furan or thiophene dialdehydes **12** in the presence of HBr. Although this approach gave the first examples of core modified porphyrins, no further applications of the ‘3 + 1’ route were made for nearly 25 years. This was due in part to tripyrranes being relatively inaccessible at that time, although an efficient route to these intermediates was subsequently reported by Sessler in 1987 [39]. The ‘3 + 1’ strategy provided access to previously unknown heteroporphyrins, but thiaporphyrin **10b** was isolated as an isomeric mixture [38]. Johnson noted that even at 60 MHz, proton NMR spectroscopy showed that additional peaks were present, and this was attributed to “traces of other isomers formed by cleavage recombination reactions” [38]. Recently, syntheses of oxa-, thia- and selenaporphyrins from tripyrranes **13** and **14** and heterocyclic dialdehydes **12a**–**c** were reported using trifluoroacetic acid (TFA) as the catalyst, and this methodology afforded pure heteroporphyrins **15a**–**c** and *N*-methylheteroporphyrins **16a**–**c** that were free from isomeric impurities (Scheme 3) [40]. In the mid-1990’s, the ‘3 + 1’ route was applied to the synthesis of porphyrins and *b*-annulated porphyrins [36,41,42,43,44,45,46,47]. Using trifluoroacetic acid as a catalyst, isomerically pure porphyrin products were generally obtained [46], although exceptions have been noted [48]. In addition, two groups independently utilized this approach to prepare carbaporphyrinoid systems. Berlin and Breitmaier prepared a benziporphyrin **17** by reacting isophthalaldehyde with tripyrrane **13** in the presence of HBr in acetic acid [49], while Lash reported the synthesis of oxybenziporphyrin **18** [19] by condensing 4-formylsalicylaldehyde with **13** in the presence of TFA, followed by oxidation with 2,3-dichloro-5,6-dicyano-1,4-benzoquinone (DDQ) (Scheme 3). Benziporphyrin **17** was mistakenly reported to have multiple tautomeric forms that failed to interconvert on the NMR timescale [49], but the results were subsequently shown to be due to the presence of isomeric impurities [15,50,51]. This problem persisted in early attempts to prepare carbaporphyrins using the HBr-catalyzed conditions [52,53]. However, pure carbaporphyrins were obtained when TFA was used as a catalyst, and these conditions have allowed the synthesis of structurally diverse carbaporphyrinoid structures (Scheme 4) [15,19,40,50,54,55,56,57,58,59,60,61,62,63,64,65,66,67,68,69,70,71,72,73,74,75]. Tripyrrane analogues have also been used to prepare porphyrin analogues, including heterotripyrranes [76,77], azulitripyrranes [61,78,79,80], benzitripyrranes [81,82,83,84], and pyritripyrranes [85] (Scheme 5). Although less commonly used, MacDonald ‘2 + 2’ syntheses of carbaporphyrinoids have been reported, including *meso*-unsubstituted NCP **19** [86], azuliporphyrin **20** [87], and neo-confused porphyrins such as **21** and **22** [88,89,90] (Scheme 6).

**Scheme 7:**
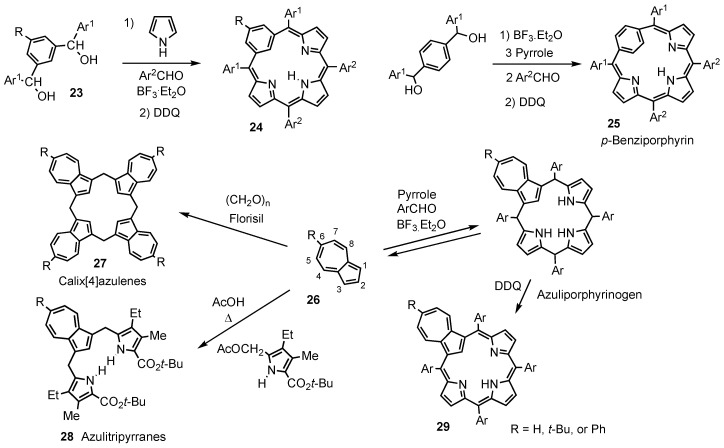
Direct syntheses of *meso*-tetraaryl carbaporphyrinoids.

One pot syntheses of *meso*-tetraarylbenziporphyrins [91,92] and azuliporphyrins [93,94,95] have also been reported (Scheme 7). Benzenedicarbinols **23** react with pyrrole and aromatic aldehydes in the presence of BF_3_.Et_2_O to give, following an oxidation step, benziporphyrins **24** [91]. This approach was also used to prepare *p*-benziporphyrins **25** from 1,4-benzenedicarbinols, albeit in low yield [96]. Azulene **26** favors electrophilic substitution at the 1,3-positions, which are analogous to the α-positions of pyrroles, and this characteristic has been utilized in the preparation of calix[4]azulenes **27** [97,98], azulitripyrranes **28** [78], and *meso*-tetraarylazuliporphyrins **29** [93,94,95] (Scheme 7). Reaction of azulene or 6-substituted azulenes, with three equivalents of pyrrole and four equivalents of an aryl aldehyde in the presence of BF_3_.Et_2_O in chloroform, followed by oxidation with DDQ, gave azuliporphyrins **29** in up to 20% yield [93,94,95]. Given that the reaction requires the selective formation of eight covalent bonds between a 1:3:4 mixture of three different reagents, the outcome of the chemistry is remarkable.

**Scheme 8:**
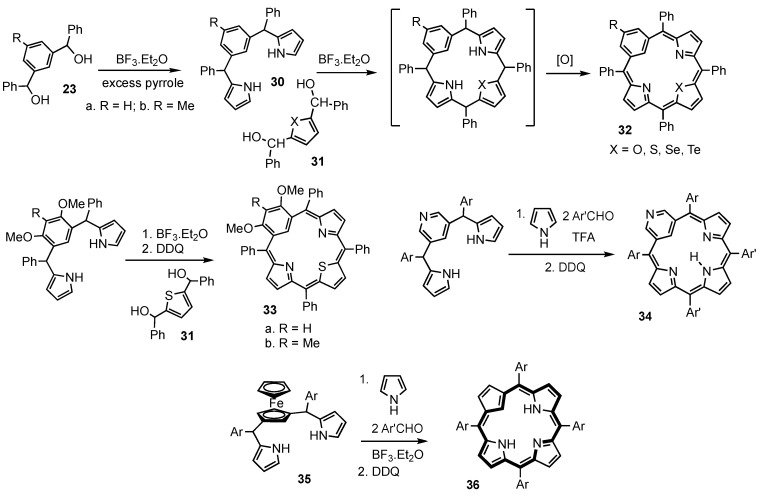
Stepwise syntheses of *meso*-tetraaryl carbaporphyrinoids.

Stepwise routes to *meso*-substituted carbaporphyrinoids are also known (Scheme 8). For example, benzenedicarbinols **23** react with excess pyrrole and BF_3_.Et_2_O to afford benzitripyrranes **30** and these condense with heterocyclic dicarbinols **31** to afford a series of heterobenziporphyrins **32**^83^ Dimethoxythiabenziporphyrins **33**^84^ and inverted pyriporphyrins **34** [85,99], where a pyridine subunit has been incorporated with the nitrogen orientated towards the periphery of the macrocycle (N-confused pyriporphyrins), were prepared similarly. In an innovative application of this strategy, a ferrocene-embedded tripyrrane analogue **35** was used to generate tetraphenylcarbaporphyrin **36** [100]. The ferrocene unit acts as a protected cyclopentadienyl moiety and spontaneously demetalates to give the macrocyclic product. Tripyrrane analogues have been widely applied to the synthesis of expanded porphyrinoid systems [101].

**Scheme 9:**
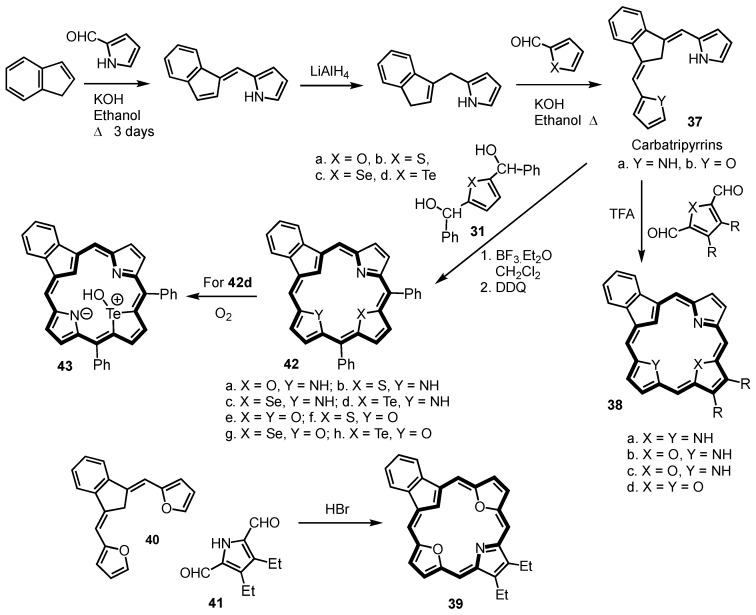
Synthesis of carbaporphyrins and heteroanalogues from carbatripyrrins.

An alternative route to carbaporphyrins and their heteroanalogues from carbatripyrrins has been developed (Scheme 9) [102]. Carbatripyrrin **37a** and oxacarbatripyrrin **37b** can be prepared in three steps from technical grade indene. Condensation with pyrrole and furan dialdehydes gave moderate yields of macrocyclic products **38** [102,103] and related carbaporphyrins with fused phenanthrene [102], acenaphthylene, pyrene and chrysene units [104] were also obtained. In addition, dioxacarbaporphyrin **39** was generated when dioxocarbatripyrrin **40** was reacted with pyrroledicarbaldehyde **41** [105]. Unfortunately, many of carbaporphyrins prepared by this strategy are poorly soluble due to the absence of substituents. This problem can be overcome by reacting **37a** and **37b** with furan, thiophene, selenophene and tellurophene dicarbinols **31a**–**d** in the presence of boron trifluoride etherate, followed by oxidation with DDQ, to give a series of relatively soluble diphenyl-heterocarbaporphyrins **42a**–**h**, including the first examples of porphyrin analogues with four different elements within the macrocyclic core [102,103,106]. Telluracarbaporphyrin **42d** proved to be prone to air oxidation and afforded the hydroxytellurophene derivative **43**.

**Scheme 10:**
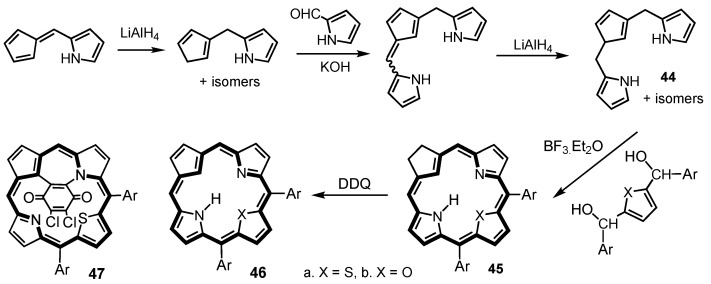
Synthesis of heterocarbaporphyrins from a cyclopentadiene tripyrrane analogue.

A cyclopentadiene analogue of the tripyrranes **44** similarly reacted with a thiophene dicarbinol to give low yields of heterocarbachlorin **45a** (Scheme 10) [107,108]. Oxidation with DDQ afforded the related thiacarbaporphyrin **46a** in 25% yield together with the quinone addition product **47**. Very recently, related oxacarbachlorins **45b** and oxacarbaporphyrins **46b** were prepared in a similar fashion [109].

**Scheme 11:**
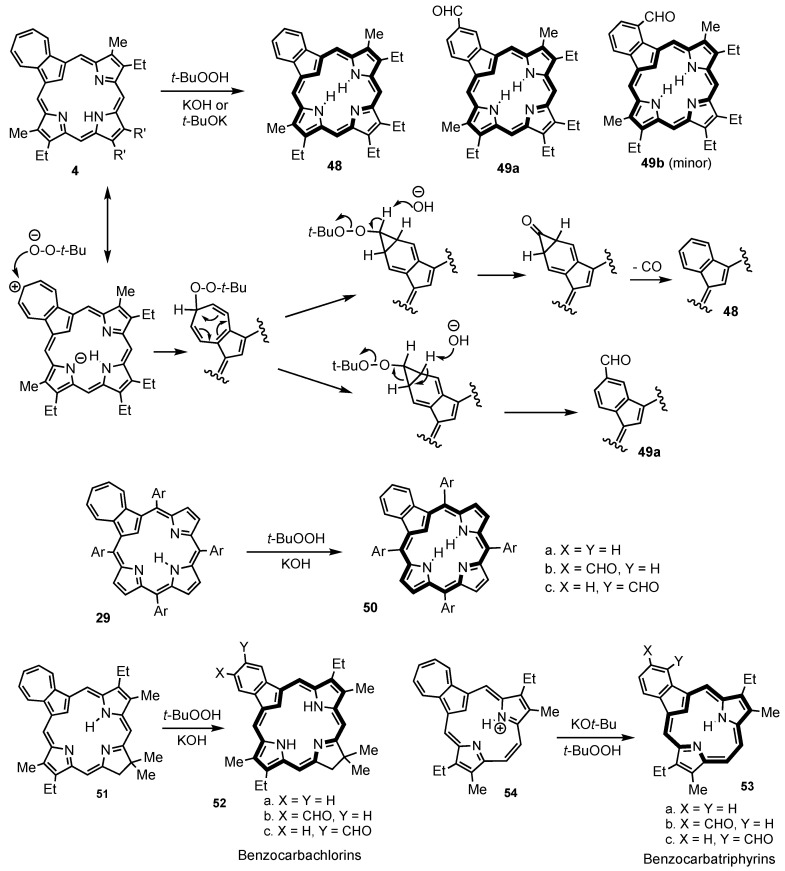
Oxidative ring contraction of azuliporphyrinoids.

Finally, it may also be possible to convert specific carbaporphyrin-like systems into other classes of carbaporphyrinoids. The best-known strategy of this type involves oxidative ring contraction of azuliporphyrins **4** to give benzocarbaporphyrins (Scheme 11) [110]. Reaction of azuliporphyrin **4** with *tert*-butyl hydroperoxide in the presence of base initiates nuclophilic attack from a peroxide anion, and subsequent Cope rearrangement and elimination affords mixtures of benzocarbaporphyrins **48** and related aldehydes **49a,b** [61]. Tetrarylazuliporphyrins **29** similarly give the related *meso*-substituted benzocarbaporphyrins **50** [93,94,95]. The same strategy has been applied to the synthesis of carbachlorins **51** from azulichlorins **52** [111] and carbatriphyrin(1.2.1)s **53** from azulitriphyrins **54** [112]. Ring contraction reactions triggered by metalation are discussed in later sections of this review.

## 3. Organometallic Chemistry of N-Confused Porphyrins

**Figure 2:**
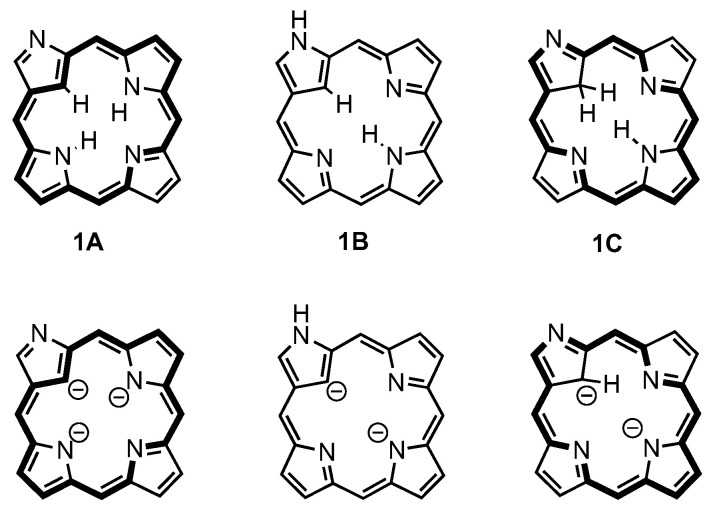
Three tautomers of N-confused porphyrin and formal representations of the corresponding di- and trianionic ligands.

**Scheme 12:**
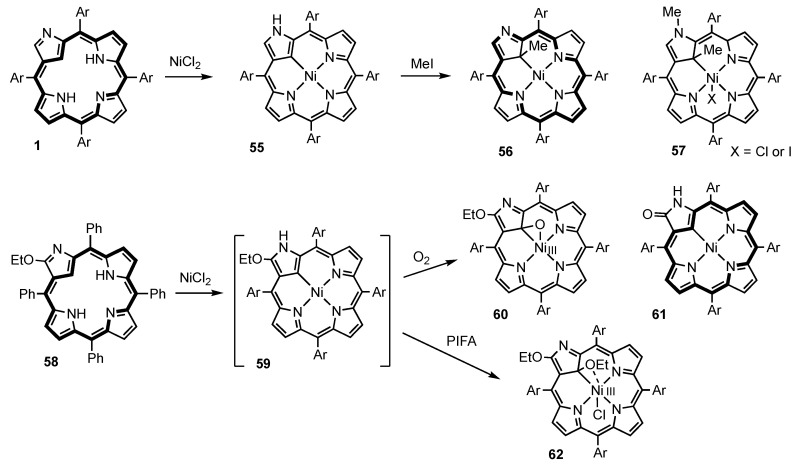
Synthesis of nickel(II) and nickel(III) NCPs.

N-Confused porphyrins are particularly proficient at forming organometallic derivatives and can act as dianionic or trianionic ligands (Figure 2). As the confused nitrogen, i.e., the external nitrogen atom, bears a proton in tautomer **1B**, replacement of the two internal protons facilitates the incorporation of transition metal dications. However, replacement of all three protons in tautomer **1A** would provide a suitable environment for trications. Although tautomer **1A** is slightly more stable than **1B** and is favored in nonpolar solvents, **1B** is sufficiently close in energy to be accessible and in fact it is the primary species in polar aprotic solvents such as DMF and DMSO [113]. The earliest report on the formation of an organometallic derivative for NCP **1** (Ar = *p*-tolyl) involved reaction with nickel(II) chloride to give a nickel(II) complex **55** in which one of the NH protons had been relocated onto the external nitrogen (Scheme 12) [32]. As is the case for tautomer **1B**, this structure is cross-conjugated and exhibits greatly reduced aromatic characteristics. Reaction of **55** with methyl iodide gave the C-methylated nickel(II) complex **56** together with a dialkylated nickel(III) species **57** (Scheme 12) [114]. Complex **56** can be considered to be derived from an dianionic ligand corresponding to tautomer **1c** (Figure 2). Metal complexes formally derived from tautomer 1B have been reported for Pd(II) [115], Pt(II) [116,117,118], Cu(II) [119,120], Mn(III) [121], Co(II) [122], Rh(IV) [123,124] and Mo(II) [125]. Reaction of 3-ethoxy-NCP **58** with NiCl_2_ gave unstable nickel(II) complex **59** and this gradually air oxidized to give oxygen bridged nickel(III) complex **60** together with nickel(II) carbaporpholactam **61** [126]. When intermediate **59** was oxidized with (bis(trifluoroacetoxy)iodobenzene (PIFA) in chloroform-ethanol, nickel(III) 3,21-diethoxyNCP **62** was generated.

**Scheme 13:**
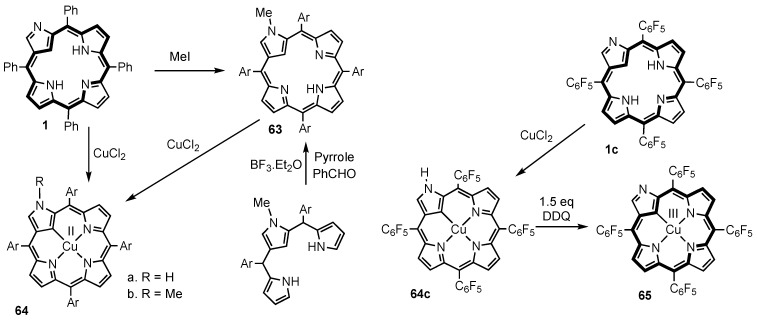
Copper complexes of N-confused porphyrins.

Copper(II) and copper(III) complexes of NCP have been reported (Scheme 13) [119,120]. Reaction of NCP **1** or 21-methylNCP **63** with copper(II) acetate afforded copper(II) complexes **64a** and **64b**, respectively. Tetrakis(pentafluorophenyl) NCP **1c** also generated copper(II) complex **64c** but this was readily converted into the corresponding copper(III) complex **65** upon treatment with 1.5 equivalents of DDQ [120]. However, this species is somewhat unstable and solutions in CHCl_3_ or CH_2_Cl_2_, **65** gradually converted back into **64c**.

**Scheme 14:**
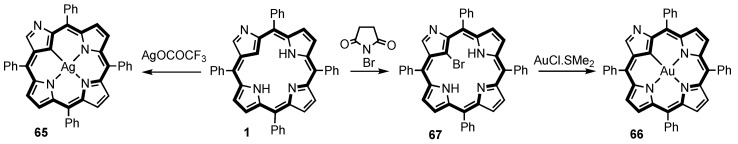
Synthesis of silver(III) and gold(III) NCPs.

**Scheme 15:**
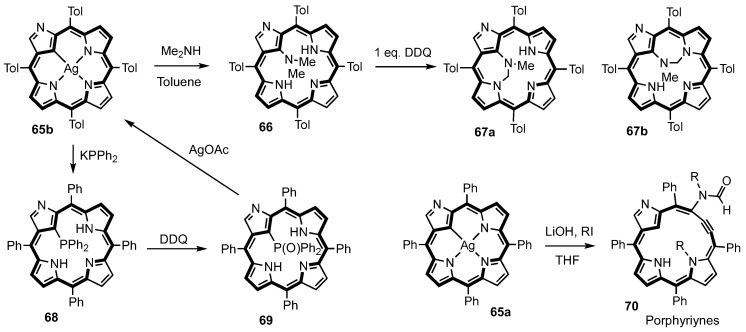
Synthesis of new porphyrinoids from silver(III) NCPs.

Tetraphenyl NCP 1a reacted with silver(I) trifluoroacetate to give silver(III) complex **65** (Scheme 14) [127]. NCP acts as a trianionic ligand in this case and the silver(I) cation is transformed into the Ag(III) complex. This is believed to occur via a disproportionation reaction: 3 Ag^+^ → 2 Ag^3+^ + 2 Ag° [128]. The macrocycle retains a porphyrin-like 18π electron delocalization pathway and **65** exhibits strongly diatropic properties. The related gold(III) complex **66** cannot be obtained directly from **1**, but instead it is necessary to initially carry out a monobromination with *N*-bromosuccinimide to form 21-bromoNCP **67** and subsequent reaction with 3.3 equiv of AuCl.SMe_2_ then gives **66** [129]. Gold(III) complex **66** exhibited unique luminescent properties at room temperature. Silver(III) NCPs have modified reactivity that enables unusual structural transformations to occur (Scheme 15). For instance, reaction of **65a** with dimethylamine results in oxidative addition to afford 21-dimethyl NCP **66** [130]. Oxidation with one equivalent of DDQ produced internally bridged NCPs **67a** and **67b**. Reaction of **1a** with potassium diphenylphosphide afforded 21-diphenylphosphanyl-NCP **68** (Scheme 15) [131]. Oxidation of **68** with DDQ generated the related diphenylphosphoryl-NCP **69** but attempts to metalate this compound with silver(I) acetate led to elimination of the phosphoryl unit and conversion back to silver(III) NCP **65a**. Thiophosphorylation was also observed when **68** was reacted with elemental sulfur (S_8_)^131^. Another intriguing transformation occurs when silver(III) NCP **65a** is treated with lithium hydroxide and methyl or ethyl iodide [132]. Under these conditions, cleavage of the confused ring, together with demetalation, occurs to produce a novel ethynyl-linked triphyrin **70**. This system, named porphyriyne, has a porphyrin-like UV-vis spectrum with a Soret band at 418 nm, and the proton NMR spectrum demonstrates the presence of a strong diamagnetic ring current [132].

**Figure 3:**
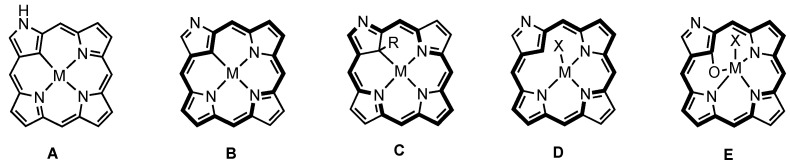
Coordination modes exhibited by N-confused porphyrins.

**Scheme 16:**
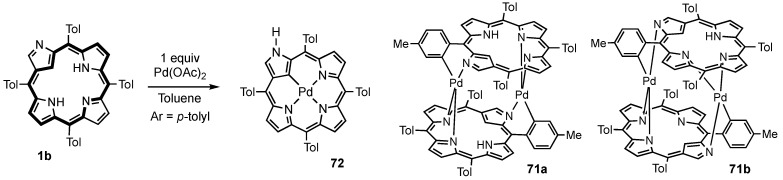
Palladium complexes of N-confused porphyrins.

As noted above, NCPs can act as dianionic or trianionic ligands and afford metal complexes **A**–**C** (Figure 3) formally related to tautomers **1A**–**C** (Figure 2), respectively. NCPs give rise to diverse coordination complexes. In addition to silver(III) and gold(III) complexes, type **B** complexes include Co(III) [122], Rh(III) [123,124], and Sb(V) [133] derivatives. Many examples of type **D** complexes, which lack direct carbon-metal bonds, have been reported including zinc [134], manganese(II) and iron(II) derivatives [135,136,137]. The manganese(II) and iron(II) derivatives react with molecular oxygen to produce internally oxo-bridged complexes of type **E**. NCPs can also coordinate at the external nitrogen and this facilitates the formation of diverse structures. For instance when NCP **1b** was reacted with palladium(II) acetate in refluxing toluene, aryl-bridged dimers **71a** and **71b** were formed, together with the organometallic palladium(II) complex **72** (Scheme 16) [115]. The remarkable coordination chemistry of NCPs has been widely reviewed elsewhere and more detailed descriptions fall outside of the scope of the current review.

**Scheme 17:**
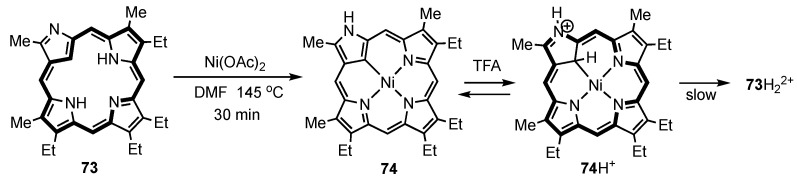
Protonation of a nickel(II) N-confused porphyrin complex.

**Scheme 18:**
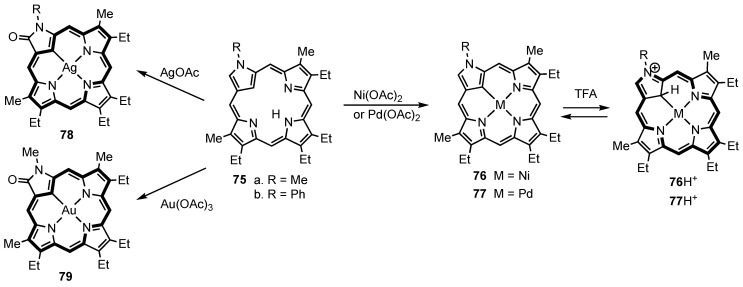
Metalloporphyrinoids derived from 2-methyl and 2-phenyl NCPs.

Although less well studied, metalated derivatives of *meso*-unsubstituted NCPs have also been investigated [56,62]. Much of the initial work on related carbaporphyrinoid systems was carried out on *meso*-unsubstituted structures and for this reason complexes of *meso*-unsubstituted NCPs allow valuable comparisons to be made. NCP **73** reacted with nickel(II) acetate in DMF at 145 °C to give the corresponding nickel(II) complex **74** (Scheme 17) [56]. This complex exhibited greatly reduced aromatic character, but when TFA was added to a solution of **74** in CDCl_3_ a strong diamagnetic ring current was generated. An internal CH resonance was observed at −4.93 ppm, while the external *meso*-protons gave rise to four singlets between 9.37 and 10.01 ppm. The new species was identified as a C-protonated nickel complex **74** with an 18π electron delocalization pathway. Protonation is reversible, but cation **74** slowly demetalated in the presence of TFA to give protonated **73**. Similar C-protonation was subsequently reported for copper(II) and nickel(II) complexes of tetraphenyl-NCP [119]. *N*-Methyl and *N*-phenyl NCPs **75** also gave nickel(II) and palladium(II) complexes, **76** and **77**, respectively, and addition of TFA to solutions of these organometallic derivatives similarly afforded aromatic C-protonated species **76**H^+^ and **77**H^+^, respectively (Scheme 18) [62]. Higher concentrations of TFA were required to protonate palladium complexes **77** compared to the **76**, but the resulting palladium cations **77**H^+^ proved to be far more stable under acidic conditions [62]. NCPs **75** reacted with silver(I) acetate to give silver(III) carbaporpholactams **78** and these proved to be highly diatropic compounds (Scheme 18). The proton NMR spectrum for a solution of **78a** in CDCl_3_ showed the *meso*-protons as four downfield 1H singlets between 9.10 and 9.86 ppm. NCP **75a** also reacted with gold(III) acetate to give a low yield of the related gold(III) complex **79** [62].

## 4. X-Confused Heteroporphyrins

**Figure 4:**
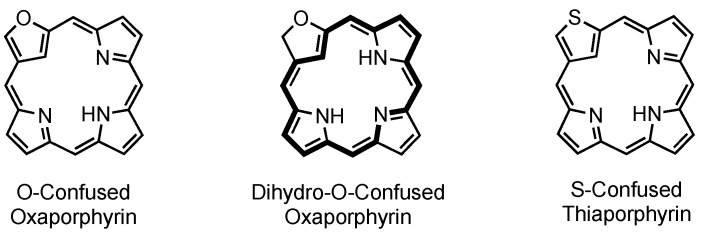
X-Confused Heteroporphyrins.

**Scheme 19:**
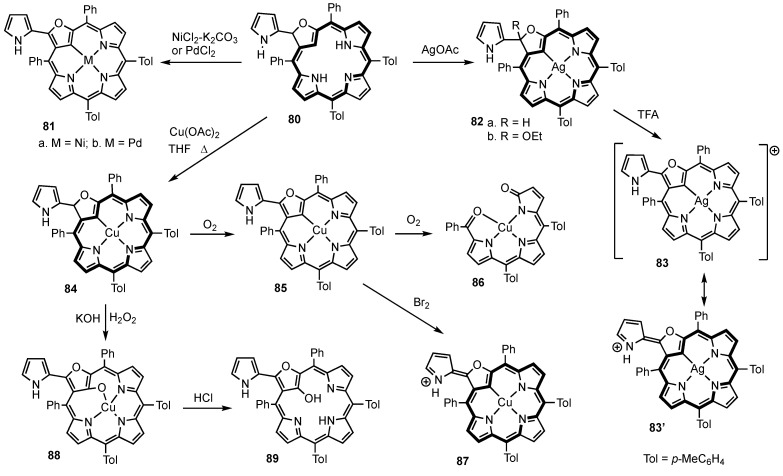
Metal complexes of pyrrole-appended O-confused porphyrinoids.

O-confused oxaporphyrins and S-confused thiaporphyrins, collectively known as X-confused heteroporphyrins, have similar structures to NCPs but possess inverted furan or thiophene units in place of the confused pyrrole moiety (Figure 4) [138]. Although X-confused heteroporphyrins are cross-conjugated and only weakly aromatic, dihydro-O-confused oxaporphyrins are chlorin analogues that possess macrocyclic aromaticity due to the presence of 18π electron delocalization pathways. Pyrrole-appended O-confused porphyrinoid **80** reacted with nickel(II) chloride or palladium(II) chloride in the presence of anhydrous potassium carbonate to afford the corresponding metal complexes **81a** and **81b**, respectively (Scheme 19) [139]. O-Confused porphyrinoid **80** exhibits macrocyclic aromaticity and proton NMR spectroscopy shows that it possesses a strong diatropic ring current. However, nickel(II) and palladium(II) complexes **81** have substantially reduced diatropicity due to the furan unit introducing a cross-conjugated element. When **80** was reacted with silver(I) acetate in acetonitrile, a fully aromatic silver(III) complex **82a** was formed but when ethanol was added to the reaction mixture, the related ethoxy-derivative **82b** was generated [139]. Addition of TFA facilitated elimination of ethanol to give the highly diatropic cation **83**. The aromatic properties exhibited by this species can be attributed to electron-donation from the pyrrole substituent (resonance structure **83′**). Porphyrinoid **80** also reacted copper(II) acetate in refluxing THF to give copper(III) complex **84** in quantitative yield (Scheme 19) [140]. This organometallic complex also gave a proton NMR spectrum that was consistent with an aromatic macrocycle, although the downfield shifts to the external protons were reduced compared to silver(III) complex **82a**. In the presence of oxygen, **84** was initially converted into copper(II) complex **85** but further exposure to O_2_ resulted in oxidative cleavage to give tripyrrinone complex **86** (Scheme 19). Addition of bromine to **85** generated an aromatic cation **87** that was analogous to silver(III) cation **83**. When copper(III) complex 84 was treated with hydrogen peroxide in the presence of KOH, an oxygen atom was inserted into the macrocyclic core to give **88**, and this could be demetalated with hydrochloric acid to afford hydroxyporphyrinoid **89**.

**Scheme 20:**
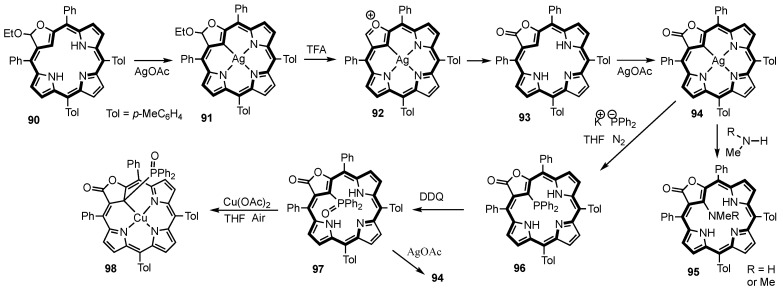
Synthesis and reactivity of Ag(III) and Cu(II) O-confused porphyrinoids.

Ethoxy-O-confused porphyrinoid **90** reacted with silver(I) acetate to give the silver(III) organometallic complex **91**, and subsequent addition of TFA led to elimination of ethanol to afford cationic complex **92** (Scheme 20) [141]. This species was unstable and slowly converted into the carbaporpholactone **93**, most likely due to nucleophilic attack from water followed by oxidation. As silver(I) is lost during this process, silver(III) complex **93** may be responsible for the observed oxidation. Porphyrinoid lactone **93** could be remetalated with silver(I) acetate to give the silver(III) complex **94**. Reaction of **94** with methylamine or dimethylamine resulted in demetalation and the formation of amino-derivatives **95** [130], while treatment with sodium diphenylphosphide afforded the phosphine derivative **96** [142]. This derivative was oxidized with DDQ to produce the corresponding phosphonate **97**, and further reaction with copper(II) acetate in the presence of air afforded a nonaromatic copper(II) complex **98**. However, treatment of **97** with silver(I) acetate led to loss of the phosphonyl group and regeneration of the silver(III) complex **94** [142].

**Scheme 21:**
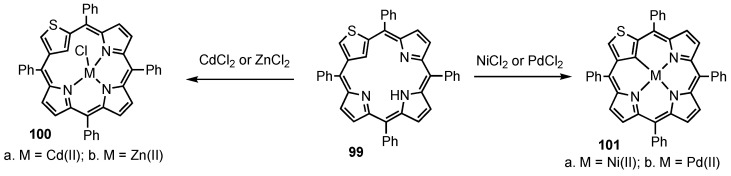
Metal complexes of S-confused thiaporphyrins.

S-Confused thiaporphyrin **99** acts as a monoanionic ligand when reacted with cadmium(II) chloride in chloroform or zinc(II) chloride in THF to give coordination complexes **100a** and **100b** possessing axial chlorides (Scheme 21) [143]. Coupling between the protons and carbon-13 nuclei of the thiophene unit and the NMR active cadmium isotopes (^111^Cd and ^113^Cd) suggest that there is a strong agostic interaction between the metal and thiophene units despite the absence of a formal carbon-metal bond. This interpretation is supported by the X-ray crystallographic data. Organometallic nickel(II) and palladium(II) complexes **101** were also obtained from **99**, demonstrating that S-confused thioporphyrins can also act as dianionic ligands [25].

## 5. Organometallic Chemistry of True Carbaporphyrins

**Figure 5:**
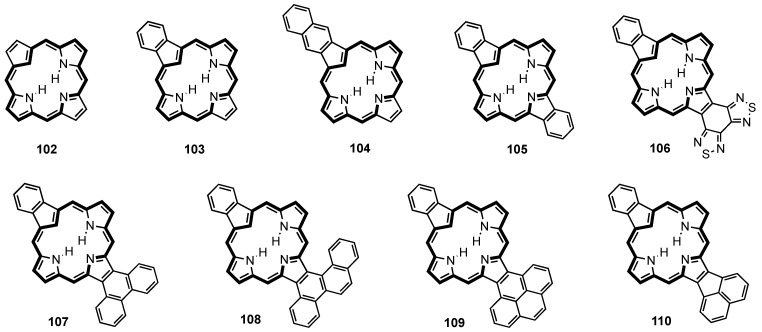
Selected Examples of True Carbaporphyrins.

**Scheme 22:**
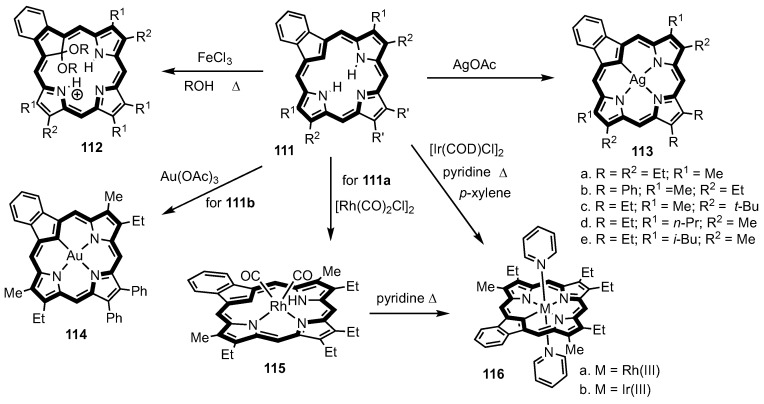
Metalation of *meso*-unsubstituted carbaporphyrins.

Carbaporphyrins retain the porphyrin framework but replace one of the nitrogens with a carbon atom. In early studies, the term “true carbaporphyrins” was introduced [22,144] to differentiate structures such as **102**–**110** (Figure 5) [15,58,67,71,72,102,104] from other carbaporphyrinoid systems including N-confused porphyrins and azuliporphyrins. This definition includes ring fused structures such as **103**–**110** in much the same way as benzoporphyrin would be considered to be a “true porphyrin” [145,146]. Much later, another author considered only **102** to be a true carbaporphyrin [108] but given that the original definition predates this by at least a dozen years, we suggest that our definition is more appropriate. In any case, the reactivity, aromaticity, and spectroscopic properties of carbaporphyrins are no more affected by ring fusion of this type than they are by introducing electron-withdrawing substituents [69]. Early investigations into the metalation of carbaporphyrins were performed on benzocarbaporphyrins **111** (Scheme 22) due in part to the relative accessibility of these porphyrinoids. Initially, attempts were made to react first row transition metal cations, including Ni(II), Cu(II) and Co(II), were unsuccessful, although **111** was shown to undergo a regioselective oxidation in the presence of 500 equivalents of iron(III) chloride in alcohol solvents to give ketal derivatives **112** (Scheme 22) [147,148]. Ketals **112** gave intense long wavelength absorptions and proved to be effective agents in the treatment of leishmaniasis [149,150]. Subsequently, reaction of benzocarbaporphyrins at room temperature with silver(I) acetate generated silver(III) complexes **113** in excellent yields [144,151]. Silver(III) carbaporphyrin complexes retain highly diatropic characteristics and the proton NMR spectra for **112a** showed the resonances for the *meso*-protons downfield near 10 ppm. The UV-vis spectra for these stable organometallic derivatives were also porphyrin-like and gave a strong Soret band at 437 nm [144,151]. The X-ray crystal structure for benzocarbaporphyrin **111b** showed that the indene unit was tilted by approximately 15° relative to the mean macrocyclic plane, but when the silver(III) cation replaces the three inner hydrogens complex **112b** takes on a near planar conformation. *meso*-Unsubstututed benzocarbaporphyrin **111b** also reacted with gold(III) acetate to give low yields of the corresponding gold(III) complex **113** (Scheme 22) [151]. Reaction of **111a** with [Rh(CO)_2_Cl]_2_ generated rhodium(I) complex **114** and this underwent oxidation in refluxing pyridine to afford rhodium(III) carbaporphyrin complex **115a** [152]. Prior to this study, rhodium(III) N-confused porphyrins had been prepared in the same way [124]. When **111a** was heated with [Ir(COD)Cl]_2_, a closely related iridium(III) complex **115b** was generated [152]. Interestingly, iridium(III) complexes of NCPs are not currently known.

**Scheme 23:**
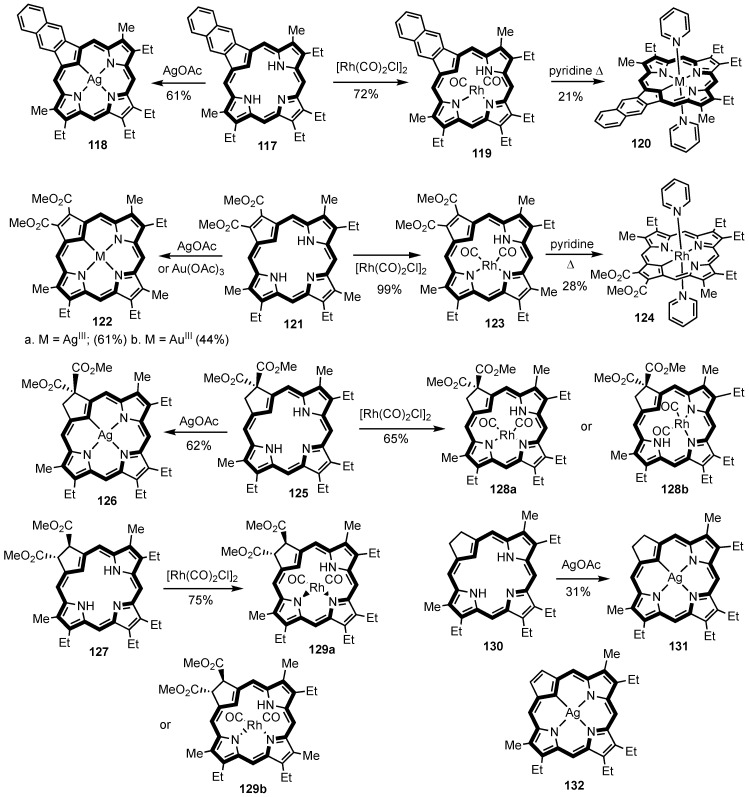
Metalation of carbaporphyrins and carbachlorins.

**Scheme 24:**
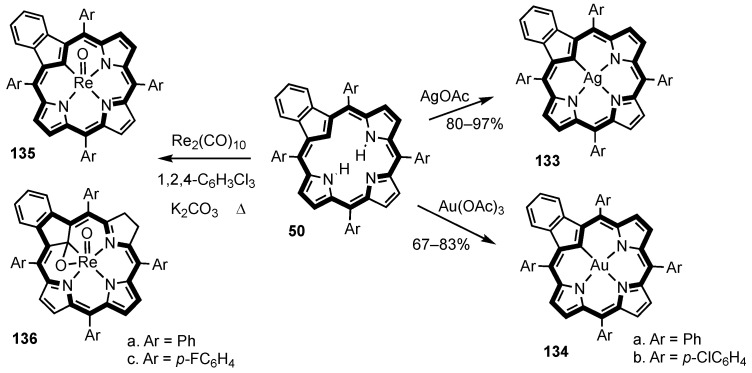
Metalation of tetraarylcarbaporphyrins.

Related carbaporphyrins undergo similar metalation reactions (Scheme 23) [153]. Naphthocarbaporphyrin **117** reacted with silver(I) acetate to give **118** [71], while treatment with [Rh(CO)_2_Cl]_2_ afforded rhodium(I) complex **119** [154]. As seen for the benzocarbaporphyrin series, heating **119** with pyridine afforded the corresponding rhodium(III) complex **120**. Carbaporphyrin diester **121** reacted with AgOAc to produce silver(III) complex **122a** [69], while metalation with Au(OAc)_3_ afforded an excellent yield of gold(III) complex **122b** [69]. The electron-withdrawing ester moieties appear to stabilize the macrocycle and this inhibits oxidation reactions. Reaction of **121** with [Rh(CO)_2_Cl]_2_ gave rhodium(I) complex **123** and this was converted into the related rhodium(III) derivative **124** in refluxing pyridine [152]. Carbachlorin **125**, which is protected from oxidation on the carbocyclic ring by the presence of a *gem*-diester unit, formed silver(III) complex **126** with AgOAc [69]. Carbachlorins **125** and **127** also gave rhodium(I) complexes **128** and **129**, respectively, with [Rh(CO)_2_Cl]_2_ [154]. Although the NMR spectra showed that single products had been formed, in both cases two different structures **128a,b** and **129a,b** were consistent with the available data (Scheme 24). X-ray crystal structures could not be obtained, and it was not possible to determine which specific isomers had been formed. Finally, carbachlorin **130** was shown to react with 3.5 equiv of AgOAc to give silver(III) carbachlorin **131**. However, the reaction occurred relatively slowly compared to carbaporphyrins [67]. When a larger excess of AgOAc was used, a low yield of impure carbaporphyrin complex **132** was isolated.

*meso*-Tetraarylbenzocarbaporphyrins **50** also reacted with silver(I) acetate to give the silver(III) complexes **133** (Scheme 24) [151]. Reaction with gold(III) acetate in refluxing pyridine gave much better results for this series, affording gold(III) derivatives **134** in 67–83% yield [151]. The presence of *meso*-substituents appears to protect the macrocycle from oxidative degradation. Reaction of benzocarbaporphyrins **50** with Re_2_(CO)_10_ and potassium carbonate in refluxing 1,2,4-trichlorobenzene gave oxorhenium(V) complex **135** and oxygen-bridged rhenium(VII) complex **136** [155]. The structures of these complexes were confirmed by X-ray crystallography. The formation of such unusual derivatives indicates that benzocarbaporphyrins may prove to have untapped potential in the formation of organometallic complexes.

**Scheme 25:**
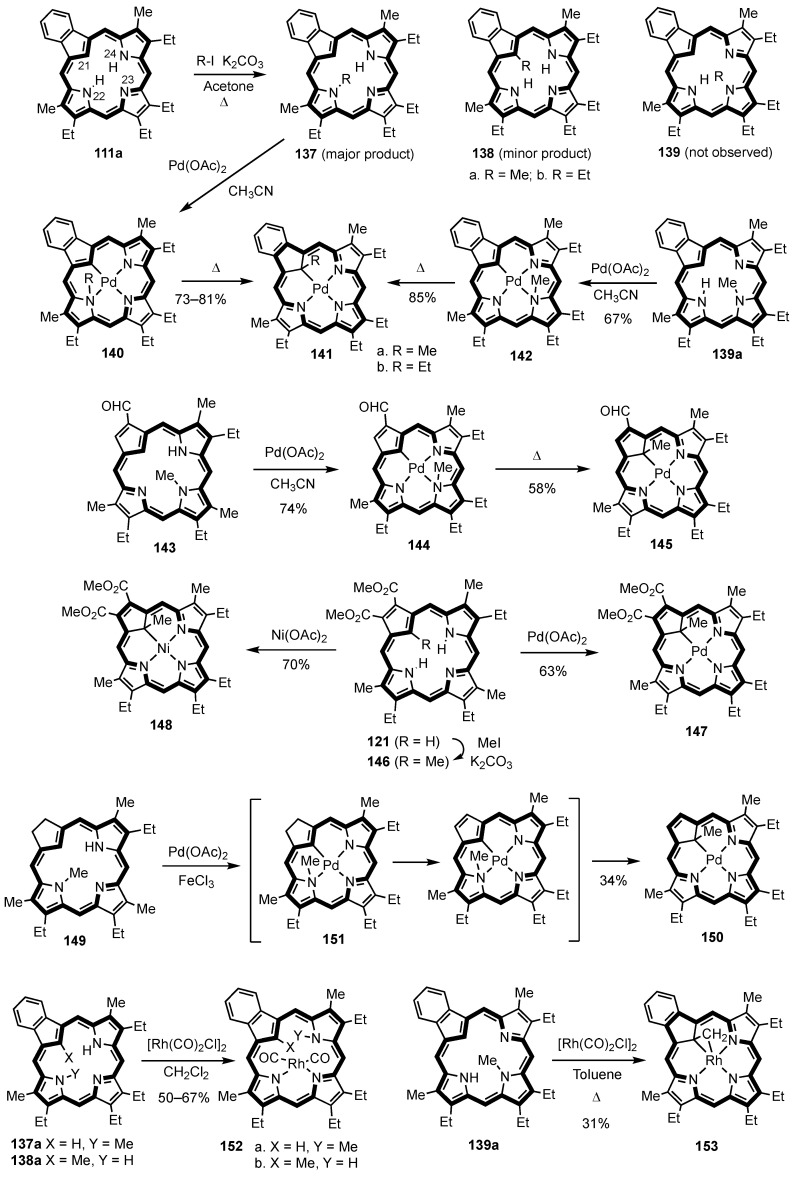
Pd(II), Ni(II) and Rh complexes of internally alkylated carbaporphyrins.

Carbaporphyrins generally act as trianionic ligands. In an attempt to convert this system into a dianionic ligand, the introduction of *N*-alkyl substituents was investigated [156]. Reaction of benzocarbaporphyrin **111a** with methyl or ethyl iodide in the presence of potassium carbonate gave a mixture on *N*- and C-substituted benzocarbaporphyrins **137** and **138**, respectively (Scheme 25) [156]. The major products **137** were alkylated at position 22; no trace of alkylation at the 23-position to give **139** was observed. *N*-Alkyl carbaporphyrins **137** were heated with palladium(II) acetate in acetonitrile with the expectation that palladium(II) complexes **140** would be generated (Scheme 25). However, the metalation reaction occurred with concomitant alkyl group migration to give C-alkyl palladium(II) complexes **141**. When the reaction was stopped after a few minutes, complexes **140a,b** were observed but attempts to purify these derivatives by column chromatography were unsuccessful as partial conversion to **141** always took place. It was suggested that alkyl group migration could involve a [1,5] sigmatropic rearrangement [156], but a stepwise mechanism involving a transient Pd-alkyl species is now favored [157]. Palladium(II) complexes **141** retain strongly diatropic characteristics. In the proton NMR spectrum for **141a**, the *meso*-protons gave two downfield 2H singlets at 9.56 and 10.27 ppm, while the internal methyl group afforded an upfield 3H resonance at −3.21 ppm [156]. In order to further examine this chemistry, 23-methylbenzocarbaporphyrin **139a** was prepared from an *N*-methyltripyrrane [157]. Reaction of **139a** with Pd(OAc)_2_ in refluxing acetonitrile gave an *N*-methyl complex **142** that could be isolated and fully characterized. When the reaction mixture was heated under reflux for 16 h, the rearranged C-methyl derivative **141a** was generated. *N*-Methyl-carbaporphyrin aldehyde **143** similarly reacted with Pd(OAc)_2_ to give palladium(II) *N*-methyl complex **144** [40]. This compound could be isolated and characterized, but longer reaction times gave C-methyl derivative **145**. Alkylation of carbaporphyrin diester **121** with methyl iodide and potassium carbonate in refluxing acetone afforded 21-methylcarbaporphyrin **146** [69]. Reaction with Pd(OAc)_2_ generated palladium(II) complex **147**, while treatment with Ni(OAc)_2_ gave nickel(II) complex **148** [69]. *N*-Methyl carbachlorin **149** was also reacted with Pd(OAc)_2_ and gave a low yield of the rearranged palladium(II) complex **150** [67]. It was proposed that this conversion involves a sequential metalation-oxidation-rearrangement process and yields were substantially improved when the oxidant FeCl_3_ was present. Attempts to isolate the intermediary carbachlorin complex **151** were unsuccessful. Reactions of 22-, 21- and 23-methylcarbaporphyrins **137a**, **138a** and **139a** with di-μ-chlorotetracarbonyldirhodium(I) were also investigated [157]. Conventional rhodium(I) complexes **152** were obtained for **137a** and **138a**, but the presence of a 23-methyl group in **139a** blocks the formation of this type of structure. However, when **139a** was heated with [Rh(CO)_2_Cl]_2_ in toluene, an usual rhodium(III) complex **153** was isolated in 31% yield. The identity of this structure was confirmed by X-ray crystallography. In this case, the methyl group has again migrated onto the internal carbon atom but is converted into a bridging methylene unit. Hence, alkyl group migration is not limited to palladium(II) carbaporphyrins. In the proton NMR spectrum for **153**, the methylene bridge gave rise to a broadened doublet at −3.22 ppm while the *meso*-protons appeared downfield as two 2H singlets at 9.51 and 10.13 ppm. These data demonstrate that the macrocycle retains strongly aromatic properties and also shows that ^103^Rh (100% natural abundance, *I* = ½) is coupling to the methylene unit.

**Scheme 26:**
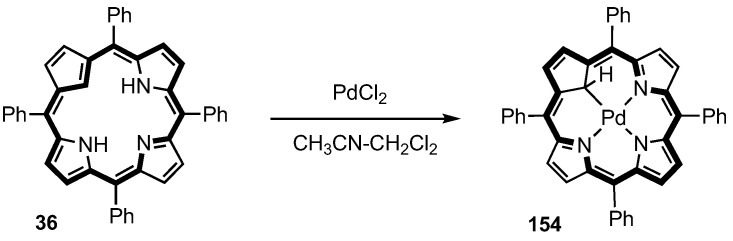
Palladium(II) complex of tetraphenylcarbaporphyrin.

Directly reacting benzocarbaporphyrins **111** with palladium(II) acetate or palladium(II) chloride primarily led to decomposition. However, tetraphenylcarbaporphyrin **36** has been shown to react with PdCl_2_ to generate palladium complex **154** (Scheme 26) [100,158].

**Figure 6:**
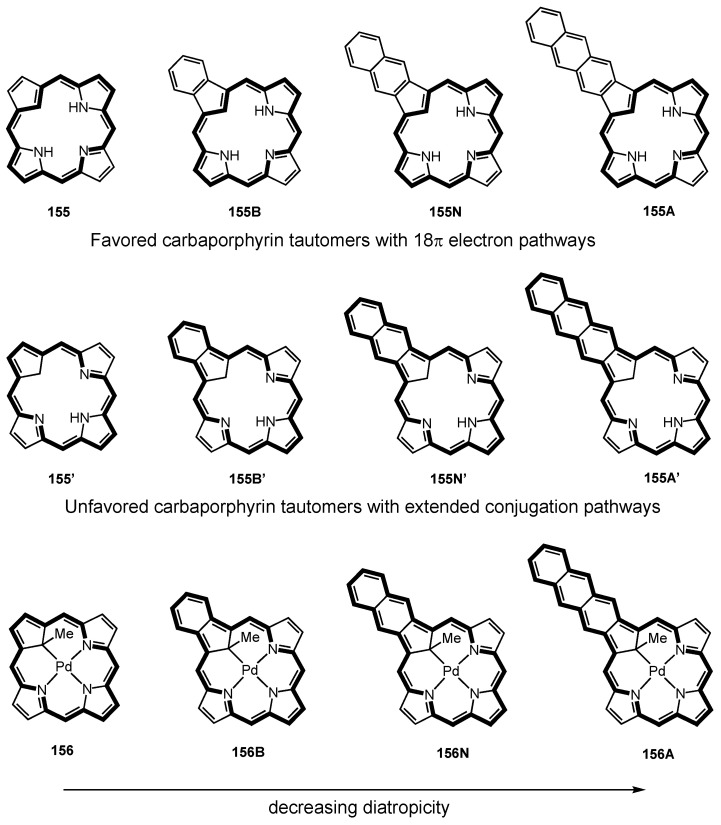
Carbaporphyrin tautomers and extended aromatic conjugation pathways in palladium(II) complexes.

**Scheme 27:**
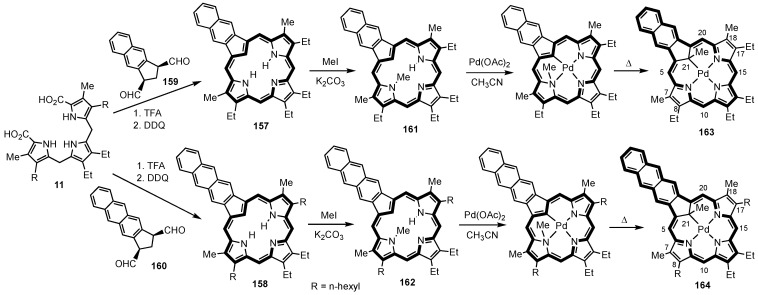
Palladium(II) complexes of naphtho- and anthrocarbaporphyrins.

Carbaporphyrins favor tautomers such as **155** and **155B** that have three hydrogens within the macrocyclic cavity, two of which are attached to nitrogen atoms. The aromatic character associated with carbaporphyrins can be attributed, at least in part, to the presence of the 18π electron circuit shown in bold for these structures (Figure 6). Less favored tautomers **155′** and **155B′** can be considered that possess internal methylene units, although these have not been observed experimentally. While these still have 18π electron delocalization pathways, benzocarbaporphyrin tautomer **155B′** can also introduce a 22π electron circuit that incorporates the fused benzo-unit. Density functional theory (DFT) calculations indicate that this delocalization pathway is favored [159,160]. Palladium(II) complexes of type **156** and **156B** effectively freeze in place the conjugation pathways found in tautomers **155′** and **155B′** and this allows extended aromatic circuits to be probed. In order to further assess how ring fusion modifies the aromatic character of carbaporphyrins, syntheses of naphtho [2,3-*b*]-21-carbaporphyrin **157** and anthro[2,3-b]-21-carbaporphyrin **158** have been developed (Scheme 27) [71,160].

Naphthocarbaporphyrin **157** was prepared by reacting diformylbenzoindane **159** with tripyrrane **11** (R = Et) in the presence of TFA, followed by oxidation with DDQ (Scheme 27) [71]. Very recently, anthracene-fused carbaporphyrin **158** was synthesized from **11** (R = *n*-hexyl) and the related dialdehyde **160** [160]. Reaction of **157** and **158** with methyl iodide and potassium carbonate in refluxing acetone gave *N*-methylcarbaporphyrins **161** and **162**, respectively, and subsequent metalation afforded, following an alkyl group migration, palladium(II) complexes **163** and **164** [71,160]. As anticipated, the spectroscopic data indicated that the aromatic conjugation pathway was extended through the fused rings facilitating 26π and 30π electron pathways. This analysis was supported by DFT calculations. The placement of the internal alkyl substituent necessitates a relocation of the π-delocalization pathway and thereby traps the structures in an arrangement that corresponds to tautomers **155N′** and **155A′**. The global conjugation pathways all follow Hückel’s rule, but the ^1^H NMR spectra indicate that the aromatic ring currents decrease as the size of the delocalization pathways increase (Table 1). When considering palladium complexes **150**, **141**, **163** and **164**, which correspond to the series shown in Figure 6, **156**, **156B**, **156N** and **156A**, the degree of deshielding to the external protons and shielding to the internal methyl groups decreases as the extent of π-conjugation increases. For instance, the resonance for the internal methyl substituent shifts downfield from −4.46 to −1.45 ppm as the size of the aromatic circuit increases, while the external *meso*-protons move upfield from values of 10.00 and 10.42 ppm in 150 to 8.84 and 9.54 ppm in **164**. The extended conjugation also leads to substantial bathochromic shifts in the electronic absorption spectra. The longest wavelength absorption for benzo-complex **141a** appears at 697 nm but this shifts to 772 nm in naphtho-derivative **163** and to 841 nm in anthracene-version **164** [71,160]. These insightful observations have been confirmed with NICS calculations and anisotropy of induced current density (AICD) plots [160].

**Scheme 28:**
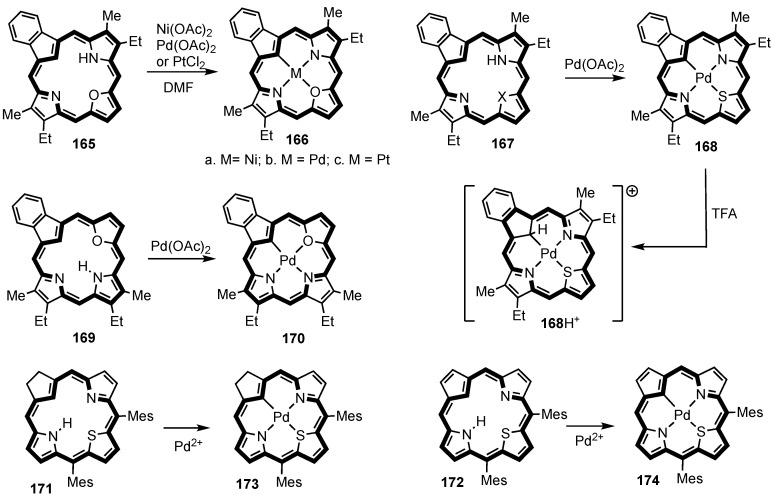
Metal complexes of oxa- and thiacarbaporphyrins.

Heterocarbaporphyrins have also been synthesized with furan, thiophene, selenophene and tellurophene rings replacing of pyrrole units [76,77,106,161]. Monoheterocarbaporphyrins act as dianionic ligands [77]. 23-Oxacarbaporphyrin **165** reacted with nickel(II) acetate, palladium(II) acetate or platinum(II) chloride in DMF to give the corresponding organometallic derivatives **166** (Scheme 28), although only low yields of the platinum(II) complex were isolated [76,77]. As expected, these metallo-derivatives retained strongly diatropic characteristics. 23-Thiacarbaporphyrin **167** similarly afforded palladium(II) complex **168**, and 22-oxacarbaporphyrin **169** also reacted with palladium(II) acetate to give palladium(II) complex **170** [162]. X-ray diffraction analysis demonstrated that palladium(II) complexes **166b** and **170** are both near planar. Addition of TFA to solutions of **168** led to the formation of an aromatic cation **168**H^+^ (Scheme 28) [84]. Thiacarbachlorin **171** and thiacarbaporphyrin **172** have also been reported to give palladium(II) complexes **173** and **174**, respectively [108].

**Scheme 29:**
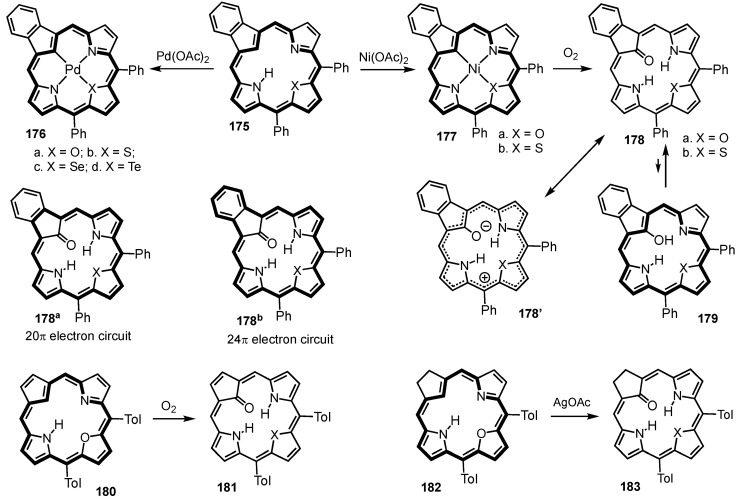
Metalation and oxidation of heterocarbaporphyrins.

Diphenyl oxa-, thia-, selena- and telluracarbaporphyrins **175a**–**d** reacted with Pd(OAc)_2_ in acetonitrile-chloroform to give a series of palladium(II) complexes **176a**–**d** (Scheme 29) [106,161]. Oxa-, selena- and telluracarbaporphyrin complexes **176a,c,d** were characterized by X-ray crystallography. The macrocyclic conformation for oxacarbaporphyrin complex **176a** is essentially planar [161]. However, the selenophene ring in **176c** is pivoted from the mean macrocyclic plane by 36.0° [161], while the tellurophene unit in **176d** is twisted away by 49.2° [106]. Nevertheless, the proton NMR spectra for all four complexes showed that they retained highly diatropic characteristics, although the UV-vis absorptions were broadened and shifted to longer wavelengths as the size of the heteroatoms increased from S to Se to Te. It is remarkable that a metal complex can be formed when a core atom as large as tellurium is present. Oxacarbaporphyrin reacted with nickel(II) acetate in refluxing DMF to afford the corresponding nickel(II) complex **177a** [161]. However, good results were only obtained when the reaction was performed under nitrogen. In the presence of air, oxidative demetalation occurred to give 21-oxocarbaporphyrinoid **178a** [161]. When pure **177a** was heated with DMF, the metalated product was converted into the carbonyl derivative and this demonstrates that oxidation only occurs following the introduction of nickel(II). Thiacarbaporphyrin **175b** also reacted with nickel(II) acetate under nitrogen to give nickel complex **177b** in 85% yield. In the presence of air, low yields of thiacarbaporphyrin oxidation product **178b** were formed. Ketones **178** are tautomers of 21-hydroxyheterocarbaporphyrins **179** but are only weakly aromatic. The system has 20 and 24 π-electron pathways, structures **178^a^** and **178^b^** that could result in antiaromatic character, but dipolar contributors such as **178′** provide 18π electron circuits that promote a degree of aromatic character [161]. Recently, oxacarbaporphyrin **180** was reported to undergo air oxidation to form a similar nonaromatic keto-structure **181** [109]. In addition, oxacarbachlorin **182** was oxidized with silver(I) acetate to give a structurally related aromatic derivative **183**; in this case, the competing antiaromatic pathways are no longer present [109].

## 6. Organometallic Chemistry of Azuliporphyrins

**Scheme 30:**
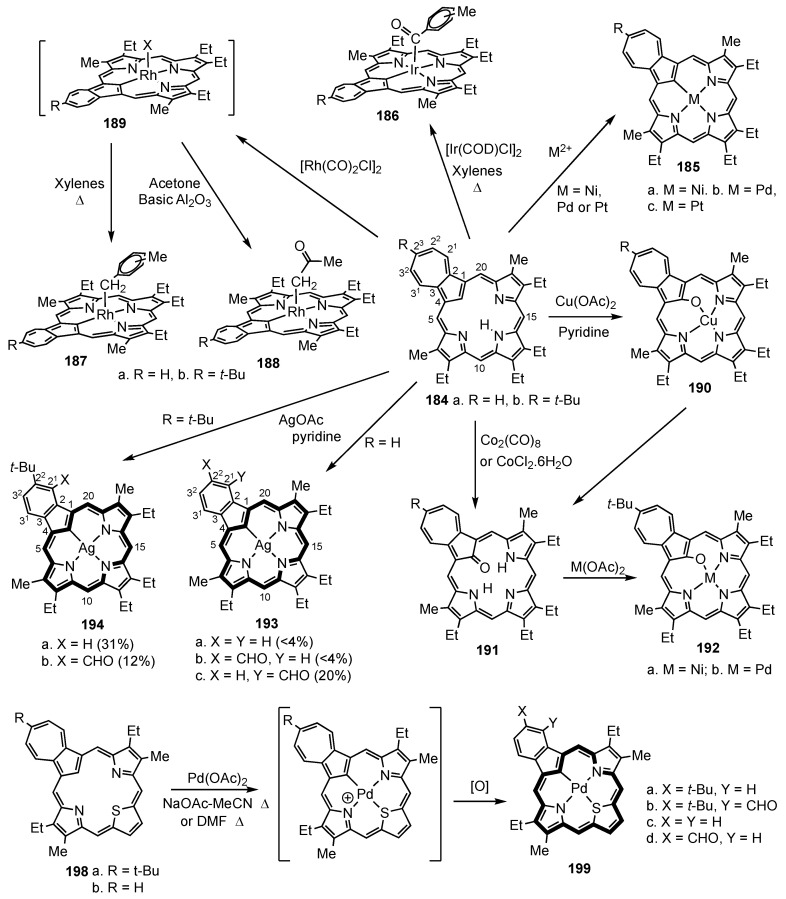
Metalation and oxidation of *meso*-unsubstituted azuliporphyrins.

**Scheme 31:**
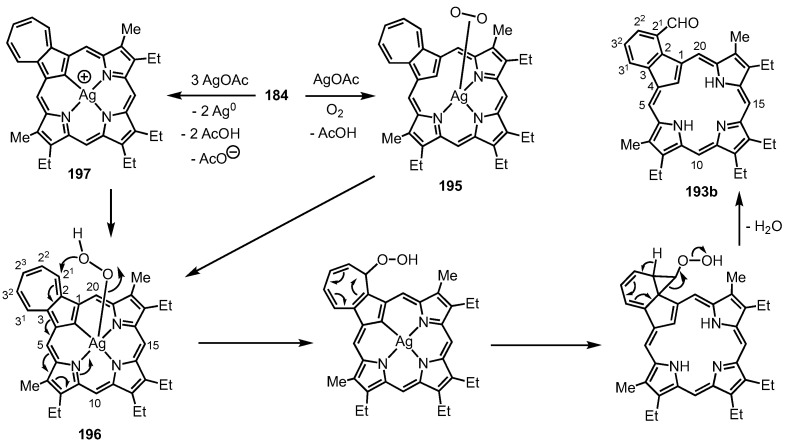
Proposed mechanism for the formation of silver(III) benzocarbaporphyrin aldehydes from the reaction of azuliporphyrins with silver(I) acetate.

Azuliporphyrins **184** (Scheme 30) have substantially reduced diatropic character compared to carbaporphyrins due to the presence of a cross-conjugated azulene unit [16,53]. However, a degree of aromatic character is retained as the proton NMR spectra for *meso*-unsubstituted azuliporphyrins shows that the inner CH proton has been shifted upfield compared to azulene to approximately +3 ppm. Nevertheless, fully aromatic carbaporphyrins commonly give a resonance for this CH at −7 ppm. The intermediary aromatic properties of azuliporphyrins have been attributed to electron-donation from the seven-membered ring which can take on a degree of tropylium character [53,163]. Unlike carbaporphyrins, azuliporphyrins are dianionic organometallic ligands. Azuliporphyrins **184** have been shown to react with nickel(II) acetate, palladium(II) acetate or platinum(II) chloride to give good yields of the corresponding metal complexes **185a**–**c** (Scheme 30) [164,165]. These stable organometallic derivatives have increased diatropicity and the *meso*-proton resonances are shifted further downfield than the values observed for free base azuliporphyrins. The largest effects are observed for palladium(II) complexes **185b**, which are considered to be the most aromatic macrocycles for this series. The *meso*-protons for platinum complex **185c** showed sidebands due to transannular coupling with ^195^Pt (^4^*J*_Pt,H_ = 4.4–5.6 Hz). *meso*-Unsubstituted azuliporphyrins **184** could also be converted into iridium(III) complexes **186** [166] and rhodium(III) complexes **187** [167] (Scheme 30). Reaction of **184** with [IrClCOD]_2_ in refluxing *o*- or *p*-xylene gave benzoyliridium(III) complexes **186**, albeit in relatively low yields, although no reaction was observed in refluxing toluene. A *tert*-butyl substituted complex gave crystals that were suitable for X-ray diffraction analysis, and this confirmed the presence of an axial aroyl unit. The macrocycle proved to be near planar and the iridium coordination environment had a 5-coordinate square pyramidal geometry. It was suggested that an iridium(III) chloride macrocyclic complex was initially formed and that this reacted with the solvent to form a benzyl iridium(III) species [166]. The proton NMR spectra showed that the benzene protons were strongly shielded by the porphyrinoid system. Further oxidation with molecular oxygen presumably converts the axial ligand into the observed benzoyl units. Reaction of **184** with [Rh(CO)_2_Cl]_2_ in refluxing *o*-, *m*- or *p*-xylene gave rhodium(III) complexes **187** with axial benzyl ligands. Again, no reaction was observed in toluene, possibly due to the lower boiling point of this solvent. Oxidation of the coordinated methylene units was not observed for the rhodium series. X-ray crystal structures were obtained for the products from all three xylene isomers and these demonstrated that the porphyrinoid macrocycles were planar with the methylbenzyl ligands occupying an orthogonal binding site [167]. The proton NMR spectra showed the coordinated methylene resonances as upfield doublets near −1.9 ppm (^2^*J*_RhH_ = 2.6–3.8 Hz). A related rhodium(III) complex **188** with an axial CH_2_C(O)CH_3_ unit was obtained when the crude rhodium(III) intermediate **189** was reacted with acetone and basic alumina in toluene. Reaction of azuliporphyrins **184** with copper(II) salts led to an oxidative metalation to form copper(II) complexes **190** [168], possibly via a copper(II) organometallic intermediate **185** (M = Cu). The structure of **190** is nonplanar and the oxyazulenyl unit is pivoted 31.76° relative to the plane described by the core nitrogen atoms. Attempts to form cobalt complexes of **184** by reacting it with CoCl_2_.6H_2_O or Co_2_(CO)_8_ were unsuccessful and instead 21-oxyazuliporphyrin **191** was generated [168]. Although reactions of **184** with Cu(II) or cobalt reagents were carried out under nitrogen, trace amounts of molecule oxygen appeared to be responsible for the formation of these oxygenated products. Oxyazuliporphyrin **191** is the keto-tautomer of 21-hydroxyazuliporphyrin. X-ray crystallography conclusively demonstrated the identity of **191** and while a 24π electron pathway is present, proton NMR spectroscopy indicates that the system is essentially nonaromatic. Protonation of this system afforded an aromatic cation. Oxyazuliporphyrin **191** acts as a dianionic ligand and reacted with nickel(II) acetate or palladium(II) acetate to afford metal complexes **192a** and **192b** [168]. These derivatives are structurally equivalent to copper(II) complexes **190** and the X-ray crystal structures for palladium(II) complex **192b** (R = *t*-Bu) was virtually superimposable with the structure obtained for copper(II) complex **190** (R = *t*-Bu). Reaction of **184** with silver(I) acetate led to the formation of silver(III) complexes of benzocarbaporphyrins (Scheme 30) [168]. Oxidative ring contraction of azuliporphyrins to benzocarbaporphyrins is well known and results in the formation of structures with unsubstituted benzo-units and related aldehydes [110]. The introduction of a formyl substituent at position 2^1^ generally only occurs to a minor extent due to steric effects. Metalation of azuliporphyrin **184** to form a silver(III) complex triggers the ring contraction but the resulting regioselectivity is greatly altered. For **184** (R = H), 2^1^-formyl derivative **193b** is the major product, albeit in 20% yield, while two other products, **193a** and **193c**, were each isolated in <4% yield. *tert*-Butylazuliporphyrin **184** (R = *t*-Bu) gave **194a** as the major product in 31% yield, but a greater than expected yield (12%) of sterically crowded benzocarbaporphyrin aldehyde **194b** was also isolated. A mechanism was proposed to explain the observed results (Scheme 31). Formation of a silver(I) complex, followed by complexation of molecular oxygen, would give **195** and a subsequent internal redox reaction would produce silver(III) complex **196** with an axial peroxide ligand. Alternatively, silver(III) azuliporphyrin cation **197** might be formed initially, followed by formation of the peroxide derivative. The location of the axial peroxide unit facilitates nucleophilic attack at the nearby 2^1^-position and subsequent Cope rearrangement and elimination of water gives the observed aldehyde product [168]. Examples of heteroazuliporphyrins with furan, thiophene or selenophene subunits have also been synthesized. Metalation of thiaazuliporphyrin **198** with palladium(II) acetate led to a similar ring contraction to form palladium(II) benzocarbaporphyrins **199** (Scheme 30) [168]. In these reactions, formation of crowded 2^1^-formyl derivatives was not favored indicating that the process did not occur via the type of directed intramolecular nucleophilic attack proposed for silver derivatives.

**Scheme 32:**
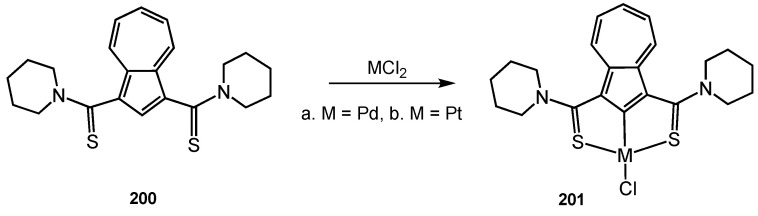
Metalation of an azulene pincer ligand.

It is worth noting that azulene-based pincer ligands have been reported that can bind divalent transition metal cations [169]. Azulene bis-thioamide **200** reacted with palladium chloride and lithium chloride in refluxing methanol to afford organometallic palladium(II) complex **201a** (Scheme 32). Reaction of **200** with PtCl_2_(PhCN)_2_ in acetonitrile generated the related platinum complex **201b**. These structures have similar features to metalated azuliporphyrins such as **185**.

**Scheme 33:**
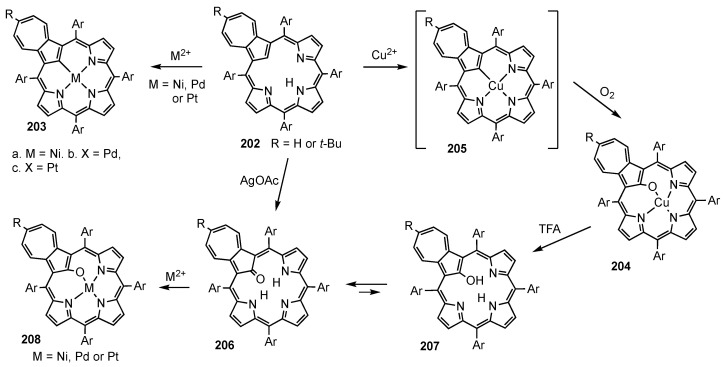
Metalation of *meso*-tetraarylazuliporphyrins.

Metalation reactions for tetraarylazuliporphyrins **202** show some significant differences to the chemistry of *meso*-unsubstituted azuliporphyrins **184**. However, they also react with Ni(OAc)_2_, Pd(OAc)_2_ and PtCl_2_ to give similar organometallic derivatives **203** (Scheme 33) [165]. Furthermore, attempts to metalate **202** with copper(II) salts led to the formation of copper(II) oxyazuliporphyrins **204** [170,171]. X-ray crystallography showed that the structure was highly distorted and the azulene ring was tilted by almost 53° relative to the mean macrocyclic plane [170]. Reactions in the presence of ^18^O_2_ demonstrated that the oxygen atom derives from molecular oxygen. A recent study demonstrated that copper(II) complex **205** can be isolated under strictly anaerobic conditions using a glove box [172]. Exposure to air then led to the formation of **204**. However, even in the absence of O_2_, conversion to **204** still takes place via inner core nucleophilic attack from water or hydroxide ions. Demetalation of **204** with 10% TFA-CHCl_3_ gave 21-oxyazuliporphyrins [170,171]. As was the case for the *meso*-unsubstituted series, keto-tautomers **206** were favored over hydroxyazuliporphyrins **207**. Metalation of **206** with Ni(OAc)_2_, Pd(OAc)_2_ or PtCl_2_ gave nickel(II), palladium(II) and platinum(II) complexes **208** [171]. X-ray crystallography showed that the conformations of the Pd(II) and Pt(II) complexes were virtually identical to the structure obtained for copper(II) oxyazuliporphyrin **204** [171].

**Scheme 34:**
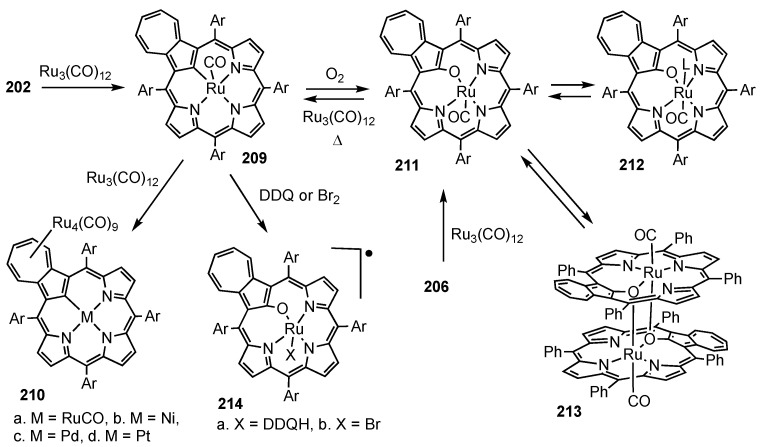
Ruthenium complexes of tetraaryl azuliporphyrins.

Ruthenium complexes of azuliporphyrins **202** have also been reported (Scheme 34) [173,174]. Reaction of **202** with one equivalent or less of Ru_3_(CO)_12_ gave ruthenium(II) complex **209**. The proton NMR spectrum showed the external pyrrolic protons between 7.62 and 7.69 ppm, indicating that the macrocycle has a moderate aromatic ring current. Addition of excess Ru_3_(CO)_12_ led to the formation of cluster complex **210a**, and related bimetallic derivatives **210b**–**d** were obtained from nickel(II), palladium(II), and platinum(II) azuliporphyrins **203a**–**c**. In the presence of air, **209** slowly oxidized to give ruthenium 21-oxyazuliporphyrin complex **211**. This complex can also be prepared in 33% yield by reacting 21-oxyazuliporphyrin **206** with Ru_3_(CO)_12_. Interestingly, **211** can be converted back into **206** by reacting it with Ru_3_(CO)_12_ in refluxing chlorobenzene. Oxyazuliporphyrin complex **211** readily added a further ligand to form hexacoordinate ruthenium complexes **212**, and a structure of this type that incorporated 1-butanol was characterized by X-ray crystallography. In the absence of a suitable ligand, a dimeric complex **213** could be isolated instead. Cyclic voltammetry showed that **209** underwent two reversible one-electron oxidations, and the first oxidation gave rise to an easily accessible radical cation **214**. This type of oxidation was also accomplished with DDQ or bromine to give the π-radical species **214a,b** (Scheme 34).

**Scheme 35:**
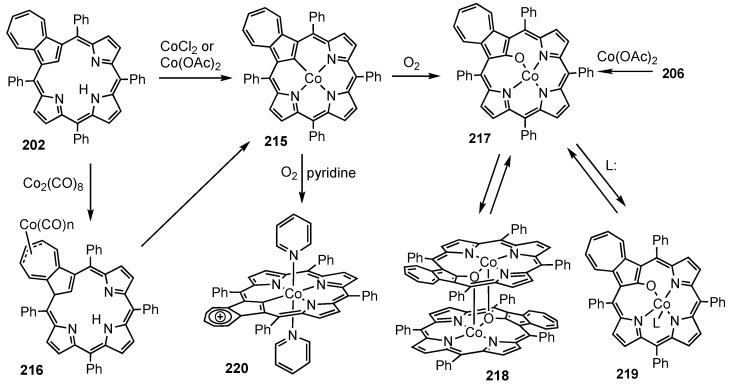
Cobalt complexes of tetraphenylazuliporphyrin.

In contrast to *meso*-unsubstituted azuliporphyrins, tetraphenylazuliporphyrin **202** reacted with CoCl_2_ or Co(OAc)_2_ to give cobalt(II) azuliporphyrin **215** (Scheme 35) [172]. In addition, treatment of **202** with Co_2_(CO)_8_ afforded a transient π-allyl complex **216** that slowly converted into **215**. When **215** was exposed to air, oxidation to cobalt(II) oxyazuliporphyrin **217** was observed. This species exists in equilibrium with dimer **218** but addition of ligands such as pyridine leads to the formation of hexacoordinate cobalt(II) complexes **219**. Reaction of oxyazuliporphyrin **206** with cobalt(II) acetate also afforded **217**. In pyridine solutions, **215** air oxidized to give cationic cobalt(III) complex **220** and the proton NMR spectrum for this structure indicated that the system had taken on significantly increased diatropicity. This can be attributed to the seven-membered ring taking on tropylium character while facilitating 18π electron delocalization pathways in the porphyrinoid ligand.

**Scheme 36:**
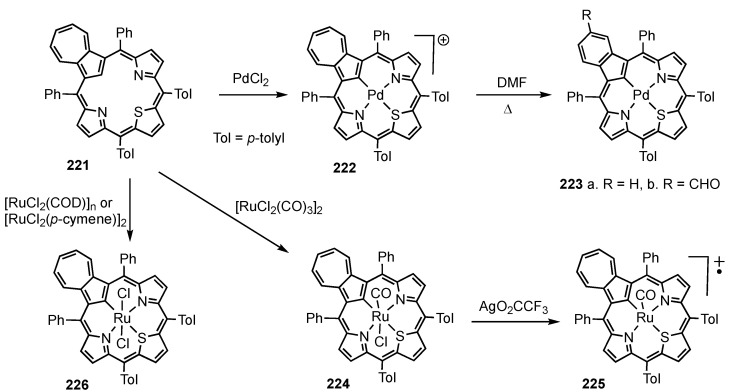
Metalation of a tetraaryl thiaazuliporphyrin.

Tetraarylthiaazuliporphyrin **221** reacted with palladium(II) chloride to give palladium(II) thiaazuliporphyrin cation **222** (Scheme 36) [175]. The X-ray structure for this species showed that the thiophene ring was deflected from the macrocyclic plane by over 30°. The electron-deficient seven-membered ring underwent ring contractions in the presence of suitable nucleophiles to generate palladium(II) thiacarbaporphyrins **223**. The best results were obtained when **222** was heated with palladium(II) acetate in DMF and ring-contraction products **223a** and **223b** were isolated in 12% and 51% yields, respectively. Thiaazuliporphyrin **221** also reacted with ruthenium reagents in chlorobenzene to give chlorocarbonylruthenium(II) complex **224**. Addition of silver(I) trifluoroacetate in dichloromethane produced the related radical cation **225**. However, when **221** was reacted with ruthenium reagents without carbonyl ligands, an oxidation reaction took place to give the paramagnetic ruthenium(III) complex **226**.

**Scheme 37:**
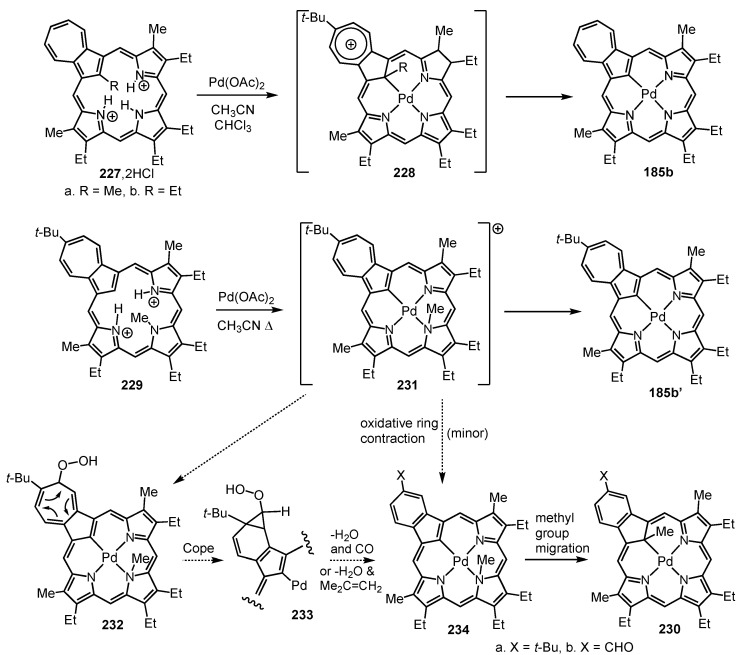
Palladium complexes derived from internally alkylated azuliporphyrins.

Metalation of internally alkylated azuliporphyrins was investigated (Scheme 37) [176]. These alkyl derivatives proved to be unstable and were isolated as the corresponding hydrochloride salts. 21-Alkylazuliporphyrins **227a**.2HCl and **227b**.2HCl were reacted with palladium acetate in refluxing chloroform-acetonitrile. A poor yield of palladium(II) complex **185b** (<10%) was isolated where the internal alkyl substituents had been lost. This may be due to nucleophilic displacement of the metalloporphyrinoid from the alkyl substituents in intermediate **228**. A second aromatic product was noted but could not be identified. The main product from the reaction of 23-methylazuliporphyrin **229**.2HCl with Pd(OAc)_2_ was also a dealkylated palladium(II) azuliporphyrin complex **185b′** (45% yield) [176]. However, two minor products corresponding to palladium(II) benzocarbaporphyrins **230a,b** were isolated in 5% and 2.4% yields, respectively. These products were profoundly modified by a combination of oxidative ring contractions and methyl group migration. It has been reported that palladium can induce the formation of peroxides from molecular oxygen [177] and it was proposed that the first step leading to the formation of **230a,b** involves nucleophilic attack from a hydroperoxide ion onto intermediate **231** to give **232** (Scheme 37) [176]. Cope rearrangement would generate a six-membered ring while closing off a cyclopropane unit affording **233**. Further elimination of water and CO would generate palladium(II) 23-methyl *tert*-butylbenzocarbaporphyrin **234a**. This would be expected to undergo a methyl group migration to afford the observed product **230a**. Alternatively, intermediate **233** could eliminate isobutylene and water to produce **234b** and this would further rearrange to give aldehyde **230b**.

**Scheme 38:**
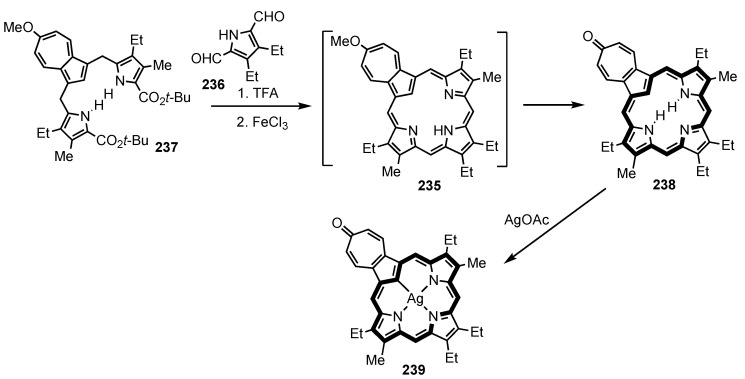
Synthesis and metalation of a tropone-fused carbaporphyrin.

In an attempt to prepare 6-methoxyazuliporphyrin **235**, pyrrole dialdehyde **236** was condensed with azulitripyrrane **237** in the presence of TFA, followed by oxidation with FeCl_3_ (Scheme 38) [178]. Unexpectedly, tropone-fused carbaporphyrin **238** was generated instead. Although the UV-vis spectrum for **238** appeared to be a hybrid of the electronic spectra for azuliporphyrins and carbaporphyrins, **238** was fully aromatic and behaved as a trianionic ligand. Specifically, **238** reacted with silver(I) acetate to give silver(III) complex **239**.

## 7. Organometallic Chemistry of Benziporphyrins, Naphthiporphyrins and Related Systems

**Scheme 39:**
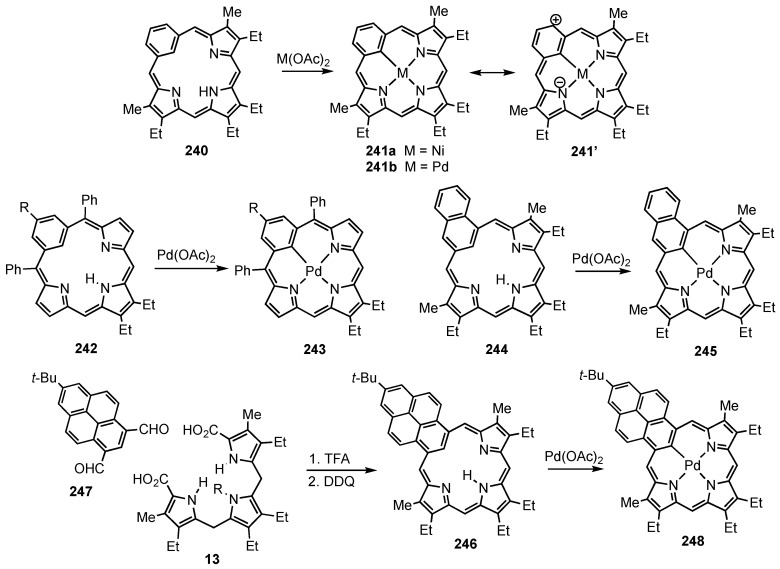
Nickel and palladium complexes of benziporphyrins.

Benziporphyrins are porphyrin analogues which have a benzene ring that replaces one of the pyrrole units [17,18,179]. Naphthiporphyrins are similar systems incorporating naphthalene rings instead. Benziporphyrins can act as monoanionic or dianionic ligands [17]. *meso*-Unsubstituted benziporphyrin **240** reacted with nickel(II) acetate in refluxing DMF to give the nickel(II) complex **241a** in 42% yield (Scheme 39) [65]. Palladium(II) complex **241b** was also obtained in 35% yield by reacting **240** with palladium(II) acetate in refluxing acetonitrile. The proton NMR spectrum for **241a** showed the *meso*-protons as two 2H singlets at 7.16 and 7.48 ppm, while palladium complex **241b** gave these resonances at 7.35 and 7.72 ppm, indicating that metallobenziporphyrins have weakly aromatic properties that are enhanced for the palladium(II) complex [65]. The aromatic properties can be attributed to dipolar resonance structures such as **241′** that possess 18π electron pathways. Diphenylbenziporphyrins **242** gave good yields of palladium(II) complexes **243** when treated with palladium(II) acetate in refluxing acetonitrile [83]. Naphthiporphyrin **244** similarly reacted with palladium(II) acetate to generate metal complex **245** [65]. The X-ray crystal structure for this complex demonstrated that the porphyrinoid macrocycle was slightly saddled. An example of a related pyreniporphyrin **246** was prepared by reacting pyrene dialdehyde **247** with tripyrrane **13** in the presence of TFA, followed by oxidation with DDQ [68]. As expected, this benziporphyrin-like structure reacted with palladium(II) acetate to produce the related palladium(II) complex **248** [68].

**Scheme 40:**
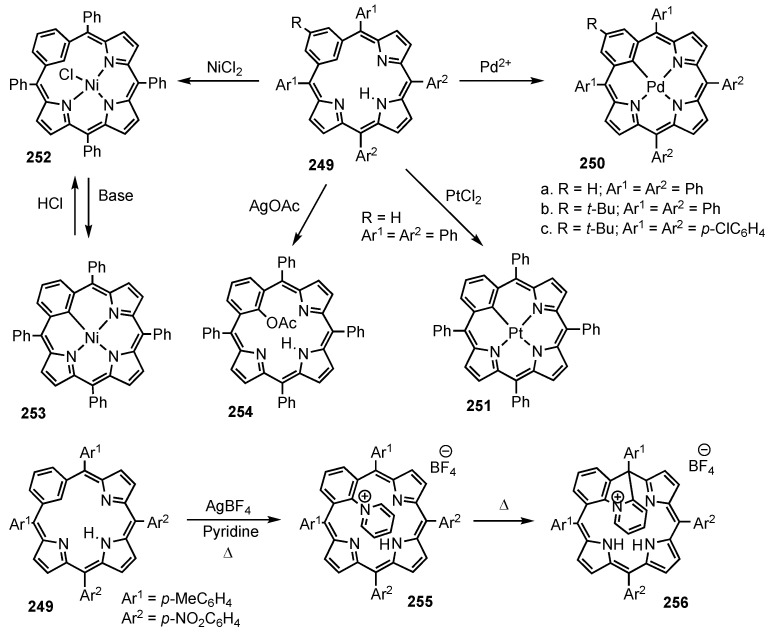
Metalation and regioselective oxidation of *meso*-tetraaryl benziporphyrins.

Most of the organometallic chemistry of benziporphyrins has been carried out using tetra-arylbenziporphyrins **249** [17]. Reaction of **249** with PdCl_2_ or Pd(OAc)_2_ in refluxing acetonitrile or CHCl_3_-CH_3_CN gave the corresponding palladium(II) complexes **250** in 49–70% yield [91,92] (Scheme 40). Tetraphenylbenziporphyrin also reacted with platinum(II) chloride in refluxing benzonitrile to afford a 20% yield of platinum complex **251** [91]. Reaction of **249** with nickel(II) chloride initially gave chloronickel(II) complex **252** but this converted into the organometallic complex **253** [180,181]. The conversion of **252** into **253** can accelerated by adding anhydrous potassium carbonate, but **253** is converted back into **252** upon treatment with dry HCl in chloroform. When **249** was treated with silver(I) acetate, a regioselective oxidation occurred to afford the 21-acetoxy-derivative **254** [91], and in a related reaction **249** was shown to react with AgBF_4_ in pyridine to give 22-pyridiniumyl derivative **255** (Scheme 40) [182]. It was proposed that a silver(III) benziporphyrin is initially formed, followed by reversible axial coordination of pyridine and reductive elimination of silver(I). When a solution of **255** in CDCl_3_ was heated for 12 h, intramolecular cyclization occurred to give phlorin cation **256**.

**Scheme 41:**
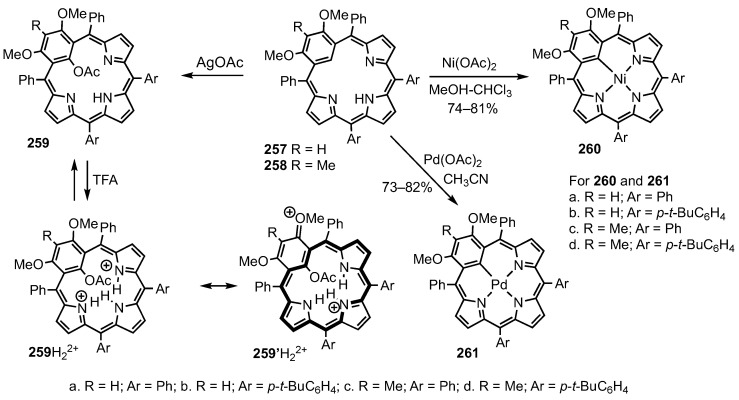
Metalation and selective oxidation of 2,4-dimethoxybenziporphyrins.

Dimethoxybenziporphyrins **257** and **258** also reacted with silver(I) acetate to give the 21-acetoxy derivatives **259** (Scheme 41) [183,184]. The proton NMR spectra for **259** (R = H) gave the acetate methyl resonance near 1.3 ppm, showing that this unit is shielded by the macrocyclic π-system. Protonation with TFA gave a dicationic species 259H_2_^2+^ where the acetate resonance shifted further upfield to 0.5 ppm, while the external pyrrolic protons shifted downfield by 0.5–0.8 ppm, indicating that the macrocycle has taken on a significant diamagnetic ring current due to resonance contributors such as **259′**H_2_^2+^ [184]. The effects were much reduced when a methyl group was present between the two methoxy substituents because steric crowding prevents the OMe units from lying coplanar with the benzene ring, diminishing electron donation. Reaction of **257** and **258** with nickel(II) acetate in refluxing chloroform-methanol gave the nickel(II) complexes **260** in 74–81% yield, while treatment with palladium(II) acetate in refluxing acetonitrile afforded palladium derivatives **261** in 73–82% yield (Scheme 41) [183,184]. The proton NMR spectra for these compounds were consistent with moderately aromatic structures. For instance, nickel(II) complex **260** (R = H, Ar = Ph), gave the pyrrolic proton resonances comparatively downfield between 7.24 and 7.66 ppm. The effect was slightly larger for palladium(II) complex **261** (R = H, Ar = Ph) and in this case the pyrrole resonances appeared between 7.30 and 7.74 ppm, The X-ray crystal structure of nickel(II) complex **260** (R = H, Ar = Ph) showed that the macrocycle has a highly distorted saddle shaped geometry. The diatropic properties of the metal complexes were substantially reduced when a methyl group was placed between the two methoxy units.

**Scheme 42:**
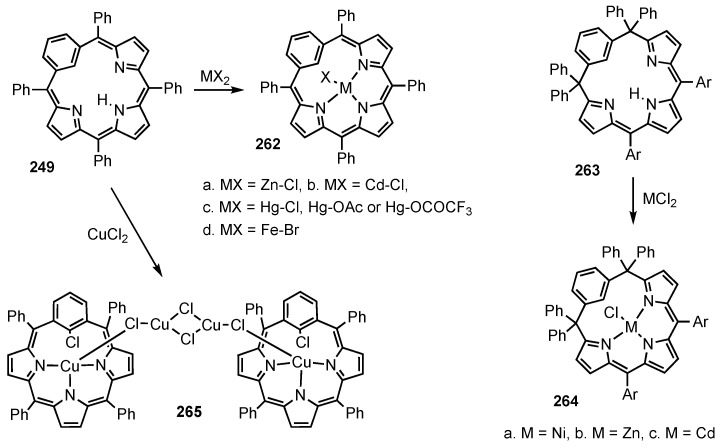
Cu, Zn, Cd, Hg and Fe complexes of benziporphyrins.

Reaction of benziporphyrins **249** with zinc chloride, cadmium chloride, or mercury salts gave metal complexes **262** (Scheme 42) [180]. Similar nickel(II), zinc, and cadmium derivatives **263** were synthesized from benziporphodimethene **264**. Evidence for agostic interactions with the internal C−H bond for complexes **262** was provided by X-ray crystallography. Reaction of benziporphyrin **249** with iron(II) bromide and lutidine in refluxing THF under an inert atmosphere afforded a high- spin iron(II) complex **262d** in 71% yield (Scheme 42) [181]. In contrast, under anaerobic conditions, copper(II) chloride reacted with **249** to give a dimeric copper complex **265**, where an oxidative chlorination had occurred on the internal carbon atoms [181]. X-ray crystallography showed that the copper(II) benziporphyrin units were connected by a [Cu_2_Cl_4_]^2−^ cluster. Dimeric silver(I) complexes of benziporphodimethenes have also been prepared [185], and a benziporphodimethene has been reported to be a selective zinc cation fluorescence switch-on sensor [186]. Furthermore, a zinc benziporphodimethene has been used as a building block to construct multidimensional nanostructure arrays [187].

**Scheme 43:**
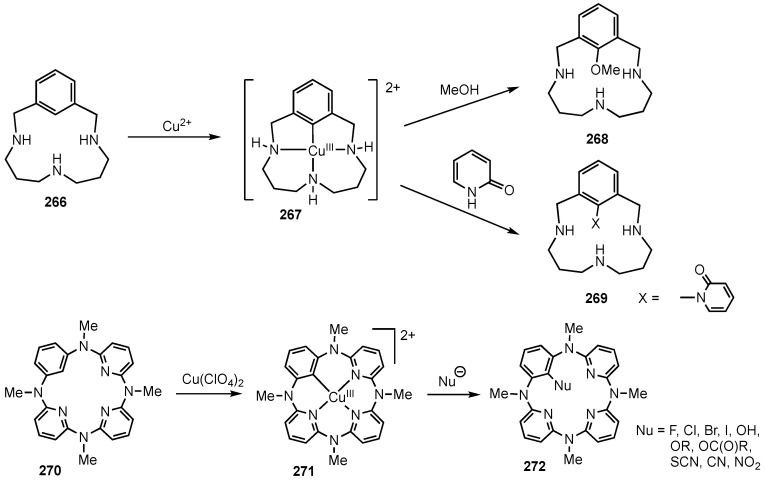
Organometallic derivatives of benzene-containing macrocycles.

Organometallic complexes for structurally related benzene-containing macrocycles have been reported. Triazamacrocycles **266** reacted with copper(II) salts to give copper(III) organometallic complexes **267** (Scheme 43) [188,189,190]. The reaction involves a disproportionation to form Cu^III^ and Cu^I^. The copper(III) can be displaced by various nucleophiles and methanol reacts to give methoxy derivative **268**, while 2-pyridone produces adduct **269**. Tetraazacalix[1]arene[3]pyridines **270** exhibit similar reactivity [191,192,193,194]. Treatment of **270** with copper(II) perchlorate gave copper(III) complex **271**, and further reaction with a variety of nucleophiles afforded substitution products **272**.

**Scheme 44:**
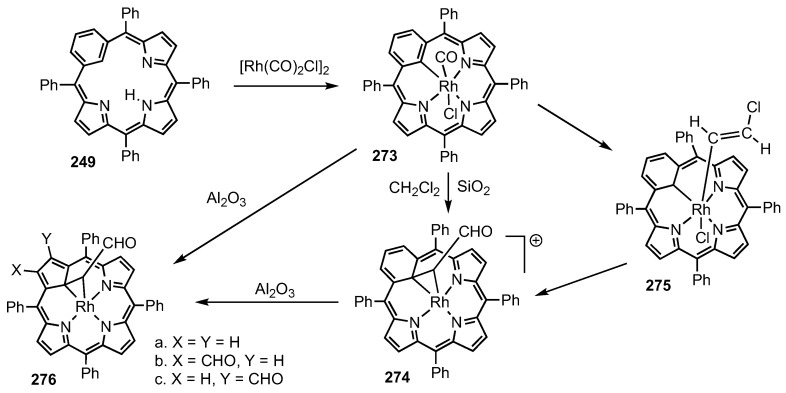
Synthesis of rhodium(III) carbaporphyrins from tetraphenylbenziporphyrin.

Reaction of tetraphenylbenziporphyrin **249** with [Rh(CO)_2_Cl]_2_ gave a six-coordinate rhodium(III) complex **273** (Scheme 44) [195]. When a solution of **273** in dichloromethane was absorbed onto a silica column and left for 12 h, aromatic rhodium complex **274** was generated. The formyl unit in **274** was shown to be derived from dichloromethane and this transformation involves the intermediacy of 2-chlorovinyl derivative **275**. Porphyrinoid **274** possesses a rhodacyclopropane unit and proved to be rather unstable. When **273** or **274** were absorbed onto basic alumina, a mixture of rhodium(III) carbaporphyrins **276a**–**c** was formed in a combined yield of 25%. The proton NMR spectrum for **276a** showed the cyclopentadiene protons downfield near 9.4 ppm, while the rhodacyclopropane proton appeared upfield at −3.5 ppm, confirming the highly diatropic nature of this system. These remarkable results demonstrate that the benzene ring in benziporphyrins can undergo a ring contraction to afford a cyclopentadiene unit. Unfortunately, the low yields of **276a**–**c** obtained from **249** makes this approach impractical for synthesizing metallo-carbaporphyrins.

**Scheme 45:**
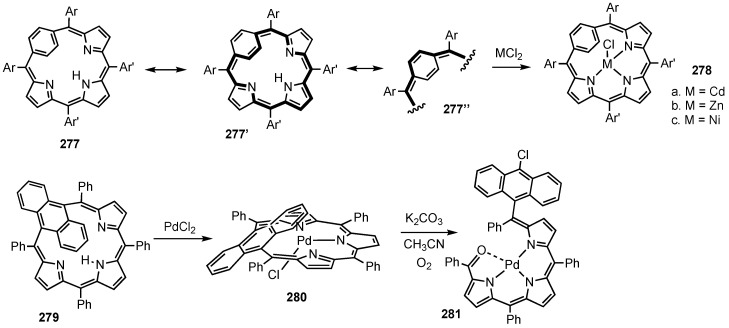
Metal complexes of *p*-benziporphyrins and anthriporphyrin.

Regular benziporphyrins, sometimes called *meta*-benziporphyrins, have the same 16-atom core as true carbaporphyrins. An isomeric system, *para*-benziporphyrin **277**, has a slightly expanded core due to the presence of a *para*-phenylene unit [96]. This system exhibits global aromatic character that has been attributed to 18π electron delocalization pathways shown in bold for resonance contributors **277′** and **277″** (Scheme 45). Nevertheless, the *p*-phenylene unit is strongly pivoted away from the mean macrocyclic plane and rapidly undergoes a teeter-tottering motion that switches the CH=CH units back and forth between the interior and exterior of the structure. *p*-Benziporphyrins **277** reacted with cadmium(II), zinc(II), and nickel(II) chlorides to give metal complexes **278a–c** [180]. Although these are only coordinated to the three core nitrogen atoms, structural evidence for *η*^2^-interactions with the phenylene unit was provided. A related anthriporphyrin **279** reacted with palladium(II) chloride in acetonitrile to yield chloropalladium complex **280**. Subsequent treatment with potassium carbonate in the presence of air resulted in oxidative ring opening to form the anthracene-appended tripyrrolic palladium(II) complex **281** [196].

**Scheme 46:**
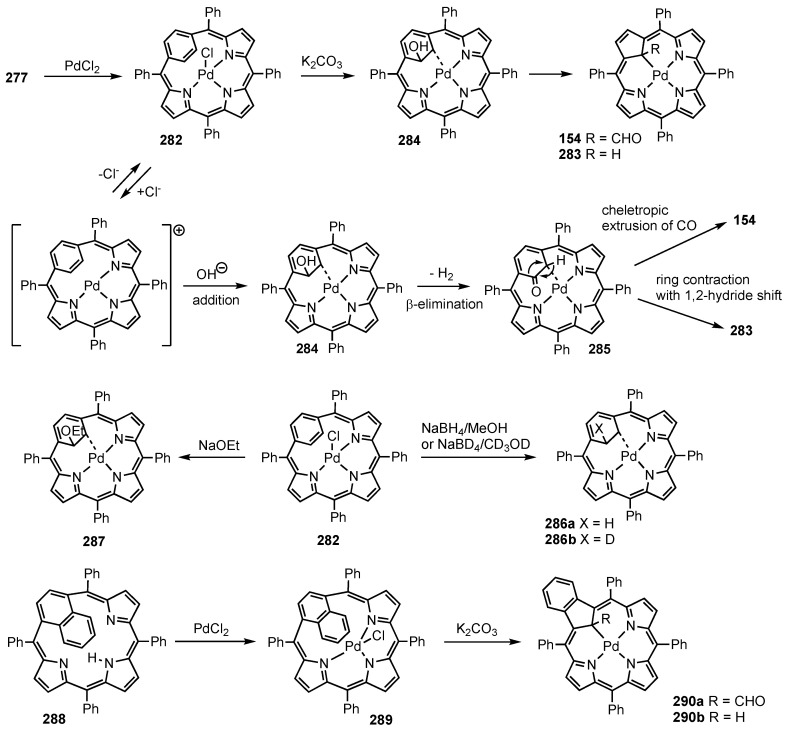
Palladium-mediated ring contractions of *p*-benziporphyrin and 1,4-naphthiporphyrin.

*p*-Benziporphyrins undergo ring contractions with transition metal cations to give palladium, rhodium and gold carbaporphyrin complexes. Reaction of **277** with PdCl_2_ in acetonitrile gave chloropalladium derivative **282** (Scheme 46) [197]. As is the case for other *p*-benziporphyrin complexes, the metal cation is involved in an *η*^2^-interaction with the arene subunit. This interaction freezes the phenylene unit in place, and the proton NMR spectrum showed the arene protons as two 2H singlets at 1.40 and 9.04 ppm, demonstrating that the complex has strongly diatropic characteristics. Treatment of **282** with potassium carbonate resulted in a rearrangement to produce palladium(II) carbaporphyrins **154** and **283** in a 1:3.5 ratio [197]. The reaction was monitored by proton NMR spectroscopy, and initial anti-addition of hydroxide and palladium to the six-membered ring was observed to give of intermediate **284** (Scheme 46). Subsequent β-elimination of H_2_ affords ketone **285**. Ring contraction involving a 1,2-hydride shift produces **283**, while extrusion of CO generates **154**. The proton NMR spectrum of formyl derivative **283** gave an upfield resonance for the aldehyde proton at 2.43 ppm, confirming the strongly aromatic properties of this complex. Reduction of **282** with sodium borohydride afforded the cyclohexadiene-palladium complex **286a**, while sodium borodeuteride stereoselectively yielded the related deuterated product **286b**. Nucleophilic addition of sodium ethoxide to palladium complex **282** gave ethoxy derivative **287**, but unlike **284**, this complex did not further react to give carbaporphyrin complexes. Structurally related 1,4-naphthiporphyrin **288** similarly reacted with palladium(II) chloride to afford chloropalladium complex **289** [198]. Both **288** and **289** had folded conformations where the naphthalene unit was placed over the porphyrinoid cavity (Scheme 46). When **289** was treated with potassium carbonate, ring contraction to give palladium(II) benzocarbaporphyrin **290a** in 21% yield together with small amounts of **290b** was observed. Although the palladium(II) cation in **290a** is placed in a square-planar coordination environment, X-ray crystallography shows that the macrocycle has a curved geometry.

**Scheme 47:**
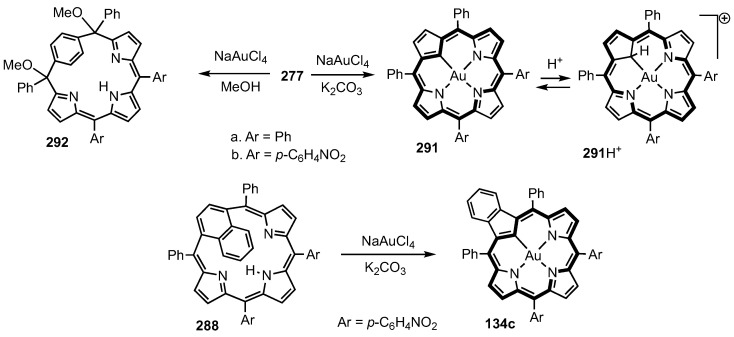
Synthesis of gold(III) carbaporphyrins from *p*-benzi- and 1,4-naphthiporphyrins.

Reaction of **277** or **288** with sodium tetrachloroaurate also induced ring contractions (Scheme 47) [199]. When *p*-benziporphyrins **277** were treated with Na[AuCl_4_]·2H_2_O and potassium carbonate, gold(III) complexes **291** were generated in 10–14% yield. Addition of acid led to a reversible protonation on the internal carbon to give cations **291**H^+^, a phenomenon that has also been reported for N-confused porphyrins [56,62,119]. Reaction of **277** with Na[AuCl_4_]·2H_2_O in methanol afforded nonaromatic dimethoxy derivatives **292** rather than the ring contraction products. 1.4-Naphthiporphyrin **288** also reacted with sodium tetrachloroaurate to give gold(III) benzocarbaporphyrin **134c** in 32% yield (Scheme 47) [199]. Similar gold(III) complexes had previously been prepared directly from tetraarylbenzocarbaporphyrins (Scheme 24) [151].

**Scheme 48:**
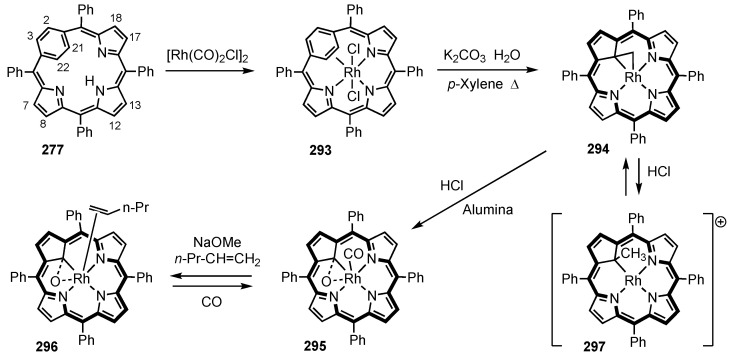
Synthesis of rhodium(III) carbaporphyrins from tetraphenyl-*p*-benziporphyrin.

Reaction of di-μ-chlorotetracarbonyldirhodium(I) with *p*-benziporphyrin **277** in toluene gave rhodium(III) complex **293** (Scheme 48) [200]. X-ray crystallographic analysis of **293** showed that the rhodium cation was close to C21–C22, suggesting the presence of a Rh−*η*^2^ interaction with the phenylene unit. The proton NMR spectrum showed the resonances for the inner and outer phenylene protons at 0.78 and 8.97 ppm, respectively, and this is consistent with a strong aromatic ring current. Furthermore, in the carbon-13 NMR spectrum the C21-C22 signal was split into a doublet due to coupling with ^103^Rh (I = ^1^/_2_). When **293** was vigorously stirred under reflux with K_2_CO_3_ and a small amount of water in *p*-xylene, ring contraction of the arene unit was observed to give rhodium(III) carbaporphyrin **294** in 46% yield. The complex contained a rhodacyclopropane unit and closely resembles a rhodium(III) complex prepared from a 23-methylcarbaporphyrin [157]. The macrocycle has a fully aromatic 18π-electron delocalization pathway, and the proton NMR spectrum showed the bridged methylene unit upfield at −3.58 ppm, while the external cyclopentadiene protons were shifted downfield to 9.14 ppm. When a solution of **294** in dichloromethane saturated with anhydrous HCl was placed on basic alumina, oxidation to oxycarbaporphyrin complex **295** was observed. The appearance of a carbonyl ligand was unexpected but may have originated from the methylene bridge in **294**. Reaction of **295** with NaOMe, followed by addition of 1-pentene, generated a related π-coordinated complex **296**. Addition of hydrochloric acid to **294** opened up the the methylene bridge to give C-methyl rhodium(III) carbaporphyrin cation **297**. This process was reversible and in the presence of water the cation reverted to **294** [200].

**Scheme 49:**
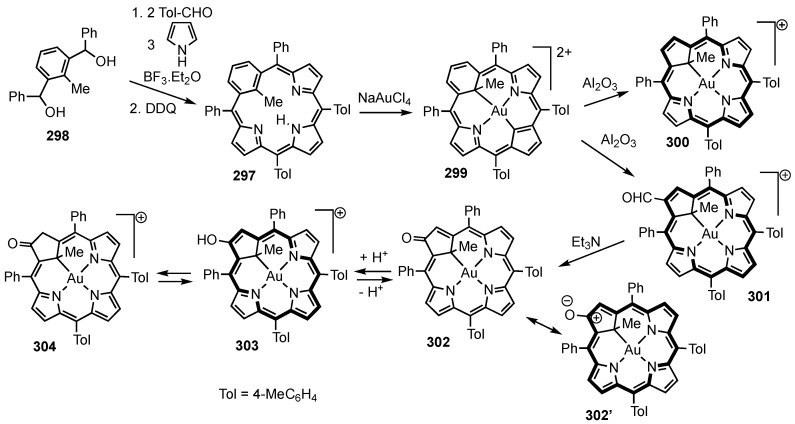
Synthesis of gold(III) carbaporphyrins from a 22-methylbenziporphyrin.

The phenylene unit in *p*-benziporphyrins undergoes some remarkable rearrangements to give carbaporphyrin organometallic complexes. However, *p*-benziporphyrins can only be synthesized in low yields and this strategy cannot be used to prepare significant quantities of carbaporphyrin derivatives. An alternative route to gold(III) carbaporphyrins was devised using 22-methylbenziporphyrins **297** [201,202]. Benziporphyrin **297** was obtained in a respectable 23% yield from dicarbinol **298** (Scheme 49) and was used to access novel carbaporphyrin derivatives. Treatment of **297** with Na[AuCl_4_].2H_2_O in dichloromethane gave a quantitative yield of chemically unstable gold(III) dication **299**. Attempts to purify **299** by column chromatography on alumina or silica resulted in the formation of gold(III) carbaporphyrins **300** and **301**. Interestingly, ring contractions mediated by alumina favored the formation of **300**, whereas silica showed a preference for aldehyde **301**. A quantitative yield of **300** and **301** was obtained when **297** was refluxed in benzene with sodium tetrachloroaurate, followed by chromatography on silica. Under basic conditions (triethylamine or potassium carbonate), **301** was converted into cross-conjugated ketone **302**. This porphyrinoid retains some aromatic character, presumably due to dipolar canonical forms such as **302′** that retain access to 18π electron delocalization pathways. Protonation with TFA or HCl resulted in the reversible formation of a cationic species **303** that showed greatly enhanced diatropic character. In particular, the proton NMR spectrum for this species gave an upfield resonance for the internal methyl substituent at −3.82 ppm, while the external pyrrolic hydrogens gave downfield peaks between 8.80 and 8.98 ppm. On the basis of deuterium exchange studies, it was proposed that the major protonated species **303** was in equilibrium with ketocarbachlorin **304** [201].

**Scheme 50:**
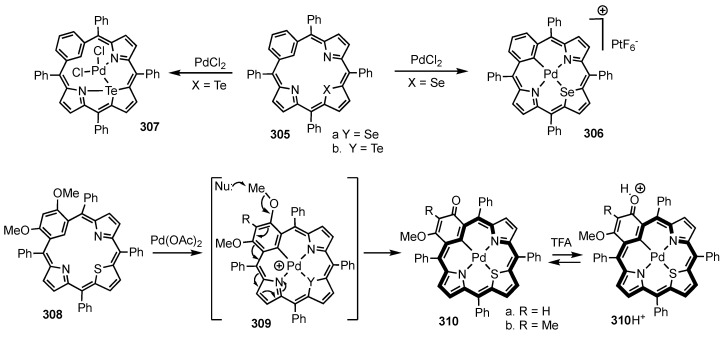
Metalation of heterobenziporphyrins.

Oxa-, thia-, selena- and tellurabenziporphyrins have been prepared and some metalation studies have been performed on these systems (Scheme 50). Selenabenziporphyrin **305a** was reported to react with palladium(II) chloride to give cationic organopalladium complex **306** [203] but the corresponding tellurabenziporphyrin **305b** afforded palladium(II) coordination complex **307** [204]. Reaction of dimethoxythiabenziporphyrin **308** initially gave palladium(II) derivative **309** but subsequent displacement of a methyl group afforded the aromatic palladium(II) oxybenziporphyrin derivative **310** [84]. The complex was reversibly protonated by TFA on the carbonyl oxygen to give cation **310**H^+^.

**Scheme 51:**
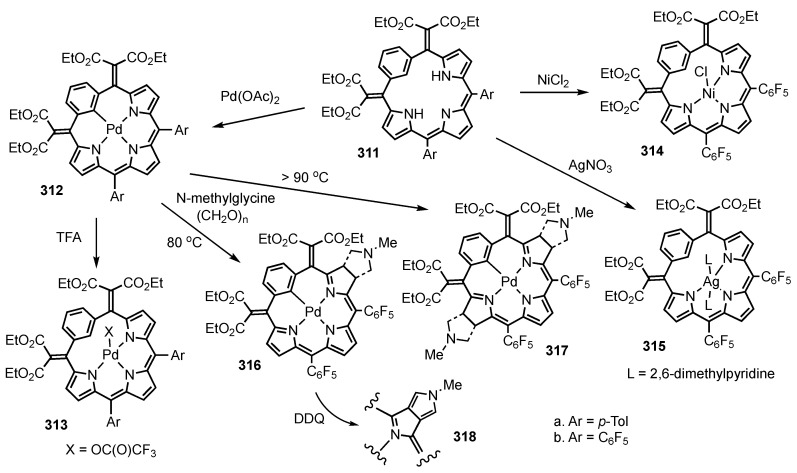
Metal complexes of benziporphyrins with exocyclic double bonds.

Lee and coworkers have investigated the synthesis and reactivity of benziporphyrins with exocyclic double bonds (Scheme 51). Benziporphyrins **311** reacted with palladium(II) chloride in refluxing acetonitrile to give palladium(II) derivatives **312** [205]. Addition of TFA to **312b** (Ar = C_6_F_5_), but not **312a** (Ar = *p*-tolyl), led to cleavage of the carbon-metal bond to form trifluoroacetate complex **313**. Reaction of **311b** with nickel(II) chloride in acetonitrile gave a similar chloronickel benziporphyrin complex **314**, while silver(I) nitrate reacted with **311b** to afford, following addition of 2,6-lutidine, silver(I) complex **315**. 1,3-Dipolar cycloadditions of benziporphyrin complex **311b** with an azomethine ylide derived from *N*-methylglycine and paraformaldehyde in toluene were investigated [206]. At 80 °C, monoadduct **316** was generated in 37% yield. However, at 90 °C or higher, the *bis*-adduct **317** was obtained in 39% yield as a diastereomeric mixture. Oxidation of **316** with DDQ afforded pyrroloporphyrinoid 318.

**Figure 7:**
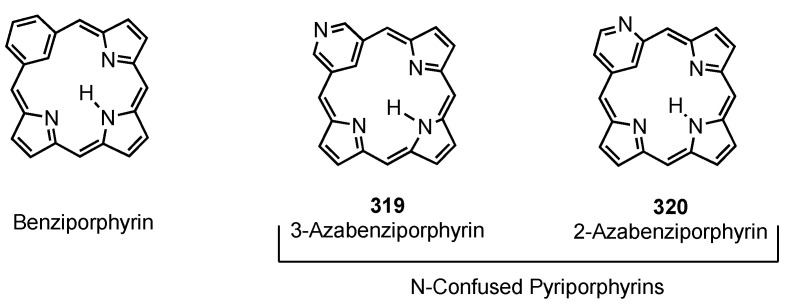
N-Confused Pyriporphyrins.

**Scheme 52:**
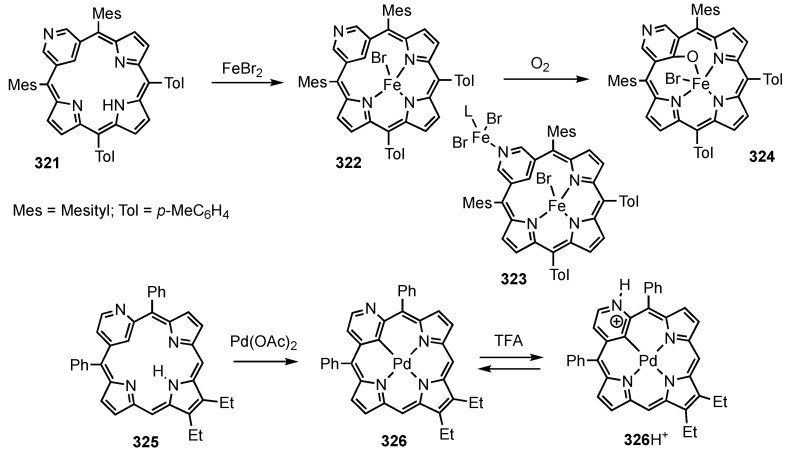
Metal complexes of N-confused pyriporphyrins.

Related systems such as inverted pyriporphyrins and nitrogen bridged porphyrinoids have also been investigated. N-confused pyriporphyrins **319** and **320**, where a pyridine ring has been inserted into the porphyrin framework so that the nitrogen faces outwards, are known and may be viewed as azabenziporphyrins (Figure 7). Importantly, the coordination cavities of N-confused pyriporphyrins are essentially the same as those found in benziporphyrins. Reaction of pyriporphyrin **321** with FeBr_2_ and collidine in THF gave iron(II) complexes **322** and **323** (Scheme 52) [207,208]. Treatment of **322** with bromine gave the corresponding iron(III) complex, while exposure to oxygen generated five-coordinate iron(III) complex **324**. Pyriporphyrin **325** reacted with palladium(II) acetate in refluxing acetonitrile to give palladium(II) complex **326** [85]. Addition of TFA afforded the externally protonated cation **326**H^+^. As expected, the proton NMR spectra for **326** and **326**H^+^ were consistent with nonaromatic porphyrinoids.

**Figure 8:**
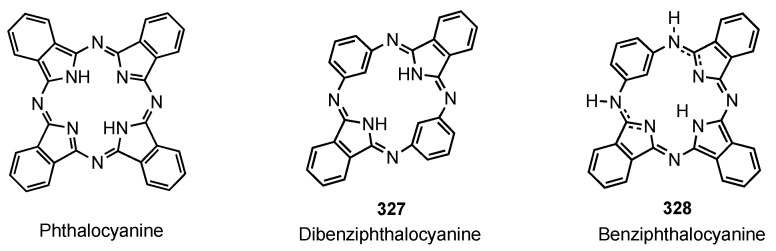
Phthalocyanine analogues.

**Scheme 53:**
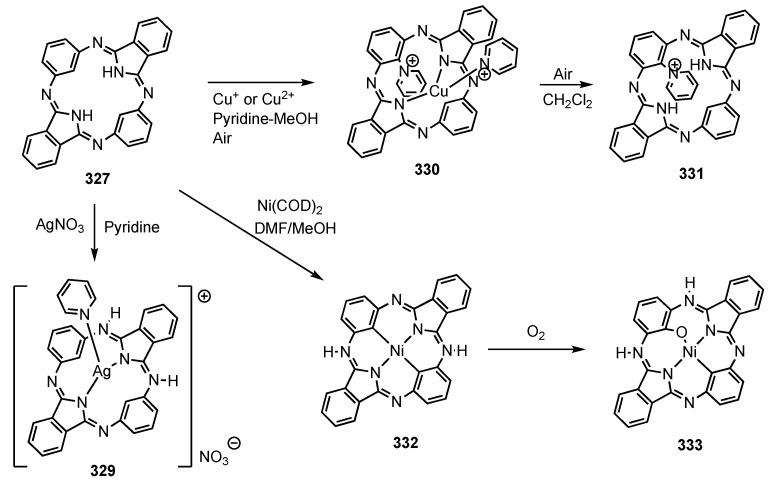
Metal complexes of dibenziphthalocyanine.

**Scheme 54:**
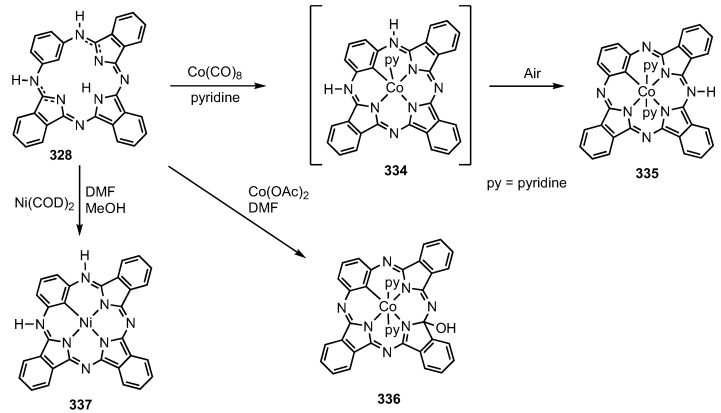
Organometallic complexes of benziphthalocyanine.

Phthalocyanines are a widely investigated group of porphyrin-like structures that have nitrogen bridges instead of the methine carbons found in porphyrins (Figure 8). Benziphthalocyanines **327** and **328** were discovered 70 years ago [209,210,211,212,213], but their potential to be organometallic ligands was only explored relatively recently. Dibenziphthalocyanine **327**, also known as dicarbahemiporphyrazine, reacted with silver(I) acetate to give silver(I) complex **329** (Scheme 53) [214]. Reaction of **327** with copper(II) and copper(I) salts gave more complicated results and afforded a copper(I) complex **330** with a pyridiniumyl group attached to a benzene ring [215]. It was proposed that copper(II)-assisted elimination of hydride resulted in the formation of the Cu(I) center. The chemistry resembles the formation of pyridiniumyl substituted porphyrinoid **255** from the reaction of benziporphyrin with silver(I) acetate and pyridine (Scheme 40) [182]. Attempts to recrystallize **330** with dichloromethane in the presence of air resulted in demetalation to form **331**. Dibenziphthalocyanine **327** formed coordination complexes with lithium, manganese, cobalt, and iron [215,216], and also afforded nickel(II) organometallic derivatives [217,218]. Reaction of **327** with Ni(COD)_2_ gave nickel(II) complex **332**, but upon exposure to molecular oxygen, a nonplanar phenolate complex **333** was generated. Benziphthalocyanine **328** readily formed cobalt and nickel(II) organometallic derivatives (Scheme 54). Reaction of **328** with Co_2_(CO)_8_ in pyridine, followed by recrystallization under anaerobic conditions, generated pyridine derivative **334**, and exposure to air afforded the corresponding six-coordinate cobalt(III) complex **335** [218]. However, when **328** was reacted with cobalt(II) acetate in DMF under aerobic conditions and the product was crystallized from *p*-xylene-pyridine, partial oxidation produced cobalt(III) complex **336** [218]. Benziphthalocyanine **328** also reacted with Ni(COD)_2_ in DMF-methanol to afford nickel(II) complex **337**. In order to form a neutral complex with Ni^2+^, it is necessary to transfer two protons onto the bridging nitrogens; relocation of protons onto bridging nitrogens is a common feature for complexes derived from **327** and **328**.

## 8. Oxybenziporphyrins, Oxynaphthiporphyrins and Related Systems

**Scheme 55:**
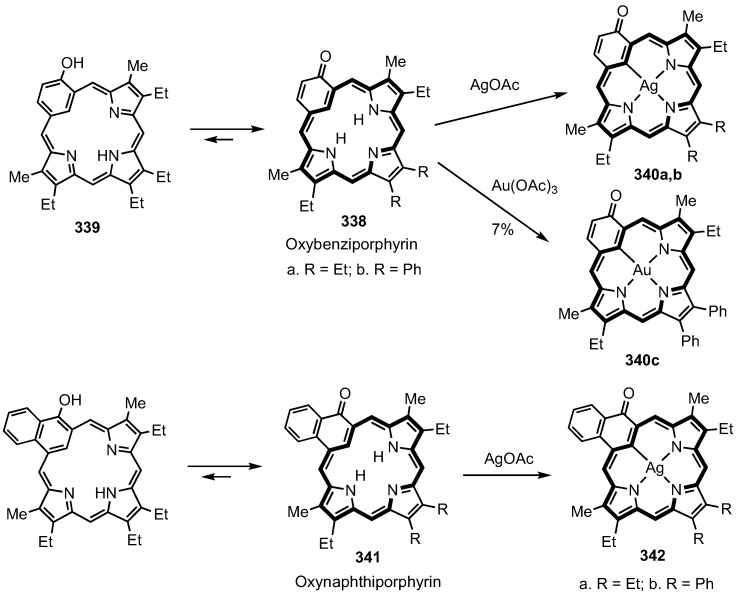
Silver(III) complexes of oxybenzi- and oxynaphthiporphyrins.

**Scheme 56:**
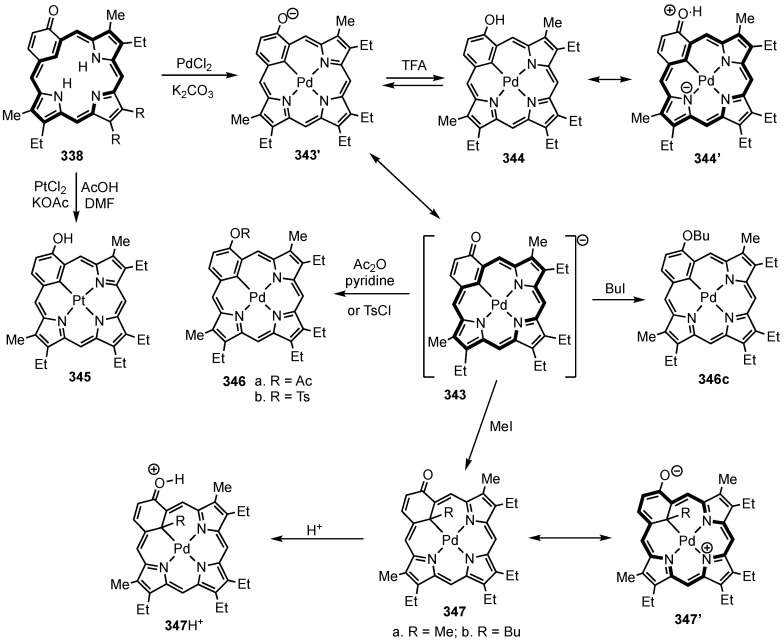
Palladium(II) and platinum(II) complexes of oxybenziporphyrin.

Oxybenziporphyrins **338** are the favored keto-tautomers of 2-hydroxybenziporphyrins **339** and can act as dianionic or a trianionic ligands [19]. Oxybenziporphyrins **338** reacts with silver(I) acetate to give the silver(III) complexes **340a,b** (Scheme 55) [65,153]. Closely related oxynaphthiporphyrins **341** reacted in the same way to give silver(III) organometallic derivatives **342**. Attempts to prepare gold(III) complexes were far less successful but a 7% yield of **340c** was obtained by reacting **338b** with gold(III) acetate [65]. These complexes retain the aromatic characteristics associated with oxybenzi- and oxynaphthiporphyrins. When **338a** was reacted with one equivalent of palladium(II) chloride in the presence of potassium carbonate, an aromatic anion **343** was generated (Scheme 56) [219]. Although this species might be expected to be a cross-conjugated phenolate anion **343′**, the proton NMR spectrum showed the *meso*-protons downfield between 9.08 and 10.37 ppm, values that are consistent with a strongly diatropic macrocycle incorporating an 18π electron delocalization pathway. Addition of one equivalent of TFA converted **343** to palladium(II) hydroxybenziporphyrin **344**. The diatropic character of **344** was much reduced compared because dipolar canonical forms such as **344′** are less favorable. A related platinum complex **345** was prepared by reacting **338** with PtCl_2_ and KOAc in mixtures of DMF and acetic acid (Scheme 56) [220]. Anionic palladium(II) complex **343** is an ambident nucleophile that can react on the oxygen or the inner carbon atom [219]. Treatment of **343** with acetic anhydride and pyridine afforded acetate **346a**, while reaction with *p*-toluenesulfonyl chloride gave the related *p*-toluenesulfonate **346b** (Scheme 56). However, reaction of **343** with methyl iodide generated C-methylated product **347a**, although treatment with *n*-butyl iodide afforded a mixture of the C-alkylated derivative **347b** and the *O*-alkylation product **346c** (Scheme 56). Unexpectedly, C-alkylation products **347a,b** were highly diatropic, possibly due to dipolar resonance contributors such as **347′** [219]. The proton NMR spectrum for **347a** gave four downfield 1H singlets for the *meso*-protons at 9.20, 9.22, 9.25 and 10.40 ppm, while the internal methyl group produced an upfield 3H singlet at −2.00 ppm. Protonation with TFA generated the aromatic cation **347**H^+^.

**Scheme 57:**
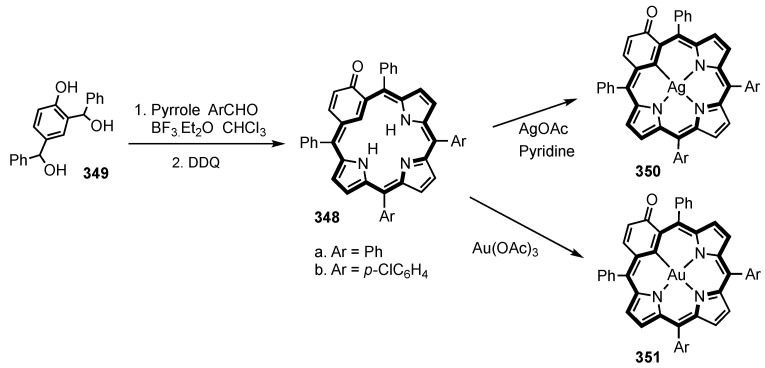
Silver(III) and gold(III) complexes of *meso*-tetraaryl-oxybenziporphyrins.

Tetraaryloxybenziporphyrins **348** were synthesized by reacting phenolic dicarbinol **349** with pyrrole and aromatic aldehydes in the presence of boron trifluoride etherate, followed by oxidation with DDQ [221]. Reaction of **348** with silver(I) acetate in pyridine gave the related silver(III) complexes **350**, while treatment with gold(III) acetate generated the gold(III) derivatives **351** (Scheme 57). Gold(III) complexes could only be obtained in low yields for *meso*-unsubstituted oxybenziporphyrins but the presence of *meso*-substituents protects these porphyrinoids from oxidative degradation and yields of 67–83% were obtained for **351** [221]. Both silver(III) and gold(III) oxybenziporphyrins exhibited strongly diatropic properties, although the aromatic character was slightly enhanced for the gold complexes.

**Scheme 58:**
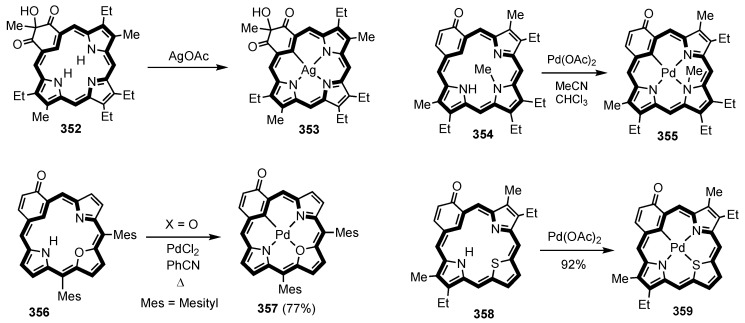
Metal complexes of ligands related to oxybenziporphyrins.

Further oxidized benziporphyrin systems have also been prepared [57,81,82] and diketone **352** acted as a trianionic ligand, reacting with silver(I) acetate at room temperature to give the silver(III) complex **353** in 87% yield (Scheme 58) [81,82]. 24-Methyl-oxybenziporphyrin **354** is a dianionic ligand and gives a fully aromatic palladium(II) complex **355** [40]. Hetero-oxybenziporphyrins have also been prepared and these are also dianionic ligands. Oxa-oxybenziporphyrin **356** reacted with palladium(II) chloride in benzonitrile to give palladium(II) complex **357** (Scheme 58) [222]. Thiaoxybenziporphyrin **358** reacted under milder conditions with palladium(II) acetate in refluxing chloroform-acetonitrile to give a similar palladium(II) complex **359** [84]. Complexes **355**, **357** and **359** all retained fully aromatic characteristics.

**Scheme 59:**
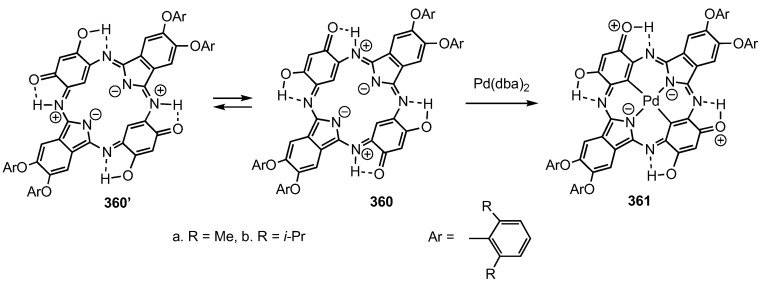
Palladium complexes of a zwitterionic dibenziphthalocyanine.

Phthalocyanine analogues of oxybenziporphyrins have also been prepared [223,224]. Bis-resorcinol containing macrocycles **360** of this type were converted into organopalladium complexes (Scheme 59). Specifically, metalation of **360a** and **360b** with bis(dibenzylideneacetone)palladium(0) gave palladium(II) complexes **361a** and **361b** in 51% and 79% yields, respectively [225]. The X-ray structure indicated that they retain the ligand’s bisquinoidal structure, and the palladium(II) complexes appeared to have zwitterionic characteristics.

## 9. Miscellaneous Monocarbaporphyrinoids

**Scheme 60:**
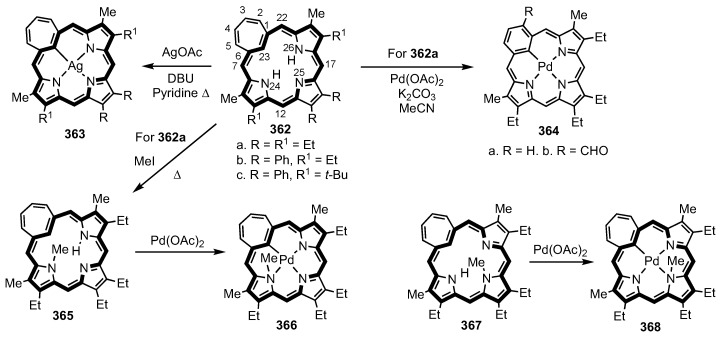
Metalation of tropiporphyrins.

**Scheme 61:**
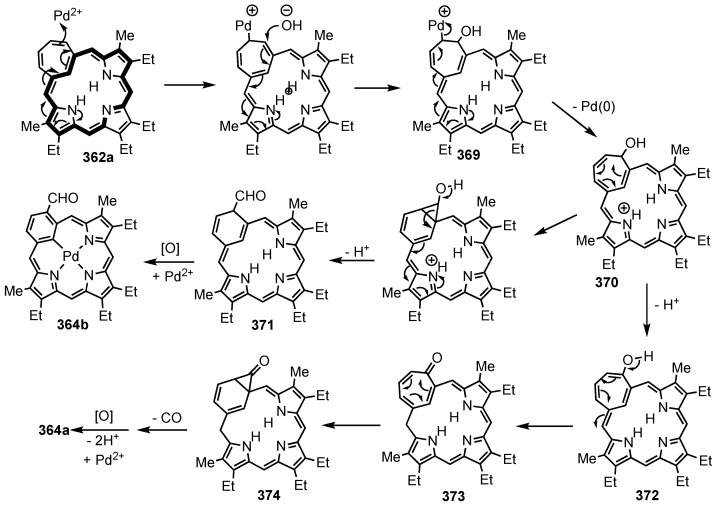
Proposed mechanisms for the formation of palladium(II) benziporphyrins from tropiporphyrin.

Tropiporphyrins **362** are trianionic ligands and react with silver(I) acetate and DBU in refluxing pyridine to give the silver(III) complexes **363** (Scheme 60) [22]. Although these derivatives are diatropic in character, the macrocycle is quite distorted. A single crystal X-ray diffraction analysis for **363b** showed that the tripyrrolic component was somewhat ruffled, but the cycloheptatriene ring was severely twisted. Reaction of tropiporphyrin with palladium(II) acetate primarily led to decomposition [226]. However, when **362a** was reacted with palladium(II) acetate in dichloromethane in the presence of potassium carbonate at 5 °C, two benziporphyrin products, **364a,b**, were isolated in a combined yield of 19% [226]. Although ring contractions of azuliporphyrins and benziporphyrins had been observed previously, this result was unprecedented. Reaction of **362a** with methyl iodide and potassium carbonate in refluxing acetone afforded 24-methyl tropiporphyrin **365**. When **365** was reacted with palladium(II) acetate, palladium(II) tropiporphyrin **366** was obtained in 48% yield and rearranged products were not observed [226]. However, it was important to limit the reaction time to 5 min at room temperature to avoid extensive decomposition. In addition, 25-methyltropiporphyrin **367** was converted to the corresponding palladium complex **368** in 43% yield under the same conditions [40]. Mechanisms for the ring contractions were proposed (Scheme 61) [226]. Addition of Pd^2+^ to the seven-membered ring of **362a**, possibly involving the initial formation of a π complex, followed by nucleophilic addition of hydroxide, would give **369**, and subsequent elimination of palladium(0) will lead to hydroxy-derivative **370**. Cope rearrangement and ring opening of the resulting cyclopropane unit will produce dihydrobenziporphyrin aldehyde **371** and subsequent oxidation and metalation would then afford **364b**. Alternatively, loss of a proton from **370** gives hydroxycycloheptatriene **372**, and following a tautomerization step, tropone **373** will be generated. Cope rearrangement can give rise to cyclopropanone **374**, and following extrusion of CO, oxidation and metalation, **364a** will be formed.

**Scheme 62:**
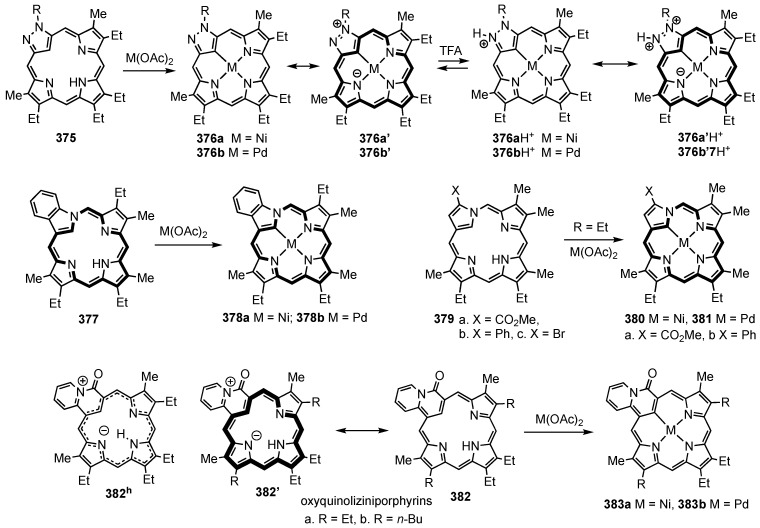
Metal complexes of pyrazoloporphyrins, neo-confused porphyrins and oxyquinoliziniporphyrins.

Carbaporphyrinoids **375** with pyrazole subunits reacted with nickel(II) acetate and palladium(II) acetate to give the organometallic derivatives **376a,b**, respectively (Scheme 62) [63,64]. Pyrazoloporphyrins **375** and their metalated derivatives are cross-conjugated and are only weakly aromatic [64]. However, the proton NMR spectra for these structures indicate a slight increase in diatropicity for the metalated structures, particularly for palladium(II) complexes **376b**. However, addition of TFA to solution of **376a** or **376b** gave monocations **376a**H^+^ and **376b**H^+^ that showed virtually no aromatic character. The weak aromatic properties for free base **375** and metal complexes **376a,b** were attributed to dipolar resonance contributors such as **375′**, **376a′** and **376b′** that possess 18π electron delocalization pathways, but protonated complexes **376a**H^+^ and **376b**H^+^ do not favor these canonical forms because it is necessary to place two positive charges next to one another (see structures **376a′**H^+^ and **376b′**H^+^) [64].

**Figure 9:**
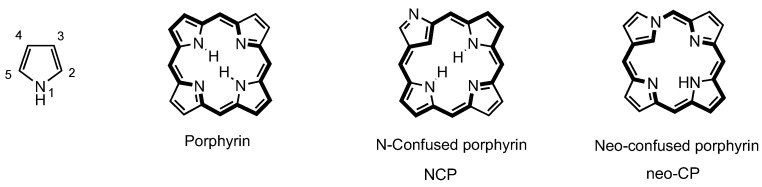
Structures of porphyrin and two of its constitutional isomers.

In N-confused porphyrins, the “confused” pyrrole ring has been connected at the 2.4-positions instead of the usual 2.5-positions (Figure 9) [88,89,90]. Neo-confused porphyrins (neo-CPs) are a more recent addition to the porphyrin isomer family in which one of the pyrrole units is connected at the 1.3-positions so that a nitrogen is directly linked to a bridging methine carbon [159,227]. This system has a 17-atom 18π electron delocalization pathway and possesses an internal CH. Benzo-neo-confused porphyrin **377** was shown to react with nickel(II) and palladium(II) acetate in acetonitrile to give the corresponding organometallic derivatives **378a,b**, respectively (Scheme 62) [88,90]. Similarly, neo-CP methyl ester **379a** gave stable nickel(II) and palladium(II) complexes **380a** and **381a** [90]. The X-ray crystal structures of **378a,b**, **380a** and **381a** showed that all four complexes are essentially planar. Proton NMR spectroscopy suggests that there is a slight increase in diatropic character for the metal complexes compared to the parent carbaporphyrinoid ligand. However, these structures have reduced aromatic ring currents compared to regular porphyrins, carbaporphyrins or N-confused porphyrins. Neo-CPs **379b,c** with phenyl or bromo-substituents instead of an electron-withdrawing ester moiety were also prepared but these were somewhat unstable [228]. However, phenyl neo-CP **379b** could also be converted into the related nickel(II) and palladium(II) complexes, **380b** and **381b**. Another recent addition to carbaporphyrinoid systems are quinoliziniporphyrins **382** [75]. This system has intermediary global aromatic character and the upfield shift of the internal proton resonance to between 3.0 and 3.5 ppm in their proton NMR spectra is similar to the results obtained for azuliporphyrins **184**. The UV-vis spectrum for **382** is also surprisingly similar to spectra obtained for **184** as well. The aromatic character associated with **382** can be ascribed to dipolar resonance contributors such as **382′** or hybrid species **382^h^** with an 18π electron circuit due to the presence of an anionic [17]annulene substructure. Oxyquinoliziniporphyrins **382** are dianionic ligands and reacted with nickel(II) and palladium(II) acetate to give the related metalated derivatives **383a** and **383b**, respectively [75].

**Scheme 63:**
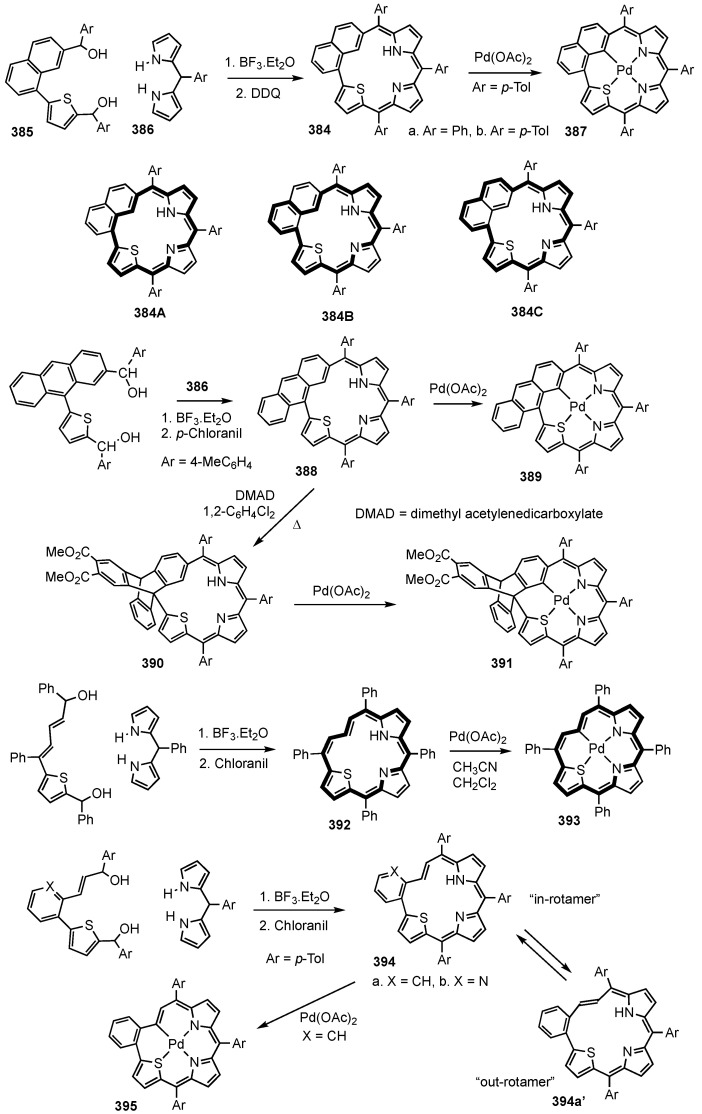
Palladium(II) complexes of a *meso*-fused thiabenziporphyrin, allyliporphyrin and related systems.

Ring-fused thiabenziporphyrins **384** were prepared by condensing thienylnaphthalene dicarbinols **385** with dipyrrylmethane **386** in the presence of boron trifluoride etherate, followed by oxidation with DDQ (Scheme 63) [229]. The resulting “*meso*-fused carbaporphyrins” **384** were isolated in 10–15% yield. This system has 18π- and 22π-electron pathways (**384A**–**C**), which give *meso*-fused carbaporphyrins moderate diatropic character. The proton NMR spectra for **384** showed that the external naphthalene, pyrrole, and thiophene protons in the range of 7.3–9.5 ppm, and broad upfield peaks at 3.72 and 4.33 ppm were assigned to the internal CH and NH protons. Reaction of **384b** with palladium(II) acetate afforded a palladium(II) complex **387** in 69% yield. Single-crystal X-ray diffraction showed that the thiophene unit was tilted relative to the mean macrocyclic plane by 38.19°. A π-extended anthracene-embedded porphyrinoid **388** was also reported and this gave the related palladium(II) complex **389** [230]. *meso*-Fused anthriporphyrin reacted with diethylacetylenedicarboxylate in 1,2-dichloromethane to give Diels-Alder adduct **390** in 46% yield and this also reacted with palladium(II) acetate to give organometallic derivative **391**. A related, more flexible, system **392** called allyliporphyrin has been prepared and this also gave excellent yields of the corresponding palladium(II) complex **393** (Scheme 63) [231]. Porphyrinoids **394** with intermediary structures between **384** and **392** have been described [232]. As is the case for **392**, benzo-fused allyliporphyrin **394a** is in equilibrium with alternative conformations, or tautomers, in particular the alternative aromatic species **394a′**. The latter structure was favored in the solid state. The presence of a nitrogen in pyrido-fused structure **394b** relieves steric interactions with the adjacent vinylene hydrogen atom, and this results in enhanced diatropic characteristics. Reaction of **394a** with palladium(II) acetate afforded the corresponding palladium(II) complex **395**, thereby locking the aromatic conformation in place.

**Scheme 64:**
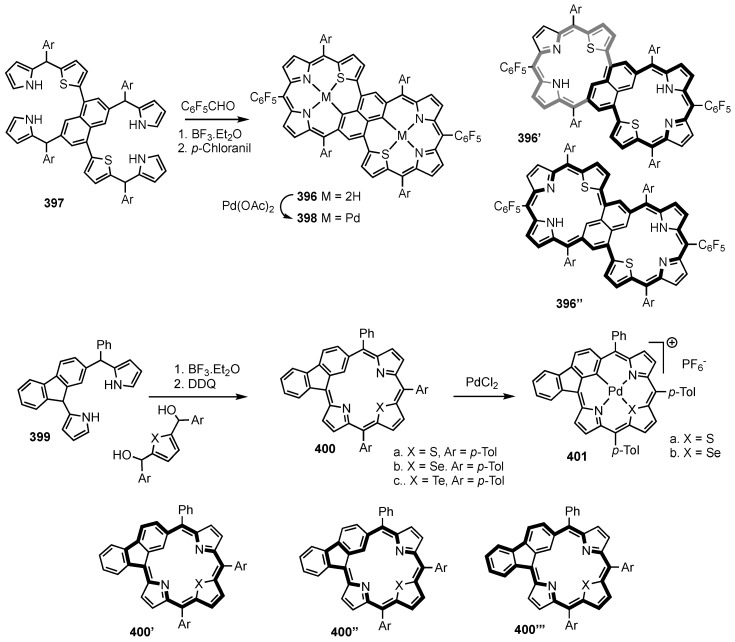
Metalation of unusual ring-fused thiabenziporphyrins.

An interlinked thianaphthiporphyrin dimer **396** was prepared from bis-naphthibilane **397** in 2% yield (Scheme 64) [233]. Although cross-conjugated, proton NMR spectroscopy indicates that the system is weakly diatropic. Anisotropy of induced current density (AICD) plots indicate that this is primarily due to the 22 and 34π electron pathways shown in bold for structures **396′** and **396″**. Dimer **396** reacted with palladium(II) acetate at room temperature to give *bis*-palladium complex **398** in 97% yield. A weakly antiaromatic heterobenziporphyrin system with embedded fluorene units was prepared using the same methodology (Scheme 64) [234]. Tripyrrane analogue **399** underwent BF_3_.Et_2_O catalyzed condensation with thiophene, selenophene or tellurophene dicarbinols to give, following oxidation with DDQ, indeno-heterobenziporphyrins **400**. The proton NMR spectra for **400a**–**c** showed that these porphyrinoids have paratropic ring currents resulting in downfield shifts to the internal CH resonances and small upfield shifts to the external protons. Thiabenziporphyrin **400a** gave the inner CH resonance at 12.03 ppm. Potential π-electron circuits with 20 and 24π electron pathways, as shown in structures **400′**, **400″** and **400‴**, may be responsible for this effect. Similar considerations may also be responsible for the reduced aromatic character of indenoporphyrins [235,236]. When reacted with palladium(II) chloride, thia- and selenabenziporphyrins **400b** and **400c** gave cationic organopalladium derivatives **401** [234].

**Scheme 65:**
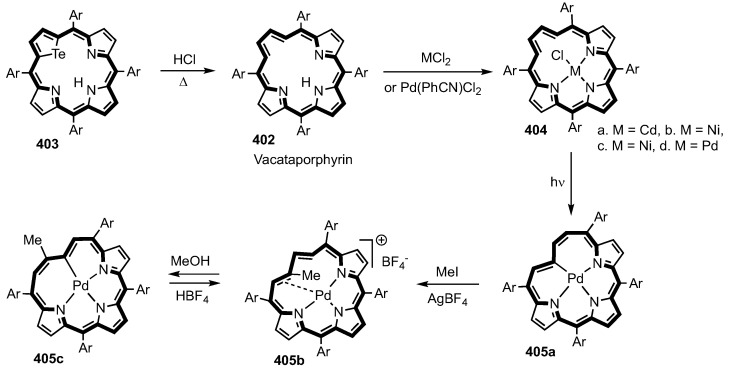
Palladium complexes of vacataporphyrin.

This review focuses on the metalation of carbaporphyrinoids that share the same 16-atom core arrangement as porphyrins and true carbaporphyrins. However, other closely related systems have some bearing as these may give insightful complementary results. Vacataporphyrins **402** are a case in point [237]. Although this system no longer has the 16-atom core it essentially shares the porphyrin framework minus one of the core atoms. Vacataporphyrins, or deazaporphyrins, were prepared by heating telluraporphyrins **403** with hydrochloric acid at 180 °C (Scheme 65). Vacataporphyrins have similar 18π electron delocalization pathways to regular porphyrins and are strongly aromatic. They react with cadmium(II), nickel(II) and zinc chloride to afford coordination complexes **404a**–**c** [238] and treatment with Pd(PhCN)_2_Cl_2_ gave a similar palladium complex **404d**. Exposure to light led to the formation of a carbon-palladium bond and generated aromatic complex **405a** [239]. Reaction with methyl iodide in the presence of AgBF_4_ produced the C-methylated cationic palladium(II) complex **405b**, and upon heating with methanol deprotonation resulted in the formation of **405c**. The proton NMR spectrum for **405b** was consistent with a paratropic system due to the conformation facilitating Möbius-type antiaromaticity. Complex **405c** is aromatic but this species can be converted back into **405b** by treatment with fluoroboric acid.

## 10. Dicarbaporphyrinoid Systems

**Figure 10:**
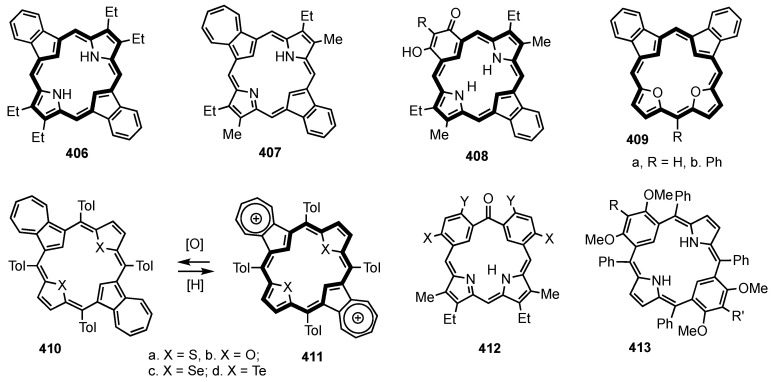
Selected dicarbaporphyrinoid systems.

**Scheme 66:**
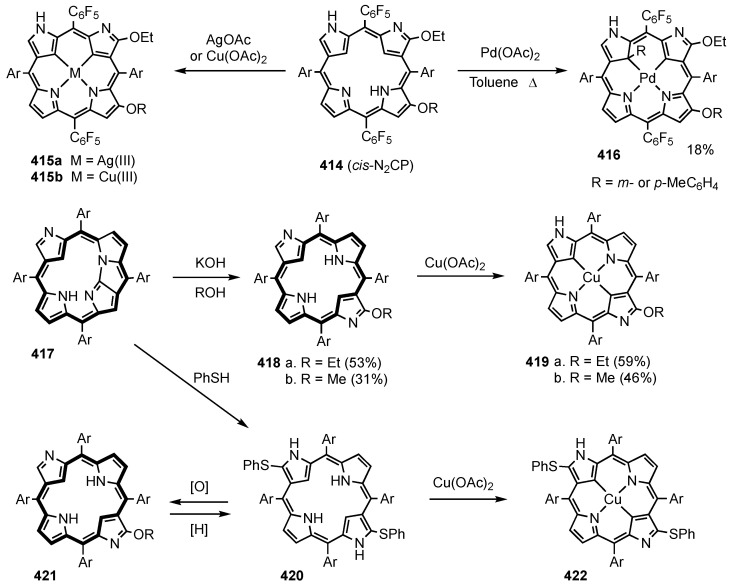
Metalation of doubly N-confused porphyrins.

Dicarbaporphyrinoid systems have two of the nitrogens within a porphyrin-type cavity replaced by carbon atoms. The first example of this class, 21,23-dicarbaporphyrin **406**, was reported in 1999 [240], but many other examples (e.g., **407**–**413**) have been reported over the last 23 years (Figure 10) [78,84,87,241,242,243,244,245,246,247,248,249]. Many of these porphyrinoids are less stable than monocarbaporphyrins and the metalation reactions for these systems has not been explored to the same extent. Nevertheless, interesting examples of metalated dicarbaporphyrinoids have been reported. *cis*-Doubly N-confused porphyrin (*cis*-N_2_CP, **414**) reacted with silver(I) acetate in 10% pyridine-chloroform to give silver(III) complex **415a**, and copper(II) acetate reacted similarly to afford the copper(III) derivative **415b** (Scheme 66) [250,251,252,253]. The reaction of *cis*-N_2_CP **414** with palladium(II) acetate in refluxing toluene gave a more unusual result, affording a palladium(II) species **416** that had undergone arylation onto an internal carbon atom [254]. A mixture of *meta*- and *para*-tolyl isomers were observed in a ratio of 2:1. When N-fused porphyrinoid **417** was treated with potassium hydroxide in ethanol or methanol, ring opening produced alkoxy-substituted *trans*-N_2_CPs **418a,b** in 53% and 31% yields, respectively (Scheme 66) [255]. Proton NMR spectra demonstrated that the *trans*-N_2_CP system is highly diatropic, and **418a** showed the pyrrolic protons downfield between 8.44 and 8.55 ppm, while the inner CH resonances were observed upfield at −4.34 and −4.36 ppm and the NHs appeared at −2.73 and −3.21 ppm. *trans*-N_2_CPs **418** reacted with copper(II) acetate to give good yields of copper(III) organometallic derivatives **419**. Reaction of **417** with five equivalents of thiophenol gave doubly N-confused isophlorin **420** in 11% yield (Scheme 66) [256]. In the presence of air, or on standing over alumina, oxidation took place to give *trans*-N_2_CP dithioether **421**. Isophlorin **420** reacted with copper(II) acetate under aerobic conditions to give copper(III) complex **422**.

**Scheme 67:**
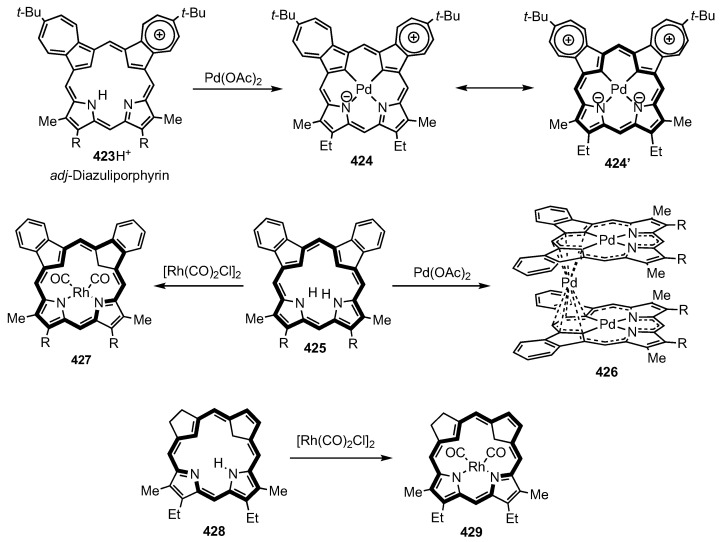
Metalation of *adj*-diazuliporphyrins, *adj*-dicarbaporphyrins and a related dicarbachlorin.

*adj*-Diazuliporphyrins **423** were isolated as monoprotonated forms as the corresponding free bases were unstable [257]. Reaction with palladium(II) acetate in refluxing acetonitrile gave palladium(II) complex **424** in 26% yield (Scheme 67). This polar organometallic complex can be represented as a series of dipolar or tetrapolar canonical forms. The X-ray crystal structure for **424** revealed that the porphyrinoid skeleton was slightly saddled. The proton NMR spectrum of **424** showed the *meso*-protons downfield at 7.9 (1H), 8.8 (2H) and 10.0 (1H) ppm, suggesting that this derivative is weakly aromatic. The aromatic properties of **424** can be attributed to resonance contributors such as **424′** that incorporate 18π electron delocalization pathways [257]. Reaction of *adj*-dicarbaporphyrin **425** with palladium(II) acetate resulted in the formation of a remarkable tripalladium sandwich complex **426** (Scheme 67) [245]. The X-ray crystal structure showed that the complex consisted of a palladium(IV) metallocene-type structure with *η*^5^-coordination to two palladium(II) dicarbaporphyrin dianions. The individual porphyrinoid units are planar and lie parallel to one another. The remarkable stability of this palladium(IV) derivative shows that dicarbaporphyrinoid systems are capable of stabilizing unusual oxidation states. Reaction of **425** with [Rh(CO)_2_Cl]_2_ gave rhodium(I) complex **427** [154]. The related dicarbachlorin **428** similarly afforded rhodium(I) complex **429** [154,258].

**Scheme 68:**
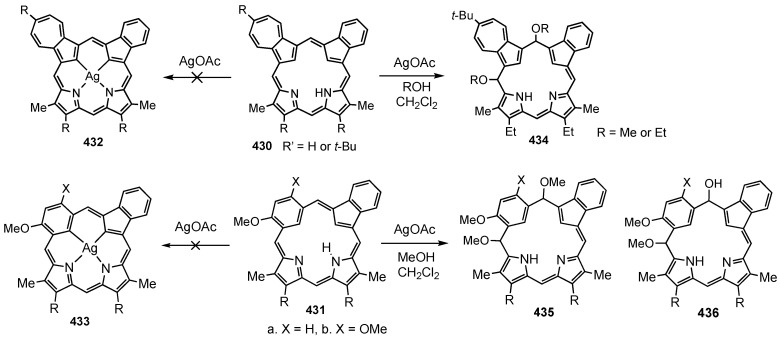
Selective oxidation of carbaazuli- and carbabenziporphyrins.

Carbaazuliporphyrins **430** [87] and carbabenziporphyrins **431** [246] both have three hydrogens within the macrocyclic core and are potentially trianionic ligands. However, attempts to prepare silver(III) derivatives **432** or **433** were unsuccessful (Scheme 68). Reaction of **430** with silver(I) acetate in methanol or ethanol instead selectively afforded nonaromatic oxidation products **434a,b** [87]. These dialkoxy derivatives were isolated as single diastereomers, although the precise stereochemical outcome was not determined. Carbabenziporphyrins **431** also gave selective oxidation reactions with silver(I) acetate in methanol-dichloromethane, and nonaromatic products **435** with two *meso*-methoxy substituents were isolated [246]. Again, the reactions were stereospecific in that only one diastereomer could be identified. A minor hydroxy-derivative **436** was also identified, presumably arising due to the presence of trace amounts of water.

**Scheme 69:**
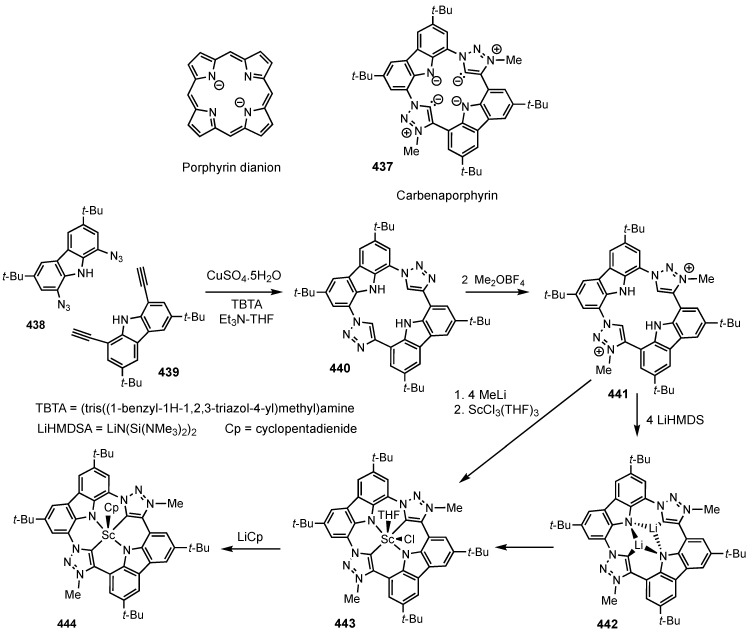
Carbenaporphyrins.

In a recent paper, a dicarbaporphyrinoid system incorporating *N*-heterocyclic carbenes was described (Scheme 69) [259]. In much the same way as porphyrins act as dianionic ligands, carbenaporphyrins **437** have a similar core arrangement that can potentially behave in the same way, albeit while generating organometallic derivatives. Copper-catalyzed alkyne-azide cycloaddition of a 1.8-diazidocarbazole **438** with a 1.8-diethynylcarbazole **439**, an example of a double click reaction, gave macrocycle **440** in 52% yield. Alkylation with Meerwein’s reagent quantitatively generated dication **441** as a bis(tetrafluoroborate) salt. Deprotonation of **441** with four equivalents of lithium hexamethyldisilazide gave a dilithium complex **442** that is equivalent to the target structure **437**. Transmetalation with scandium trichloride in THF gave a scandium carbenaporphyrin complex **443** that could be characterized by X-ray crystallography. Treatment of **443** with lithium cyclopentadienide afforded the corresponding cyclopentadienyl complex **444** as an orange solid [259]. Although the versatility of carbenaporphyrin ligands has yet to be demonstrated, this system has the potential to further extend the applications of dicarbaporphyrinoid systems.

## 11. Tri- and Tetracarbaporphyrins

**Figure 11:**
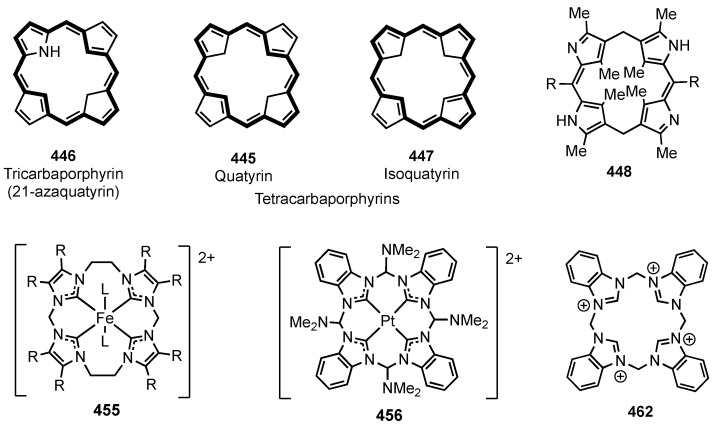
Tri- and tetracarbaporphyrinoids.

In principle, replacement of three or four of a porphyrin’s nitrogens with carbons would give tri- and tetracarbaporphyrins (Figure 11) [23,260,261,262]. However, these types of bridged annulene structure are presently unknown, although their significance has been discussed for many years [260]. The theoretical importance of tetracarbaporphyrin (quatyrin) **445** was appreciated by Vogel, who used this structure are a starting point when planning the synthesis of tetraheteroporphyrin dications and porphyrin isomers [263,264,265,266,267]. Unfortunately, attempts to synthesize quatyrin and related structures such as **446** and **447** have so far been unsuccessful [268]. Tri- and tetracarbaporphyrins **445**–**447** have been assessed using DFT and NICS calculations and the results show that quatyrin is planar and strongly aromatic [261]. However, it is worth noting that dicarbaporphyrins are much less stable than monocarbaporphyrins, and stability issues may plague further work in this area. Other structures with a porphyrin-type framework and four internal carbons can be considered and there has been some success in synthesizing macrocycles of this type. However, this possibility has not yet been applied to N-confused porphyrinoids. Doubly N-confused calix[4]pyrroles have been reported, but attempts to prepare quadruply N-confused calixphyrin **448** were unsuccessful [269].

**Scheme 70:**
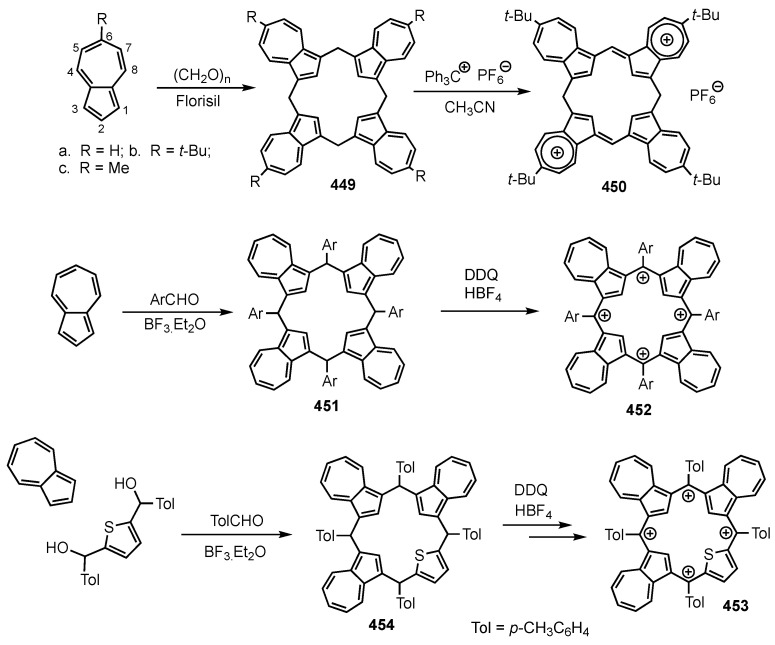
Calixazulenes and related cationic species.

The carbon skeleton for quatyrin is present in calix[4]azulenes **449**, which can be prepared by reacting azulenes with paraformaldehyde in the presence of florisil (Scheme 70) [97,98], and these macrocycles show interesting supramolecular interactions [270,271,272]. As noted earlier, azulene favors electrophilic substitution at the 1,3-positions and can substitute for pyrrole in the construction of porphyrinoid macrocycles. Treatment of **449b** with triphenylcarbenium hexafluorophosphate afforded a partially conjugated dication **450** that corresponds to a dihydroquatyrin [98]. This species was dark blue in solution and gave a strong absorption at 616 nm in its UV-vis spectrum. Oxidation of tetraarylcalix[4]azulenes **451** with DDQ in the presence of tetrafluoroboric acid gave the tetraazuliporphyrin tetracations **452** [273]. Although these tetracations might be considered to be didehydroquatyrins, DFT calculations demonstrate that they have severely distorted conformations and the macrocycles are not fully conjugated. A similar triazuli-thiaporphyrinoid **453** has also been reported [274]. Condensation of azulene with a thiophene dicarbinol and *p*-tolualdehyde in the presence of boron trifluoride etherate gave **454** as a diastereomeric mixture. Oxidation with DDQ and addition of 20 equivalents of HBF_4_.Et_2_O gave the nonaromatic tetracation **453**. Tetraazuliporphyrinoids have not been metalated in the core, although cluster complexes with the seven-membered rings have been reported [275].

**Scheme 71:**
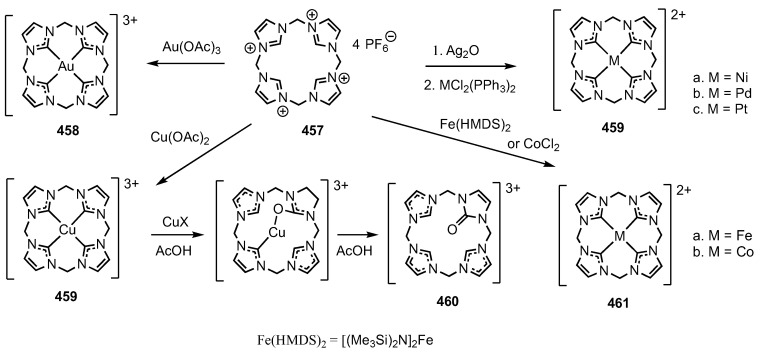
Metalation of a tetraimidazolyl system.

Although tetracarbaporphyrin ligands are not presently known, cyclic *N*-heterocyclic carbenes readily form organometallic derivatives. Macrocycles constructed from four imidazolium subunits have been reported and these form metalated derivatives such as **455** and **456** (Figure 11) [276,277,278,279]. These systems have a similar coordination framework to carbaporphyrins. Tetraimidazolyl tetracation **457** reacted with gold(III) acetate to give gold(III) trication **458** [280], while reaction with copper(II) acetate afforded copper(III) complex **459** (Scheme 71) [281]. Reaction of the latter complex with copper(I) salts and acetic acid, followed by demetallation, gave imidazolone trication **460**. Silver and gold cluster complexes of **457** were also reported. Reaction of **457** with bis[bis(trimethylsilyl)amido]iron(II) gave iron(II) complex **461a** [282,283,284,285], while treatment with cobalt(II) chloride afforded **461b** [286]. Both of these complexes initially reacted with O_2_ to give dioxygen complexes. At room temperature, the iron system afforded a μ-oxo dimer, while the cobalt complex generated a μ-peroxy species. Metalation of the related tetrabenzo-ligand **462** [280,287] (Figure 11) and boron-bridged analogues [288,289,290,291] have also been investigated.

## 12. Contracted Carbaporphyrinoids

**Figure 12:**
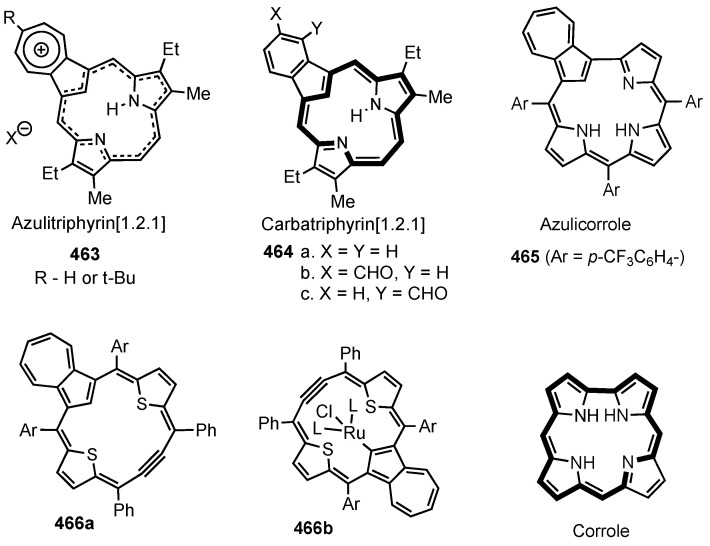
Examples of contracted carbaporphyrinoids and the structure of corrole.

Carbaporphyrinoid systems with smaller rings are known, including azulitriphyrin[1.2.1]s **463** [112,292], carbaporphyrins[1.2.1] **464** [112], azulicorroles **465** [293,294], and ethynyl-linked azuliporphyrinoid **466a** [295] (Figure 12). Some metalation studies have been performed on contracted systems, including the formation of ruthenium(II) complex **466b**, although work in this area is still in its early stages. Technically, these systems fall outside of the primary focus for this review, but the reactivity of these structures is clearly relevant.

**Scheme 72:**
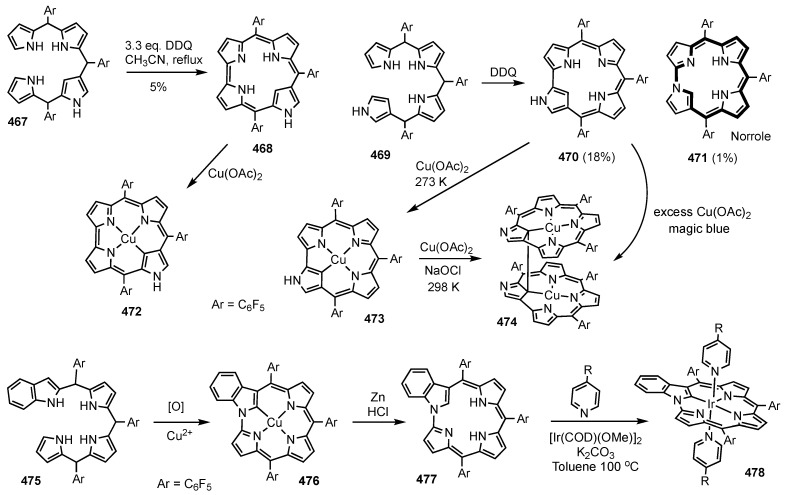
Metal complexes of N-confused and neo-confused corroles.

Corroles (Figure 12) are contracted porphyrins with only three bridging carbons. These have a more crowded cavity but retain aromatic character and act as trianionic ligands [296,297,298,299,300]. Furuta investigated the metalation of N-confused and neo-confused corroles (Scheme 72) [301,302]. N-confused bilane **467** was oxidatively cyclized with 3.3 equivalents of DDQ in acetonitrile to give N-confused corrole **468** in 5% yield. Isomeric bilane **469** similarly afforded N-confused corrole **470** in 18% yield but in this case a second corrole isomer **471** was generated in 1% yield [301]. Both N-confused corroles favored tautomers with an external NH. Neo-confused corrole **471**, named norrole, has a direct link between a pyrrole nitrogen and an adjacent pyrrole unit [301]. Norrole exhibits some diatropic characteristics, and the proton NMR spectrum showed the inner CH as an upfield resonance at 1.21 ppm. N-confused corrole **468** reacted with copper(II) acetate to give copper(III) complex **472**, and **470** similarly gave a related copper(III) derivative **473** when the reaction was carried out at 273 K. Although both of the copper(III) complexes are stable, **473** dimerizes in the presence of excess copper(II) acetate or in the presence of the oxidant magic blue to give **474**. This complex is linked via the internal carbon atoms. Oxidative cyclization of bilane **475** incorporating an indole unit with chloranil and copper(II) acetate afforded copper(III) benzonorrole **476** in 68% yield [302]. Reductive demetalation with zinc-hydrochloric acid produced free-base benzonorrole **477** in 94% yield. The X-ray structure of the copper(III) complex showed that the tetrapyrrolic unit had a nearly planar conformation. Reaction of benzonorrole **477** with [Ir(COD)(OMe)]_2_, 4-substituted pyridines, and potassium carbonate in toluene gave a series of near-infrared phosphorescent iridium(III) complexes **478** [303]. These derivatives have two axial pyridine ligands, but otherwise the macrocycle is near planar.

**Scheme 73:**
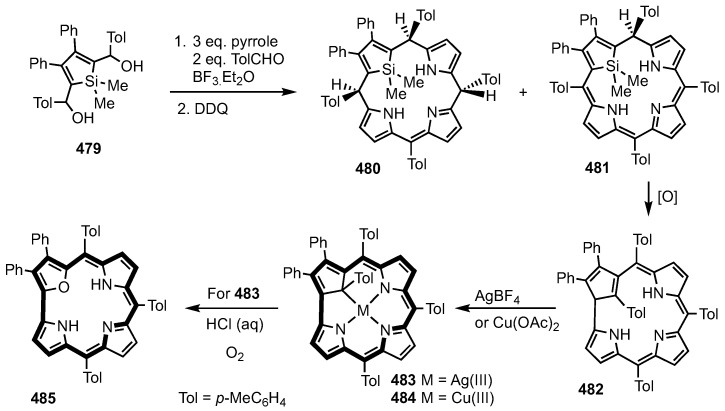
Silver(III) and copper(III) complexes of carbacorrole.

In an attempt to synthesize silaporphyrins, silole dicarbinol **479** was reacted with pyrrole and *p*-tolualdehyde in the presence of BF_3_.Et_2_O, and following oxidation with DDQ, two partially oxidized macrocycles **480** and **481** were isolated [304]. Further oxidation of **481** failed to give a fully conjugated silaporphyrin but instead afforded a low yield of nonaromatic carbacorrole **482**. Reaction of **482** with silver(I) tetrafluoroborate or copper(II) acetate gave silver(III) complex **483** and copper(III) derivative **484**, respectively (Scheme 73) [304]. Metal insertion was associated with tautomerization to give fully conjugated carbacorrole species. The proton NMR spectra for **483** and **484** showed that these complexes are strongly diatropic, and the protons on the internal tolyl substituents are shifted upfield. For example, solutions of silver(III) complex **483** in CDCl_3_ at 180 K showed the *o*-tolyl protons at 4.46 ppm. Silver(III) complex **483** reacted with aqueous HCl in the presence of O_2_ to give oxacorrole **485**. During the course of this reaction, the interior benzylic unit and the silver(III) cation are lost.

**Scheme 74:**
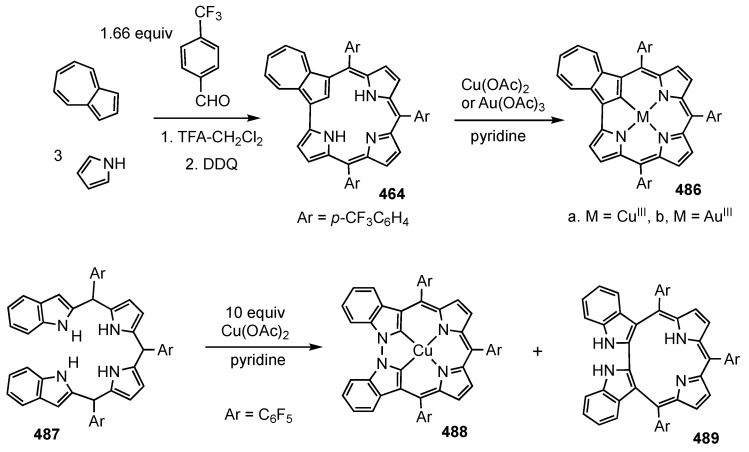
Copper(III) and gold(III) complexes of carbacorrole analogues.

Azulicorrole **465** was obtained in low yield by condensing azulene, pyrrole and 4-trifluoromethylbenzaldehyde in 10% TFA-CH_2_Cl_2_, followed by oxidation with DDQ (Scheme 74) [293]. Azulicorrole reacted with copper(II) acetate and gold(III) acetate to give metalated derivatives **486a,b** in 89% and 32% yield, respectively [293]. The X-ray structure for **464** showed that the azulene ring was tilted ca. 40° relative to the remaining macrocyclic plane, but as might be expected copper(III) complex **486a** was relatively planar. Bilane **487** with two terminal indole units was cyclized with 10 equivalents of copper(II) acetate to give copper(III) complex **488** together with 2.2′-biindole-linked macrocycle **489** in 12% and 18% yield, respectively [305]. The proton NMR spectrum of **488** indicates that there is an 18π electron delocalization pathway in the complex. Attempts to demetalate **488** with zinc dust in TFA-acetonitrile-CH_2_Cl_2_ to form the parent porphyrinoid were unsuccessful and resulted in the structure being converted back into bilane **487**.

**Scheme 75:**
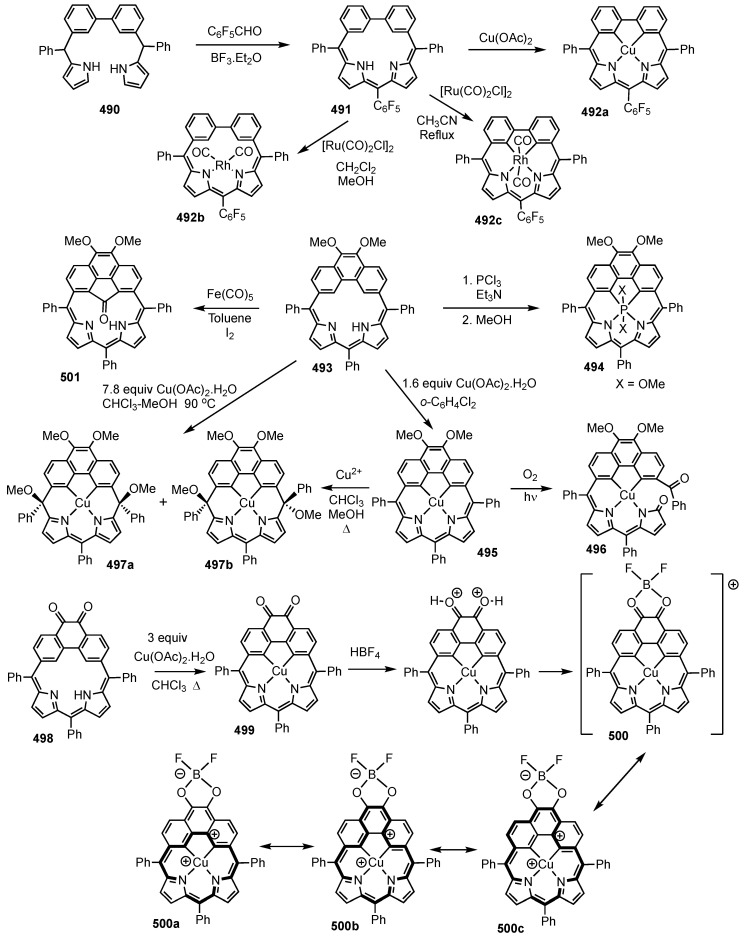
Organometallic chemistry of dibenzicorrole and phenanthriporphyrins.

**Scheme 76:**
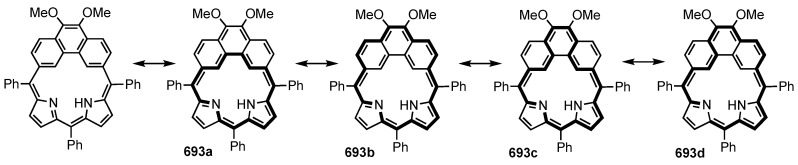
Resonance contributors of phenanthriporphyrin with 16 or 20π electron circuits.

Dicarbacorroles with a biphenyl unit or a phenanthrene moiety have been reported [306,307,308]. A bilane-like intermediate **490** incorporating a biphenyl unit was cyclized with pentafluorobenzaldehyde and BF_3_·Et_2_O and, following oxidation with DDQ, dibenzicorrole **491** was obtained in 10% yield (Scheme 75) [306]. The X-ray crystal structure of **491** showed that the benzene rings were tilted by 19.52° and 20.06° relative to the mean macrocyclic plane. Dibenzicorrole **491** reacted with copper(II) acetate to give organometallic copper(III) complex **492a** in 90% yield. Very recently, **491** was shown to react with [Rh(CO)_2_Cl]_2_ in dichloromethane-methanol to give rhodium(I) complex **492b** [307]. However, when the reaction was performed in refluxing acetonitrile, a rhodium(III) organometallic derivative **492c** was generated. A structurally related phenanthrene-containing system **493** was prepared in the same way (Scheme 75) [308,309]. The authors named this compound phenanthriporphyrin, but structurally the system is a dicarbacorrole. The proton NMR spectrum for **493** showed the external pyrrolic protons upfield as two 2H doublets at 5.24 and 5.59 ppm, while the inner CH protons were shifted downfield to 16.70 ppm. The external phenanthrene proton resonances were also relatively upfield, appearing at 5.94 and 6.94 ppm. These results are consistent with the macrocycle having a moderate paratropic ring current. The antiaromatic nature of **493** can be ascribed to the presence of 16π- and 20π-electron delocalization pathways shown in bold for resonance contributors such as **493a**–**d** (Scheme 76) [309]. Phenanthriporphyrin **493** reacted with phosphorus trichloride and triethylamine, followed by treatment with methanol in the presence of air, to give phosphorus(V) complex **494** [309]. This species retains the antiaromatic characteristics of the parent ligand. Reaction of **493** with 1.6 equivalents of copper(II) acetate gave antiaromatic copper(III) complex **495** [310]. Regioselective photolytic cleavage of **495** in the presence of molecular oxygen gave copper(III) phenanthribilinone **496**. When **493** was reacted with 7.8 equivalents of Cu(OAc)_2_ in chloroform-methanol, a diastereoisomeric mixture of dimethoxy derivatives **497a,b** was generated. Phenanthriquinone **498**, which can be prepared by demethylation of **493** with boron tribromide or sulfuric acid [311], also reacted with copper(II) acetate to give copper(III) complex **499**. Reaction with fluoroboric acid afforded BF_2_ complex **500**. Although **498** and **499** are nonaromatic, boron difluoride cation **500** has aromatic character. This can be rationalized as being due to canonical forms such as **500a**–**c** with 14 or 18π electron circuits. Phenanthriporphyrin **493** reacted with Fe(CO)_5_ and I_2_ to give keto-derivative **501**. It was proposed that this reaction involved the intermediacy of an iron(II) organometallic complex [312].

**Scheme 77:**
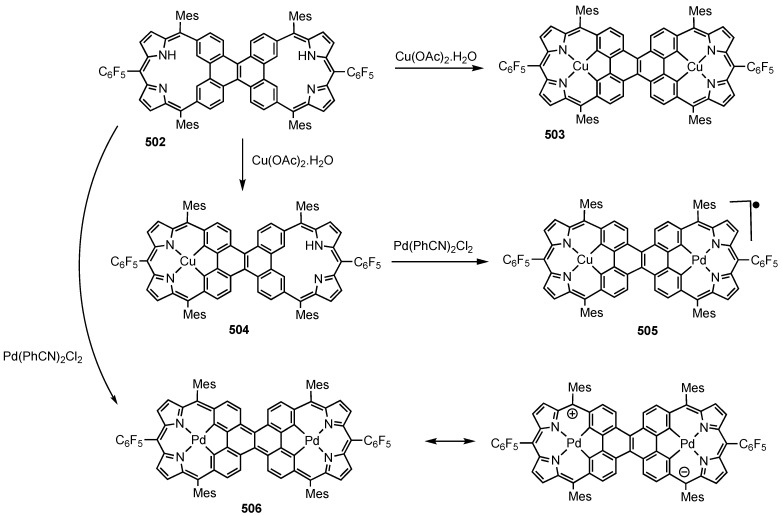
Bimetallic complexes of carbaporphyrinoid dimers.

Porphyrinoids with two interlinked phenanthriporphyrin units have been described (Scheme 77) [313,314]. Diporphyrinoid **502** reacted with copper(II) acetate to give a bis-copper(III) complex **503**. A monocopper(III) complex **504** could also be isolated and this reacted with Pd(PhCN)_2_Cl_2_ to give the mixed Cu^III^-Pd^II^ complex **505** as a stable radical species. When **502** was reacted with Pd(PhCN)_2_Cl_2_, an unusual bis-palladium complex **506** was formed. Bis-porphyrioid **502** and bis-copper(III) complex **503** are antiaromatic, as judged by proton NMR spectroscopy, but the upfield shifts of the external protons are drastically reduced for dipalladium complex **506**. Bond length analysis indicates that the complex has quinoidal character, indicating that the π-systems of the individual macrocycles are strongly interacting.

**Scheme 78:**
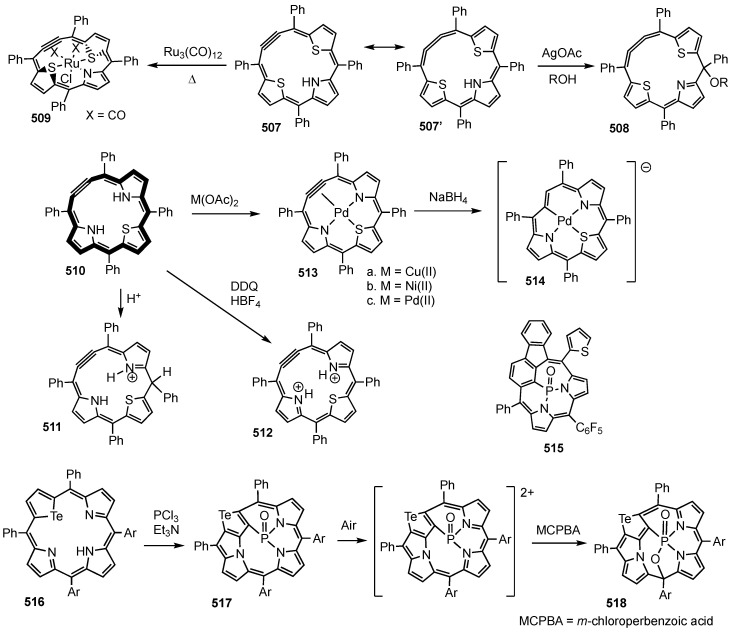
Metalated derivatives of contracted carbaporphyrinoids.

Dithiaethyneporphyrin **507** has an acetylene unit in place a pyrrole ring (Scheme 78) [315]. The four-carbon bridge facilitates conjugation, and this system can be represented as acetylene-linked structure **507**, or the cumulene resonance contributor **507′**, both having 18π electron delocalization pathways. Oxidation with silver acetate in an alcohol solvent gave nonaromatic alkoxyphlorins **508**, while metalation with Ru_3_(CO)_12_ in refluxing chlorobenzene afforded ruthenium complex **509**. A related monothiatriphyrin **510** (Scheme 78) is also aromatic, but protonation affords a nonaromatic cation **511** [316]. Oxidation of **510** with DDQ in the presence of fluoroboric acid gave an aromatic dication **512**. Although both **510** and **512** are aromatic, the π-conjugation pathways are quite different. Porphyrinoid **510** acts as a dianionic ligand and reacts with copper(II) acetate to give a copper(II) complex **513a** that has significant *η*^2^-interactions with the triple bond (Scheme 78). Similar complexes **513b,c** were obtained when **510** was reacted with nickel(II) or palladium(II) acetate. Reduction of **513c** with sodium borohydride gives an aromatic anion **514** in which the palladium(II) is directly bonded to a carbon atom.

Contracted carbaporphyrinoids can give rise to organophosphorus complexes such as **515** [317]. Reaction of telluraporphyrin **516** with phosphorus trichloride in triethylamine, followed by air oxidation, led to insertion of phosphorus and inversion of the tellurophene ring to give the carbaporphyrinoid complex **517** [318]. Oxidation with *m*-chloroperbenzoic acid (MCPBA) and reaction with water afforded a further oxidized nonaromatic product **518**.

## 13. Expanded Carbaporphyrinoids

**Figure 13:**
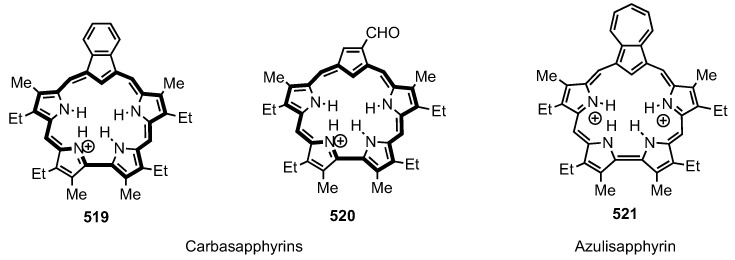
Carbasapphyrins.

**Scheme 79:**
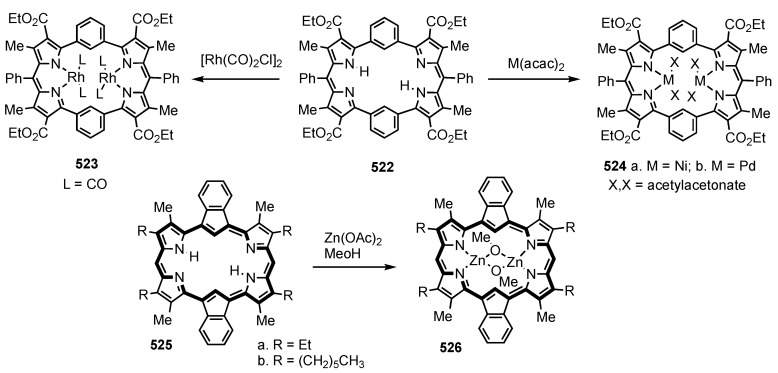
Coordination complexes of dibenzi- and dicarba-amethyrins.

Expanded carbaporphyrinoid systems have also been investigated but these diverge a great deal from the systems discussed above and will not be covered in detail. Early examples of expanded carbaporphyrins are carbasapphyrins **519** and **520**, and azulisapphyrin **521** (Figure 13) [319,320,321], but no metalation studies were conducted. It is worth noting that pentapyrrolic sapphyrins were the first expanded porphyrins to be discovered [322] and they continue to be widely investigated [323,324,325]. Dibenziamethyrin **522** was shown to react with Rh_2_(CO)_4_Cl_2_ in benzene to give high yields of bis-rhodium(I) complex **523** and reactions with nickel(II) or palladium(II) acetylacetonate gave bis-nickel(II) complex **524a** and bis-palladium(II) derivative **524b**, respectively (Scheme 79) [326,327]. Similarly, dicarbaamethyrin **525** reacted with zinc acetate in methanol to give the bridged bis-zinc complex **526** [328]. However, organometallic derivatives for these systems have not been identified.

**Scheme 80:**
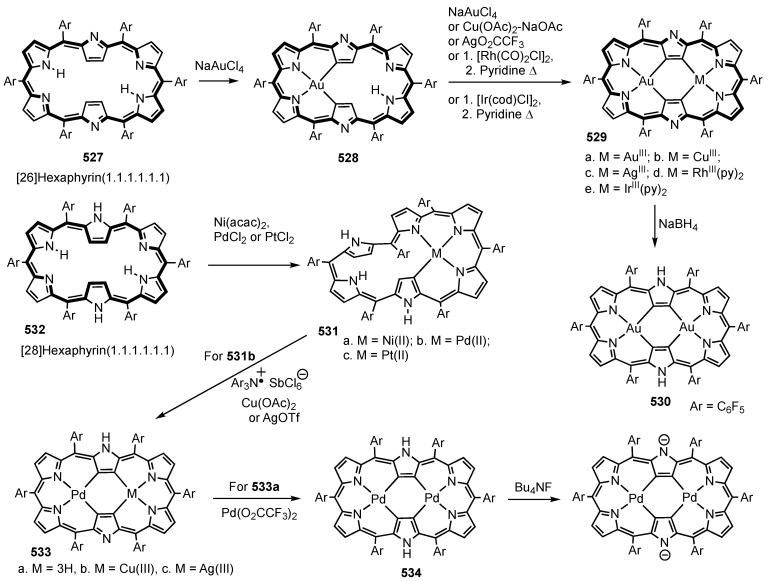
Organometallic derivatives of hexaphyrins(1.1.1.1.1.1).

Expanded porphyrinoid systems often possess inverted heterocyclic rings that place CH units within the macrocyclic cavity, and this may allow the formation of organometallic derivatives. These structures are in essence carbaporphyrinoid-type systems, but only select examples will be presented. Hexaarylhexaphyrins **527** are particularly versatile organometallic ligands that often place four CH units within the macrocycle (Scheme 80) [329,330,331,332]. This provides two binding pockets that resemble dicarbaporphyrinoid structures. Hexaphyrin **527** reacted with NaAuCl_4_ to give a mixture of the mono-gold(III) **528** (16%) and the bis-gold(III) complexes **529a** (14%) [329]. Reduction of **528** or **529a** with sodium borohydride gave the related antiaromatic [28]hexaphyrin complexes **530**. This chemistry has been applied to preparation of mixed complexes with Ag(III)-Au(III), Cu(III)-Au(III), Rh(III)-Au(III), and Ir(III)-Au(III) (**529b**–**e**) [329,330,331,332,333]. Nickel(II), palladium(II) and platinum(II) complexes **531** were obtained by reacting [28]hexaphyrin(1.1.1.1.1.1) **532** with Ni(acac)_2_, PdCl_2_ or PtCl_2_, respectively [334]. The chiral Mobius aromatic palladium(II) complex **531b** has been resolved to give the individual enantiomers by using HPLC on a chiral stationary phase [335]. Treatment of **531b** with tris(4-bromophenyl)aminium hexachloroantimonate induced a molecular topology change to give the Hückel aromatic complex **533a**. Reaction of **533a** with copper(II) acetate gave Pd(II)-Cu(III) [28]hexaphyrin complex **533b** in 90% yield, while reaction with silver triflate in acetonitrile afforded Pd(II)-Ag(III) complex **533c** in 93% yield [336]. Treatment of **533a** with Pd(OCOCF_3_)_2_ generated the aromatic bis-palladium(II) complex **534**, and this was readily deprotonated with tetrabutylammonium fluoride to produce the corresponding dianion (Scheme 80) [337].

**Scheme 81:**
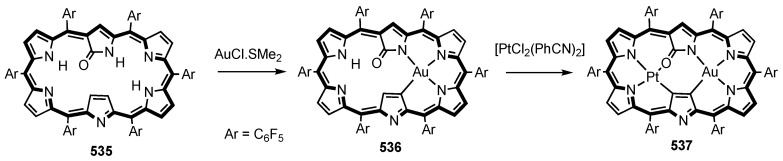
Organometallic derivatives of doubly N-confused hexaphyrins.

**Scheme 82:**
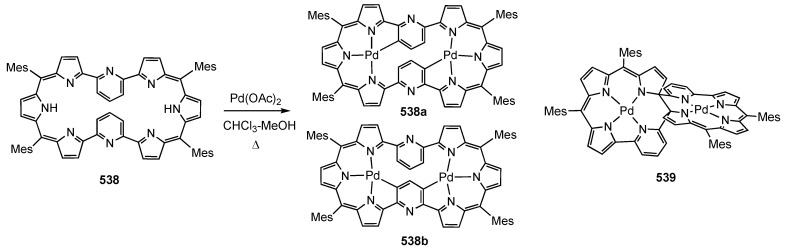
Palladium complexes of a dipyrioctaphyrin.

**Scheme 83:**
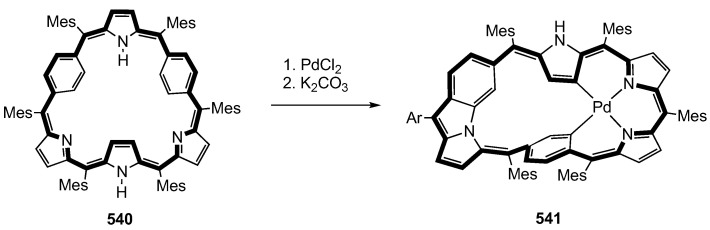
Synthesis of a palladium(II) complex of a dibenzihexaphyrin.

**Figure 14:**
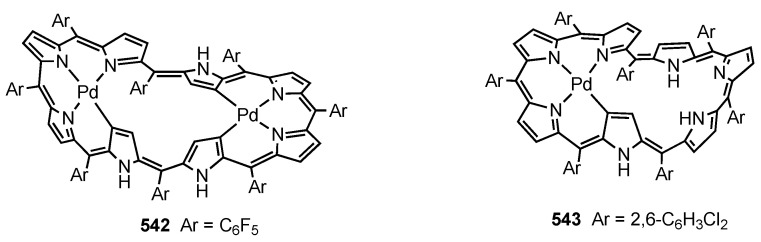
Möbius aromatic palladium(II) complexes of expanded porphyrins.

Reaction of AuCl.SMe_2_ with doubly N-confused hexaphyrin **535** gave the gold(III) complex **536**, and further treatment with PtCl_2_(PhCN)_2_ afforded the mixed Pt(II)-Au(III) complex **537** (Scheme 81) [338]. In another intriguing study, reaction of palladium(II) acetate with dipyrihexaphyrin **538** gave three dipalladium complexes **538a,b** and **539** (Scheme 82) [339]. Structure **539** is not an organometallic derivative but has an unusual interlocked structure with two pyricorrole-like components. Many examples of expanded porphyrins with *m*-phenylene or *p*-phenylene units have been described and these may also give organometallic derivatives. For example, dibenzihexaphyrin **540** reacted with palladium(II) chloride and potassium carbonate to give Möbius aromatic palladium(II) complex **541** (Scheme 83) [340]. Other examples include the Möbius aromatic palladium(II) porphyrinoids **542** and **543** (Figure 14) [341]. These examples illustrate some exciting examples of organometallic expanded porphyrinoids, but no attempt has been made to give comprehensive coverage of this area.

## 14. Related Systems

**Figure 15:**
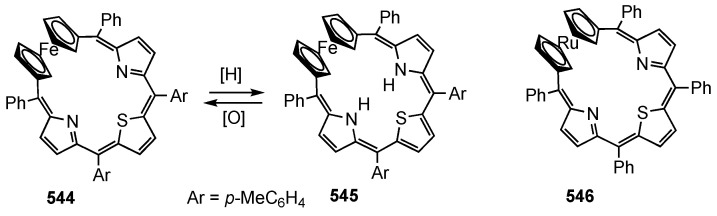
Metallocenoporphyrins.

Many other closely related systems with porphyrin-like frameworks have been investigated. Metallocenoporphyrins such as **544**–**546** (Figure 15) incorporate ferrocene or ruthenocene units in place of a pyrrole ring [342,343]. Surprisingly, the metallocene units facilitate conjugation within these macrocycles and they exhibit a degree of aromatic, or in some cases antiaromatic, character. This shows that π-electron delocalization can be transferred through the d-orbitals of the metallocene component.

**Scheme 84:**
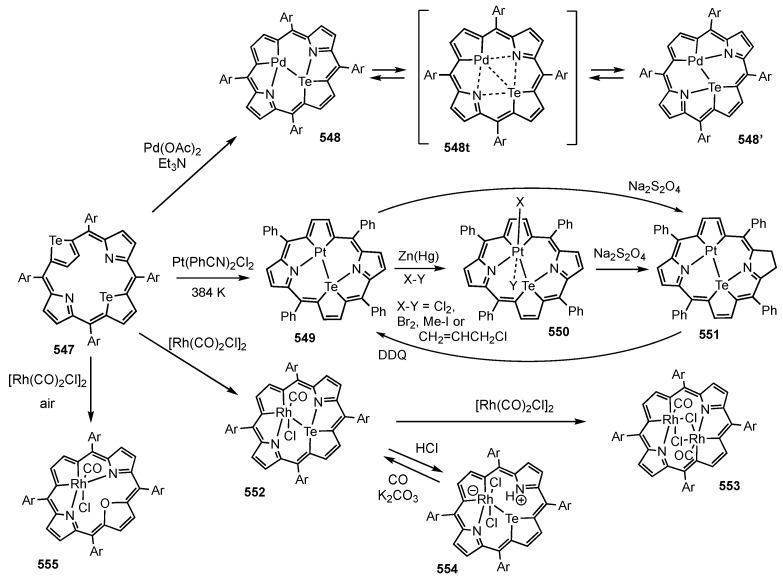
Synthesis of metallaporphyrins.

Another intriguing class of porphyrin-like structures have been prepared from 21,23-ditelluraporphyrins **547** (Scheme 84). Reaction of **547** with palladium(II) acetate and triethylamine gave derivative **548** where palladium(II) has replaced tellurium as one of the core atoms [344]. The new macrocycle can be viewed as a 21-pallada-23-telluraporphyrin, although the bonding interactions within the core are quite different from other porphyrinoids. X-ray crystallography shows that the Pd was covalently bound to only two neighboring atoms, nitrogen and tellurium, breaking the symmetry of the macrocycle. The proton NMR spectrum at 300 K appears to show a symmetrical aromatic structure, but many of the resonances split at 180 K. The results show that two equivalent structures, **548** and **548′**, rapidly interconvert a room temperature via a symmetrical transition state **548^t^**. Reaction of **547** with Pt(PhCN)_2_Cl_2_ at 384 K gave an analogous platinatelluraporphyrin **549** [345]. Reduction with zinc amalgam in the presence of Cl_2_, Br_2_, MeI or allyl chloride gave a series of Pt(IV) addition products **550**. When treated with sodium dithionite, **550** afforded the corresponding metallachlorin **551**, but this could be oxidized back to **549** with DDQ. Reaction of **547** with [Rh(CO)_2_Cl]_2_ in refluxing toluene gave rhodatelluraporphyrin **552** and dirhodaporphyrin **553** [346]. The dirhodaporphyrin macrocycle was relatively planar and the rhodium atoms were linked via two chloride bridges. When treated with HCl, **552** was converted to the zwitterionic complex **554**. Reaction of ditelluraporphyrin **547** with [Rh(CO)_2_Cl]_2_ in the presence of air led to loss of both tellurium atoms to afford oxarhodaporphyrin **555**. Hence, remarkable new organometallic derivatives can be generated within porphyrin-like frameworks.

**Scheme 85:**
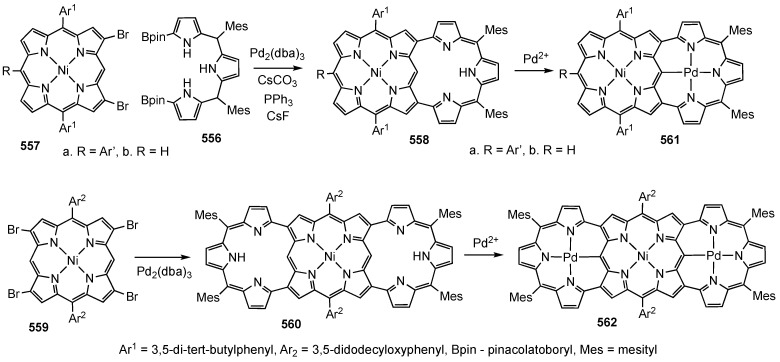
Porphyrin earrings.

**Figure 16:**
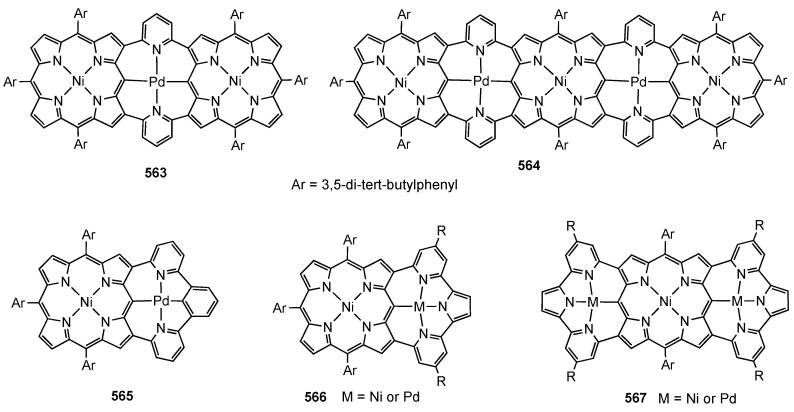
Metal complexes of porphyrins with appended carbaporphyrin-like loops.

Another variation on the theme are structures with carbaporphyrin-like cavities that are built onto porphyrin macrocycles [347,348]. A case in point are the so-called porphyrin earrings (Scheme 85). Palladium-catalyzed Suzuki−Miyaura coupling of diboryltripyrrane **556** with nickel(II) dibromoporphyrins **557a** or **557b** gave porphyrin “earrings” **558a** and **558b** in 32% and 20% yields, respectively [347]. It was possible to install two “ears” onto a porphyrin by coupling tetrabromoporphyrin **559** with two equivalents of **556** and double-earring porphyrin **560** was generated in 8% yield [347]. It was necessary to introduce 3,5-didodecyloxyphenyl substituents to increase the solubility of these structures. Although the porphyrin earrings have curved geometries, the newly introduced cavities have the same core atoms as monocarbaporphyrinoid systems. Both **557a** and **560** reacted with palladium(II) acetate to give the palladium(II) complexes **561** and **562**, respectively, in >90% yield. A number of related porphyrins have been reported [349,350] that bind nickel(II) and palladium(II) within the appended carbaporphyrin-like loop, including structures **563**–**567** (Figure 16).

## 15. Conclusions

The 16-atom core of carbaporphyrins has a CNNN binding pocket that can facilitate the formation of metalated derivatives. Indeed, the ordered cavities found in these porphyrinoid structures provide an intriguing environment to probe organometallic processes. These systems form complexes with many of the late transition metals and can stabilize higher oxidation states. The ligands can be profoundly altered by introducing a multitude of different subunits. Pyrrole units can be replaced by furan, thiophene, selenophene or tellurophene. More importantly, the subunit that places a carbon atom within the cavity can be an inverted pyrrole, furan or thiophene, or cyclopentadiene, indene, azulene, cycloheptatriene, inverted pyridine, pyrazole, benzene, naphthalene, and so on. Furthermore, macrocycles with two internal carbons are also easily accessible. These structural changes not only affect metalation processes but also the spectroscopic and chemical reactions for these ligands. Carbaporphyrinoids may be fully aromatic but in some cases, they are nonaromatic or antiaromatic. Some expanded carbaporphyrinoids can even take on twisted conformations that lead to Möbius aromatic or antiaromatic structures. The unprecedented structural diversity of carbaporphyrinoid systems has led to the discovery of a remarkable wealth of coordination architectures and highly usual reactivity. The organometallic complexes also have value in the design of catalytic systems, including catalysts for cyclopropanation reactions [123,351] and CO_2_ fixation [352]. In addition, medicinal applications of metallocarbaporphyrinoids as photosensitizers for photodynamic therapy have been noted [353,354]. This area continues to surprise and will no doubt lead to many further advances in the future.

## Figures and Tables

**Table 1:** Selected proton NMR chemical shifts (ppm) for palladium(II) carbaporphyrins.

	150	141	163	164
5,20-H	10.42	10.27	9.85	9.54
10,15-H	10.00	9.56	9.13	8.84
7,18-Me	3.49	3.33	3.16	3.05
21-Me	−4.46	−3.21	−2.18	−1.45

## Data Availability

Not applicable.
